# Accelerated Thioarylation of Arenes Using Lewis Acid and Lewis Base Dual Catalysis

**DOI:** 10.1021/acs.joc.6c00744

**Published:** 2026-05-18

**Authors:** Oluwajuwon A. M. Okunade, Pankaj K. Majhi, R. Nisha Khanizeman, Andrew Sutherland

**Affiliations:** † School of Chemistry, The Joseph Black Building, 3526 University of Glasgow, Glasgow G12 8QQ, U.K.; ‡ GSK Medicines Research Centre, Gunnels Wood Road, Stevenage SG1 2NY, U.K.

## Abstract

Biaryl sulfides are valuable motifs in pharmaceuticals and functional materials and thus require efficient synthetic methods that operate rapidly and under mild conditions. We recently reported a method for the synthesis of biaryl sulfides via the reaction of arenes with iron­(III)-activated saccharin-based thioaryl reagents. However, this approach proved to be less effective for electron-deficient thioaryl groups, which required prolonged reaction times and more forcing conditions. Here, we present an improved procedure featuring a dual catalytic system in which diphenyl selenide acts as a Lewis base cocatalyst to accelerate iron-catalyzed thioarylation at room temperature. This modification significantly enhances the reactivity of electron-deficient saccharin-based reagents, broadening the substrate scope and enabling late-stage functionalization of complex molecules such as natural products and pharmaceuticals. The synthetic utility of this dual catalytic thioarylation reaction is demonstrated with a concise synthesis of the HIV-1 reverse transcriptase inhibitor L-737,126 and late-stage functionalization of the painkiller naproxen.

## Graphical abstract

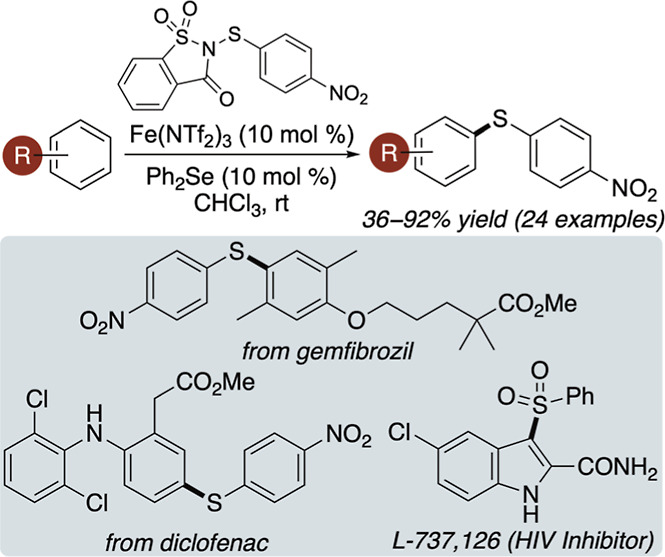

## Introduction

Biaryl sulfides represent an important class of organosulfur compounds with applications in organic synthesis, materials chemistry, and medicinal chemistry. In organic chemistry, as well as acting as intermediates for sulfoxides and sulfones,^1^ these are commonly used as functional handles such as sulfonium salts, which are utilized as electrophilic intermediates in various substitution reactions or as radical precursors in photoredox catalysis.^2,3^ The nucleophilicity of sulfur can be exploited in alkylation or acylation reactions, and the sulfur atom can stabilize adjacent carbocations and radicals, facilitating rearrangements or radical-based processes.^1,4^ In materials science, poly­(aryl)­sulfide derivatives have been prepared via palladium-catalyzed polycondensations, yielding conjugated sulfur-rich polymers with potential in electronic and optoelectronic devices.^5^ Furthermore, biaryl sulfide motifs are found in numerous natural products and pharmaceuticals, where the sulfur linkage can play a key role in modulating lipophilicity, stability, and binding interactions.^6^ Some examples are shown in Figure 1a and include the antiallergenic compound AZD4407 (**1**),^7^ the immunosuppressive agent azathioprine (**3**),^8^ and the antidepressant vortioxetine (**5**).^9^ As key intermediates in drug synthesis, biaryl sulfides are commonly oxidized to the sulfone-containing pharmaceutical, as exemplified by the synthesis of the HIV-1 reverse transcriptase inhibitor L-737,126 (**6**).^10^

**Figure 1:**
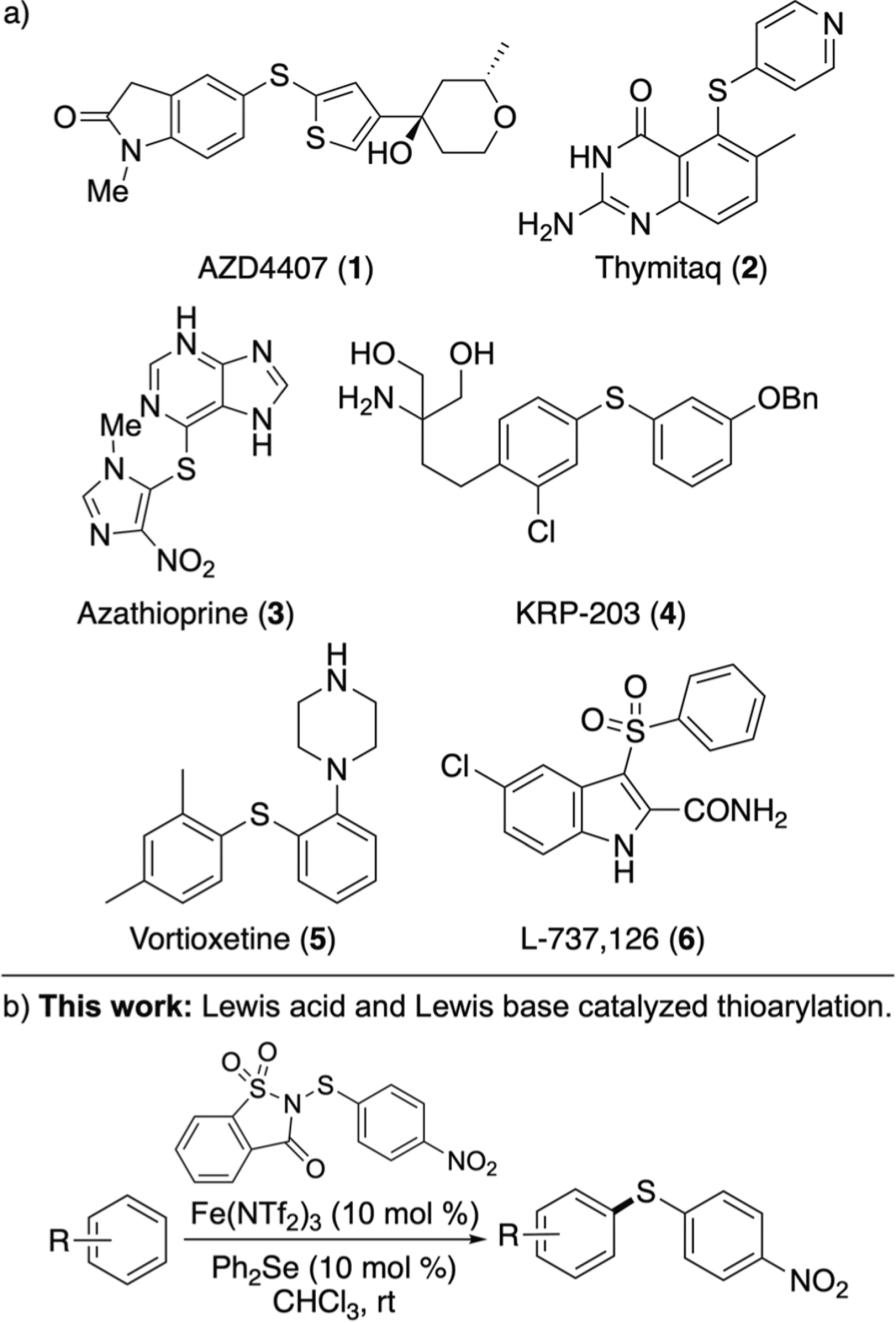
(a) Bioactive aryl thioethers. (b) This work: accelerated thioarylation using dual-catalyzed activation of saccharin-based reagents.

A variety of synthetic methods have been developed for the preparation of biaryl thioethers,^11^ including coupling reactions of organometallic reagents with electrophilic arylsulfur compounds^12^ or transition-metal-catalyzed reactions of aryl (pseudo)­halides with thiols or disulfides.^13^ Direct C–H thiolation methods have also emerged,^14^ such as thioarylation of arenes using electrophilic sulfoxonium salts^3^ and related approaches employing *N*-(arylthio)­succinimides as electrophilic partners.^15^ Activating commonly used *N*-(arylthio)­succinimides typically requires palladium catalysis^15a^ or strong Brønsted acids such as trifluoroacetic acid (TFA)^15a,15d,15e^ or triflic acid.^15f^ To circumvent the need for precious-metal catalysis or strong Brønsted acids, we recently reported a series of arene C–S bond-forming reactions that employ Lewis acid-catalyzed iron­(III) activation of electrophilic succinimide- and saccharin-based reagents.^16^ This work included the use of iron­(III) triflimide as a super Lewis acid to activate saccharin-derived thioarylation reagents for the synthesis of unsymmetrical biaryl sulfides.^17^ Although effective for transferring most thioaryl groups, this method proved less effective for strongly electron-deficient analogues, such as nitro-substituted variants, which required elevated temperatures and prolonged reaction times (up to 48 h). This suppressed reactivity also restricted the scope to highly activated arenes.

To address this limitation, we sought strategies to enhance the reactivity of saccharin-based thioarylation reagents, enabling access to nitro-substituted biaryl sulfides suitable for late-stage structural modification. Prior studies have shown that Lewis acid-catalyzed processes can be accelerated by Lewis bases, for example, the Gustafson group’s dual-catalytic system combining triflic acid with Lewis basic biaryl selenides for arene thiolation.^15f^ Inspired by this precedent, we proposed that a more general and faster iron-catalyzed thioarylation of saccharin reagents bearing strongly electron-deficient thiolating groups could be achieved through a dual-catalytic Lewis acid and Lewis base system. Here, we report the development of a rapid thioarylation reaction enabling the transfer of electron-deficient, nitro-substituted thioaryl groups using dual Lewis acid and Lewis base catalysis (Figure 1b). In addition to demonstrating a broader substrate scope and the utility of this dual-catalytic approach for late-stage, regioselective functionalization of biologically active molecules, we also showcase its application for the preparation of pharmaceutically relevant targets, including the HIV-1 reverse transcriptase inhibitor L-737,126 (**6**).

## Results and Discussion

Development of an accelerated thioarylation method began with an investigation of the reaction between 2-methylanisole (**7a**) and *N*-(4-nitrophenylthio)­saccharin (**8a**).^18^ As previously reported, catalysis at room temperature using only iron­(III) triflimide (10 mol %), generated in situ from iron­(III) chloride and the readily available ionic liquid [BMIM]­NTf_2_, required 23 h to give (4′-nitrophenyl)­(3-methyl-4-methoxyphenyl)­sulfane (**9a**) in 85% yield (Table 1, entry 1).^17^ A series of commercially available Lewis bases (10 mol %) were then evaluated under these conditions. As expected, sulfanes such as bis­(4-methoxyphenyl)­sulfane (entry 2) and 1-methoxy-4-(methylthio)­benzene (entry 3), previously shown to activate succinimide-based reagents,^19^ accelerated the reaction to 1 h while maintaining comparable yields of **9a**. In contrast, the use of *N*,*N*-diphenylthiourea (entry 4) and triphenylphosphine sulfide (entry 5) resulted in poor conversion and low yields of **9a** over similar reaction times.^20^ The fastest reaction was observed with diphenyl selenide (10 mol %) as the Lewis base (entry 6), which reached completion within 0.5 h and delivered **9a** in 85% yield. As diphenyl selenide was found to be the optimal Lewis base, this thioarylation was repeated on a larger scale (1 mmol), which gave **9a** in 82% yield after a reaction time of 0.75 h (entry 7). Finally, to demonstrate that both a Lewis acid and a Lewis base are required for this rate enhancement, the reaction was repeated in the absence of iron triflimide (entry 8). No reaction was observed after 2 h, confirming the dual-catalytic nature of the accelerated process.

**Table 1:**
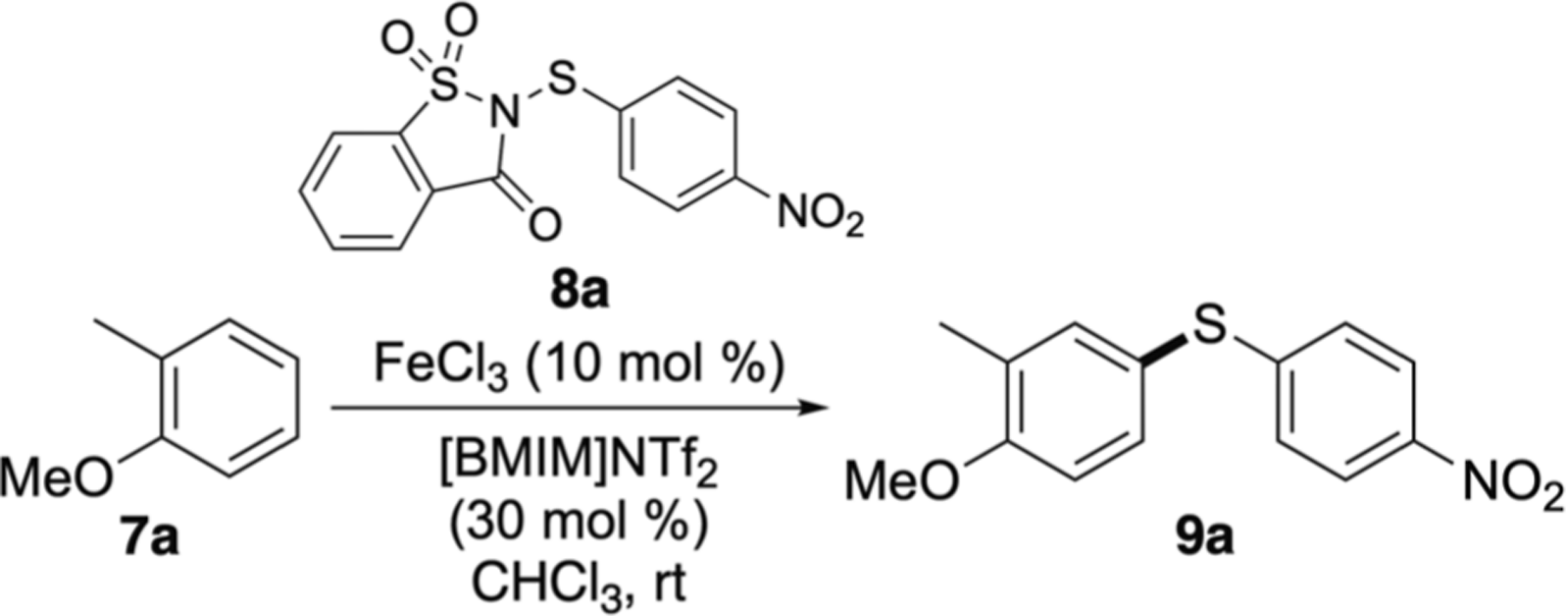
Examination of Lewis Bases for the Accelerated Thioarylation of 2-Methylanisole (**7a**)

entry	Lewis base (10 mol %)	time (h)	yield (%)^a^
1	----	23	85
2	(4-MeOPh)_2_S	1	82
3	(4-MeOPh)SMe	1	84
4	(PhNH)_2_C=S	1	11
5	Ph_3_P=S	1	8
6	Ph_2_Se	0.5	85
7^b^	Ph_2_Se	0.75	82
8^c^	Ph_2_Se	2	---

a Isolated yields.

b The reaction was performed on a 1 mmol scale.

c The reaction was done in the absence of iron­(III) triflimide.

On the basis of the results of the optimization study, a reaction pathway for the dual-catalyzed thioarylation reaction has been proposed (Scheme 1). Following formation of iron­(III) triflimide, the super Lewis acid initially activates *N*-(4-nitrophenylthio)­saccharin (**8a**). This activation enables its subsequent reaction with diphenylselenide to form a highly reactive selenium cation, which then undergoes rapid electrophilic aromatic substitution with the arene. In previous iron-catalyzed thioarylation studies, we have shown that while electron-rich biaryl sulfide products can accelerate these reactions by acting as a Lewis base through an autocatalytic process, electron-deficient biaryl sulfides are unable to participate in this way.^16a^ Consequently, the formation of electron-deficient products such as **9a** likely proceeds solely through the generation of the highly reactive selenium cation from diphenyl selenide.

**Scheme 1:**
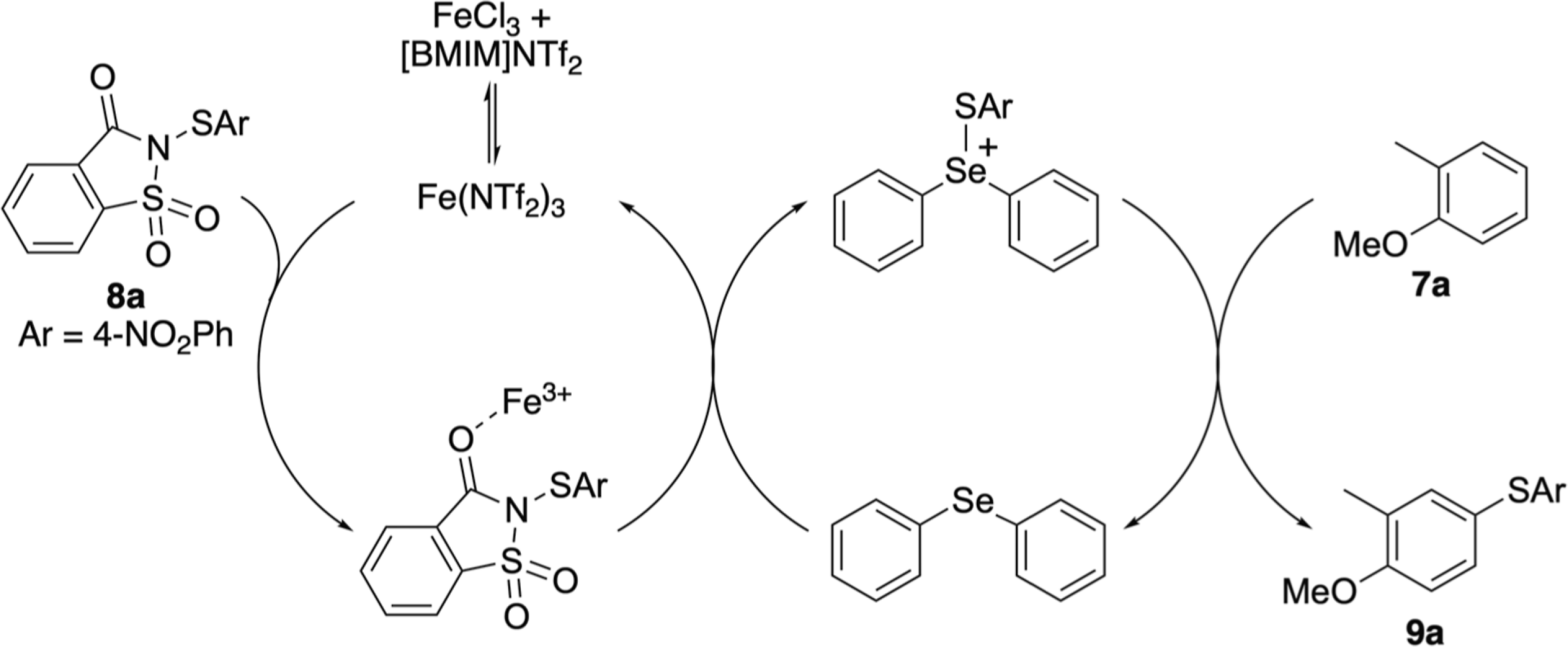
Proposed Reaction Pathway for the Dual-Catalyzed Thioarylation Reaction

Having identified diphenyl selenide as the optimal Lewis base, comparative studies were performed to examine the differences between an iron-only catalytic process versus the dual-catalyzed reaction. A range of highly activated arenes, as well as less reactive substrates, including some with deactivating substituents, were selected for this study. In all cases, the dual-catalytic process gave the desired products in similar or improved yields (Scheme 2). More importantly, all dual-catalyzed reactions proceeded at room temperature and were significantly faster than the iron-only process. For example, the reaction of highly activated substrates such as 1-methoxynaphthalene with *N*-(4-nitrophenylthio)­saccharin (**8a**) required heating to 40 °C and a reaction time of 4.5 h when using only iron­(III) triflimide (10 mol %). In contrast, upon addition of diphenyl selenide (10 mol %) using the dual-catalytic system, the reaction was complete after 1 h at room temperature. More significant differences were observed with less activated arenes. The iron-catalyzed thioarylation of 3-bromophenol required 40 °C and 48 h, whereas the dual-catalytic reaction furnished **9d** at room temperature in only 1.5 h. Similarly, formation of **9f** from mesitylene was complete after 2 h under dual catalysis, compared with 29 h at 40 °C using only iron triflimide. These results demonstrate that for all substrates and especially for less active arenes, the dual-catalyzed thioarylation reaction enables significantly accelerated reactions under mild conditions.

**Scheme 2:**
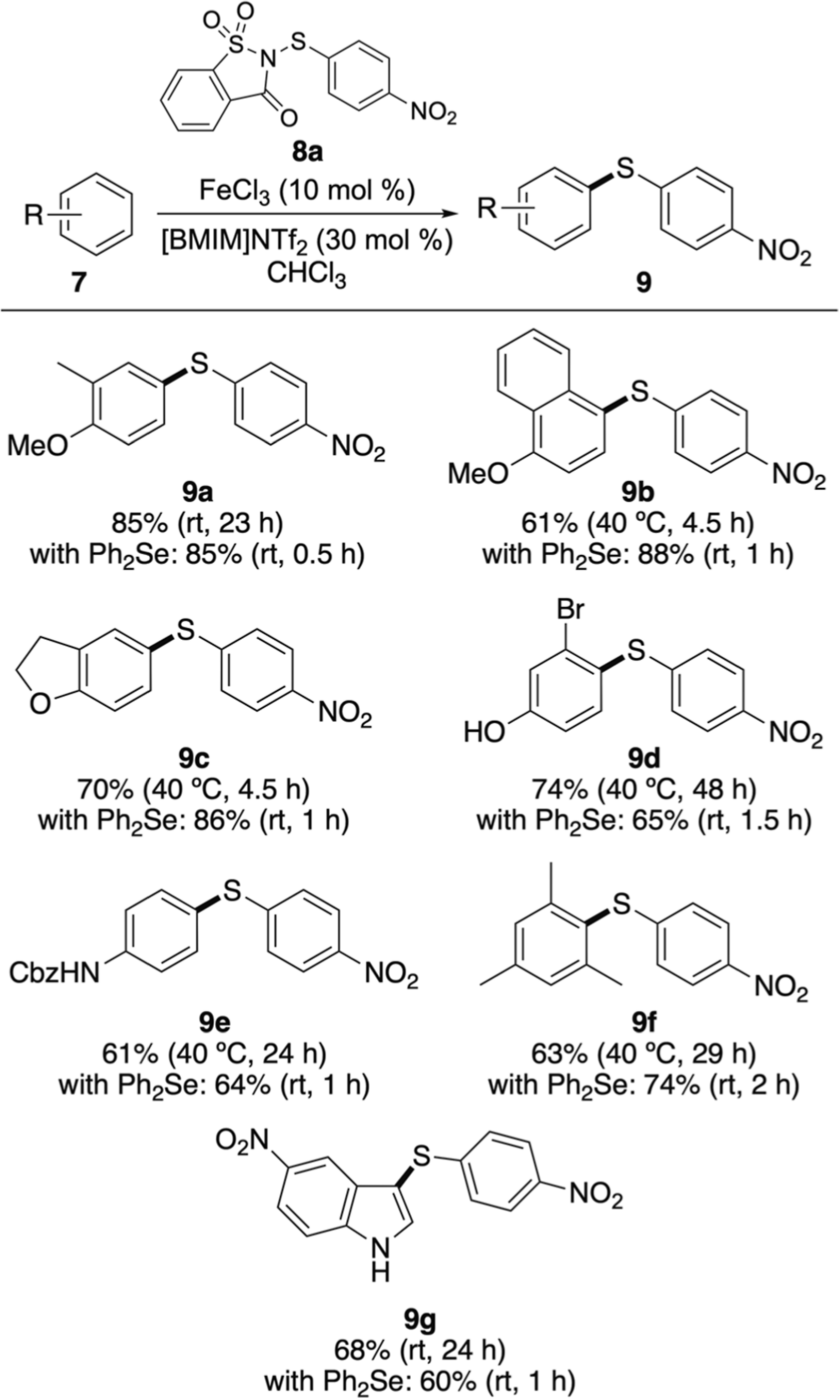
Comparison of the Iron-Catalyzed and Dual-Catalyzed Thioarylation Reactions^a^ ^,^ ^b^

a Isolated yields.
b For reactions with diphenylselenide, 10 mol % was used.

Having established the advantages of the dual-catalytic protocol, its application to new substrates, including compounds that exhibited little or no reactivity under the iron-only conditions, was evaluated (Scheme 3a). Anilines bearing either protecting groups (**7h**) or deactivating substituents (**7i** and **7j**) underwent efficient thioarylation, with all reactions reaching completion within 1.5 h to afford the corresponding biaryl sulfides **9h**–**9j** in 62–82% yields. A range of heterocycles (**7l**–**7n**) also proved compatible with the dual-catalytic system, delivering the desired products within 1 h. 1-(Phenylsulfonyl)­indole (**7l**), which showed low reactivity when using only iron­(III) triflimide, requiring elevated temperatures and a reaction time of 23 h, was thioarylated using the dual-catalytic process at room temperature, producing **9l** in 73% yield. Similarly, pyrrole ester **7n**, which demonstrated 50% conversion after 7 days with the iron-only reaction, was complete at room temperature in 1 h using the dual catalytic process. The 36% isolated yield for **9n** is due to the formation of byproducts, including the C2-thioarylated regioisomer.

**Scheme 3:**
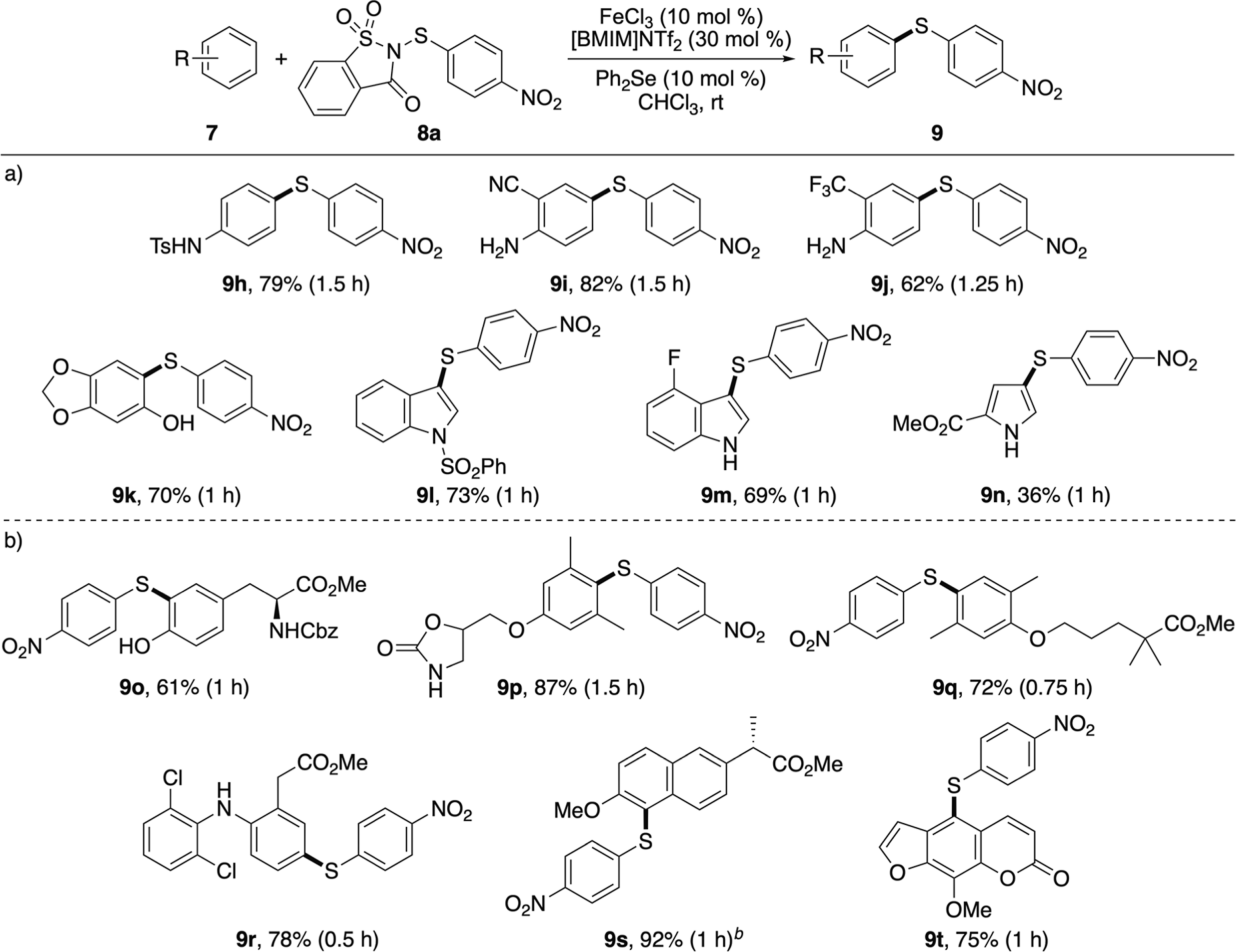
Expansion of the Substrate Scope Using the Dual-Catalytic Process^a^ ^,^ ^b^

a Isolated yields.
b Gram-scale reaction.

To assess functional-group tolerance and selectivity, the dual-catalytic protocol was next applied to a series of structurally complex arenes, including natural product derivatives and pharmaceutical agents (Scheme 3b). Tyrosine derivative (**7o**) underwent rapid thioarylation at room temperature to afford **9o** in 61% yield after 1 h, while the muscle relaxant metaxalone was converted to **9p** in 87% yield under analogous conditions. Methyl ester derivatives of gemfibrozil (**7q**) and the nonsteroidal anti-inflammatory drugs diclofenac (**7r**) and naproxen (**7s**) all reacted smoothly, delivering the corresponding 4-nitrophenylsulfanes in high yields (72–92%). To provide material for subsequent studies (vide infra), the thioarylation of naproxen methyl ester **7s** was conducted on a gram scale, which gave **9s** in a similar yield (92%). The highly activated arene core of the natural product and the psoriasis drug methoxsalen (**7t**) also underwent clean thioarylation to furnish **9t** in 75% yield. Collectively, these results demonstrate that the rapid thioarylation enabled by the dual-catalytic system proceeds under sufficiently mild conditions to be fully compatible with complex, pharmaceutically relevant substrates while exhibiting high selectivity for the most electronically activated arene position.

Having explored the substrate scope of the dual-catalyzed thioarylation for the incorporation of electron-deficient 4-nitrothioaryl groups, we next evaluated whether the enhanced reactivity of this transformation could be used for relatively deactivated substrates in the synthesis of biologically relevant targets. We previously investigated the preparation of a small library of analogues of the HIV-1 reverse transcriptase inhibitor L-737,126 via iron­(III) triflimide-catalyzed thioarylation of indole derivatives with *N*-(thioaryl)­saccharin reagents. However, reactions of indole-2-carboxylic acid (**10**) or the corresponding amide required elevated temperatures (40 °C) and extended reaction times (up to 42 h), which resulted in poor conversions, product mixtures, and low yields (0–28%). Thus, the thioarylation of indole-2-carboxylic acid **10** was examined using the Lewis acid/Lewis base dual-catalyzed protocol (Scheme 4a). For more reactive *N*-(thioaryl)­saccharin reagents, the use of 2.5 mol % of both iron­(III) triflimide and diphenyl selenide afforded clean reactions with short reaction times (3.5–7 h), delivering thioarylated products **11b**–**11d** in 62–77% yield. For comparison, the thioarylation step during the original synthesis of L-737,126 employed sodium hydride and diphenyl disulfide at 50 °C for 24 h, which gave **11b** in 60% yield.^10a^ The less reactive *N*-(4-nitrophenylthio)­saccharin (**8a**) required the standard catalyst loadings (10 mol % of each catalyst), providing **11a** in 45% yield after 3 h. These results highlight the advantages of the dual-catalyzed thioarylation, which enables functionalization of challenging substrates such as **10** under milder conditions and with reduced reaction times. Completion of the three-step synthesis of L-737,126 (**6**) was achieved by conversion of indole-2-carboxylic acid **11b** to amide **12** using thionyl chloride and ammonia (76% yield), followed by oxidation of the sulfane with *m*-chloroperbenzoic acid (*m*CPBA). At room temperature, this gave L-737,126 in 63% yield.

**Scheme 4:**
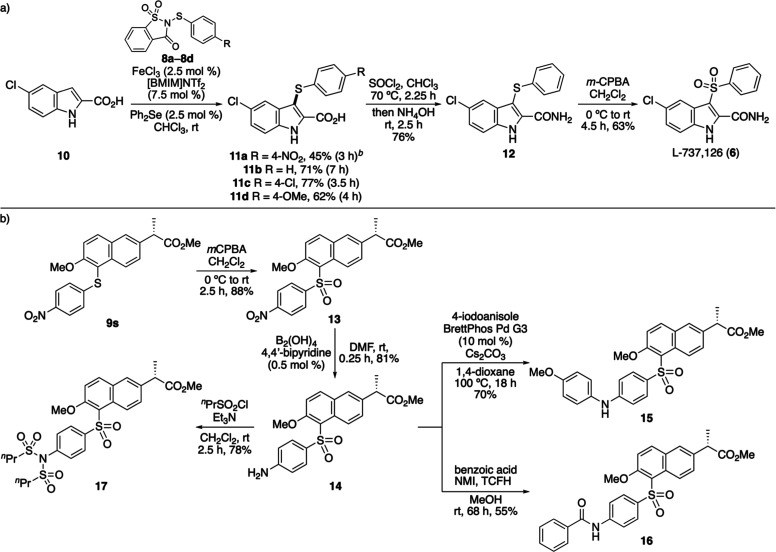
(a) Three-Step Synthesis of the HIV-1 Reverse Transcriptase Inhibitor L-737,126; (b) Late-Stage Functionalization of the Naproxen Analogue **9s** ^a^ ^,^ ^b^

a Isolated yields.
b The synthesis of **11a** was done using Fe­(NTf_2_)_3_ (10 mol %) and Ph_2_Se (10 mol %).

The primary motivation for developing an accelerated, mild thioarylation of 4-nitrophenylsulfanes was to enable access to biaryl sulfides amenable to downstream functionalization via nitro-group transformations for medicinal chemistry applications. To demonstrate this potential, the naproxen analogue **9s** was selected as a representative substrate for further derivatization (Scheme 4b). Oxidation of sulfane **9s** with *m*CPBA proceeded smoothly to afford sulfone **13** in 88% yield. Subsequent reduction of the nitro group to provide a functional handle for late-stage diversification was achieved using tetrahydroxydiboron as the reductant and catalytic 4,4′-bipyridine, following the recently reported protocol by Han and co-workers.^21^ This mild, room-temperature procedure gave aniline **14** in 81% yield after 0.25 h. The synthetic utility of intermediate **14** was further demonstrated through standard medicinal chemistry transformations. For example, Buchwald–Hartwig amination^22^ with 4-iodoanisole, catalyzed by BrettPhos Pd G3, afforded biaryl amine **15** in 70% yield.^23^ Amidation of **14** with benzoic acid, using *N*,*N*,*N′*,*N′*-tetramethylchloroformamidinium hexafluorophosphate (TCFH) and *N*-methylimidazole (NMI) to generate the acyl imidazolium intermediate, delivered amide **16** in 55% yield, while sulfonylation using ^*n*^propylsulfonyl chloride under standard conditions gave bis-sulfonamide **17** in 78% yield.^24^ These results illustrate that 4-nitrobiaryl sulfides, readily accessed under mild conditions via the dual-catalytic thioarylation process, provide versatile intermediates for the late-stage diversification of pharmaceutically relevant scaffolds.

## Conclusions

In summary, a dual catalytic approach combining iron catalysis with diphenyl selenide as a Lewis base cocatalyst has been developed to enable efficient thioarylation under mild conditions. The cooperative effect of this catalytic system substantially enhances the reactivity of electron-deficient saccharin-based reagents, allowing reactions to proceed at room temperature with a broadened substrate scope. The method exhibits functional group tolerance and is applicable to late-stage functionalization of structurally complex molecules. Its synthetic utility is demonstrated by a concise synthesis of the HIV-1 reverse transcriptase inhibitor L-737,126 and the direct structural modification of the pharmaceutical agent naproxen. Future efforts will focus on expanding the scope and applications of this methodology to facilitate the discovery and diversification of bioactive compounds relevant to medicinal chemistry.

## Experimental Section

All reagents and starting materials were obtained from commercial sources and used as received. *N*-(Arylthio)­saccharin reagents **8c** and **8d** were prepared as previously described.^17^ A modified procedure for the preparation of **8a** is described below. All reactions performed at elevated temperatures were heated using an oil bath or a heating mantle. Brine refers to a saturated aqueous solution of sodium chloride. Flash column chromatography was performed using silica gel 60 (40–63 μm) or disposable normal phase silica cartridges using a Teledyne ISCO Combiflash Rf^+^ apparatus and the solvent gradient specified. Purification by reverse phase chromatography was carried out on a Teledyne ISCO ACCQ Prep HP150 with a Waters Xselect CSH prep C18 5 μm 30 × 100 mm column using the solvent gradient specified. Aluminum-backed plates precoated with silica gel 60F_254_ were used for thin layer chromatography and were visualized with a UV lamp or by staining with potassium permanganate. ^1^H NMR spectra were recorded on a NMR spectrometer at either 400 or 500 MHz, and data are reported as follows: chemical shift in ppm relative to the solvent as the internal standard (CHCl_3_, δ 7.26 ppm; DMSO, δ 2.50), multiplicity (s = singlet, d = doublet, t = triplet, q = quartet, m = multiplet or overlap of nonequivalent resonances, integration). Abbreviation br s refers to broad singlet. ^13^C NMR spectra were recorded on a NMR spectrometer at either 101 or 126 MHz, and data are reported as follows: chemical shift in ppm relative to tetramethylsilane or the solvent as the internal standard (CDCl_3_, δ 77.2 ppm; DMSO-*d*_6,_ δ 39.5), multiplicity with respect to hydrogen (deduced from DEPT experiments, C, CH, CH_2_ or CH_3_). Infrared spectra were recorded on a FTIR spectrometer; wavenumbers are indicated in cm^–1^. Mass spectra were recorded using electron impact (EI) or electrospray (ESI) techniques on a quadrupole time-of-flight (Q-TOF) mass spectrometer. Melting points are uncorrected. Optical rotations were determined as solutions irradiated with the sodium D line (λ = 589 nm) using a polarimeter. [α]_D_ values are given in units 10^–1^ deg cm^–1^ g^–1^.

### *N*-(4-Nitrophenylthio)­saccharin (**8a**)^17^

4-Nitrothiophenol (0.714 g, 4.60 mmol) in dry dichloromethane (6 mL) was added dropwise to a stirred solution of *N*-chlorosaccharin (1.00 g, 4.60 mmol) in dry dichloromethane (9 mL) at 0 °C under argon. A solution of triethylamine (0.64 mL, 4.60 mmol) in dry dichloromethane (4 mL) was then added dropwise. The reaction mixture was stirred at 0 °C for 10 min. The reaction mixture was then diluted with dichloromethane (25 mL) and washed with water (25 mL). The aqueous layer was extracted with dichloromethane (2 × 35 mL), and the combined organic layers were washed with brine (60 mL). Purification by recrystallization (dichloromethane/diethyl ether, 1:5) gave *N*-(4-nitrophenylthio)­saccharin (**8a**) (1.23 g, 79%) as a white solid. Spectroscopic data were consistent with the literature.^17^ Mp 164–166 °C; ^1^H NMR (400 MHz, CDCl_3_): δ 8.23–8.16 (m, 3H), 8.03 (ddd, *J* = 7.5, 1.5, 0.7 Hz, 1H), 7.99 (td, *J* = 7.5, 1.2 Hz, 1H), 7.92 (td, *J* = 7.5, 1.5 Hz, 1H), 7.70–7.65 (m, 2H); ^13^C­{^1^H} NMR (101 MHz, CDCl_3_): δ 159.3, 147.9, 142.6, 138.3, 136.2, 135.0, 128.1, 126.7, 126.4, 124.6, 122.0; MS (ESI) *m*/*z*: [M + Na]^+^ calcd for C_13_H_8_N_2_O_5_S_2_Na, 358.98; found, 358.98.

### *N*-(Phenylthio)­saccharin (**8b**)^25^

A solution of thiophenol (0.940 mL, 9.20 mmol) in dry dichloromethane (12 mL) was added to a stirred solution of *N*-chlorosaccharin (2.00 g, 9.20 mmol) in dry dichloromethane (18 mL) at 0 °C under argon and stirred for 10 min. A solution of triethylamine (1.28 mL, 9.20 mmol) in dry dichloromethane (6.8 mL) was then added dropwise. The resulting mixture was diluted with dichloromethane (45 mL) and washed with water (2 × 50 mL), and the aqueous layers were combined and extracted with dichloromethane (4 × 35 mL). The combined organic layers were washed with brine (80 mL), dried over MgSO_4_, and then filtered and concentrated in vacuo. Purification by recrystallization (diethyl ether/dichloromethane, 5:1) gave *N*-(phenylthio)­saccharin (**8b**) (1.45 g, 54%) as a white crystalline solid. Mp 141–143 °C (lit.^25^ 140–141 °C); ^1^H NMR (400 MHz, CDCl_3_): δ 8.10 (ddd, *J* = 7.6, 1.2, 0.7 Hz, 1H), 7.95 (ddd, *J* = 7.6, 1.2, 0.7 Hz, 1H), 7.90 (td, *J* = 7.6, 1.2 Hz, 1H), 7.86–7.80 (m, 3H), 7.42–7.33 (m, 3H); ^13^C­{^1^H} NMR (101 MHz, CDCl_3_): δ 159.4, 138.1, 135.5, 134.4, 133.1, 133.0, 130.5, 129.3, 127.1, 125.9, 121.6; MS (ESI) *m*/*z*: [M + NH_4_]^+^ calcd for C_13_H_9_NO_3_S_2_NH_4_, 309.04; found, 309.03.

### General Procedure: Thioarylation of Arenes Using Iron­(III) Triflimide and Diphenyl Selenide

Iron­(III) chloride (10 mol %) was dissolved in 1-butyl-3-methylimidazolium bis­(trifluoromethanesulfonyl)­imide (30 mol %) and left to stir for 0.5 h at room temperature before being added to a solution of *N*-(4-nitrophenylthio)­saccharin (0.3 mmol) and the arene (0.23 mmol) in chloroform (0.5 mL). Diphenyl selenide (10 mol %) was then added. The reaction mixture was left to stir at room temperature for 0.5–1.25 h. The mixture was diluted with dichloromethane (10 mL), filtered through a short pad of Celite, washed with dichloromethane (10 mL), and then concentrated in vacuo. Purification by flash column chromatography gave the desired product.

### (4′-Nitrophenyl)­(3-methyl-4-methoxyphenyl)­sulfane (**9a**)

The reaction was performed as described in the general procedure using 2-methylanisole (28.5 μL, 0.230 mmol) and *N*-(4-nitrophenylthio)­saccharin (**8a**) (0.0997 g, 0.296 mmol). The reaction mixture was stirred at room temperature for 0.5 h. Purification by flash column chromatography (0–40% dichloromethane in hexane) gave (4′-nitrophenyl)­(3-methyl-4-methoxyphenyl)­sulfane (**9a**) (0.0538 g, 85%) as a yellow solid. Mp 123–125 °C; IR (neat) 2917, 1575, 1503, 1334, 1248, 1082, 1029 cm^–1^; ^1^H NMR (400 MHz, CDCl_3_): δ 8.06–8.01 (m, 2H), 7.38 (dd, *J* = 8.4, 2.4 Hz, 1H), 7.32 (d, *J* = 2.4 Hz, 1H), 7.12–7.06 (m, 2H), 6.90 (d, *J* = 8.4 Hz, 1H), 3.89 (s, 3H), 2.24 (s, 3H); ^13^C­{^1^H} NMR (101 MHz, CDCl_3_): δ 159.5, 150.6, 145.1, 137.8, 134.8, 129.1, 125.7, 124.1, 119.5, 111.3, 55.6, 16.3; HRMS (ESI) *m*/*z*: [M]^+^ calcd for C_14_H_13_NO_3_S, 275.0611; found, 275.0610.

### (4-Methoxynaphthalen-1-yl)­(4′-nitrophenyl)­sulfane (**9b**)^26^

The reaction was performed as described in the general procedure using 1-methoxynaphthalene (32.0 μL, 0.220 mmol) and *N*-(4-nitrophenylthio)­saccharin (**8**) (0.0996 g, 0.296 mmol). The reaction mixture was stirred at room temperature for 1 h. Purification by flash column chromatography (50% dichloromethane in hexane) gave (4-methoxynaphthalen-1-yl)­(4′-nitrophenyl)­sulfane (**9b**) (0.0601 g, 88%) as a yellow solid. Spectroscopic data were consistent with the literature.^26^ Mp 161–166 °C; ^1^H NMR (400 MHz, CDCl_3_): δ 8.40–8.35 (m, 1H), 8.19–8.13 (m, 1H), 8.00–7.95 (m, 2H), 7.85 (d, *J* = 8.0 Hz, 1H), 7.58–7.51 (m, 2H), 7.04–6.98 (m, 2H), 6.89 (d, *J* = 8.0 Hz, 1H), 4.09 (s, 3H); ^13^C­{^1^H} NMR (101 MHz, CDCl_3_): δ 158.3, 149.8, 145.1, 137.1, 135.1, 128.4, 126.9, 126.3, 125.5, 124.1, 123.1, 117.0, 104.3, 56.0; MS (ESI) *m*/*z*: [M + H]^+^ calcd for C_17_H_13_NO_3_SH, 312.07; found, 312.07.

### (2,3-Dihydro-1-benzofuran-5-yl)­(4′-nitrophenyl)­sulfane (**9c**)

The reaction was performed as described in the general procedure using 2,3-dihydrobenzofuran (26.0 μL, 0.230 mmol) and *N*-(4-nitrophenylthio)­saccharin (**8a**) (0.100 g, 0.299 mmol). The reaction mixture was stirred at room temperature for 1 h. Purification by flash column chromatography (60% dichloromethane in hexane) gave (2,3-dihydro-1-benzofuran-5-yl)­(4′-nitrophenyl)­sulfane (**9c**) (0.0539 g, 86%) as a yellow solid. Mp 98–100 °C; IR (neat) 3097, 2897, 1575, 1506, 1332, 1230, 1084 cm^–1^; ^1^H NMR (400 MHz, CDCl_3_): δ 8.07–8.01 (m, 2H), 7.37 (br s, 1H), 7.33 (d, *J* = 8.2 Hz, 1H), 7.13–7.07 (m, 2H), 6.87 (d, *J* = 8.2 Hz, 1H), 4.67 (t, *J* = 8.8 Hz, 2H), 3.26 (t, *J* = 8.8 Hz, 2H); ^13^C­{^1^H} NMR (101 MHz, CDCl_3_): δ 162.1, 150.6, 145.1, 136.4, 132.5, 129.7, 125.6, 124.1, 119.7, 111.1, 72.0, 29.6; HRMS (ESI) *m*/*z*: [M]^+^ calcd for C_14_H_11_NO_3_S, 273.0454; found, 273.0446.

### (2-Bromo-4-hydroxyphenyl)­(4′-nitrophenyl)­sulfane (**9d**)

The reaction was performed as described in the general procedure using 3-bromophenol (24.5 μL, 0.231 mmol) and *N*-(4-nitrophenylthio)­saccharin (**8a**) (0.0997 g, 0.296 mmol). The reaction was stirred at room temperature for 1.5 h. Purification by flash chromatography (100% dichloromethane) gave (2-bromo-4-hydroxyphenyl)­(4′-nitrophenyl)­sulfane (**9d**) (0.0492 g, 65%) as a yellow solid. Mp 161–165 °C; IR (neat) 3423, 1568, 1497, 1331, 1283, 1086 cm^–1^; ^1^H NMR (400 MHz, CDCl_3_): δ 8.08 (d, *J* = 8.0 Hz, 2H), 7.57 (d, *J* = 8.4 Hz, 1H), 7.30 (d, *J* = 2.4 Hz, 1H), 7.09 (d, *J* = 8.0 Hz, 2H), 6.89 (dd, *J* = 8.4, 2.4 Hz, 1H), 5.20 (br s, 1H); ^13^C­{^1^H} NMR (101 MHz, CDCl_3_): δ 158.0, 147.9, 145.5, 138.9, 131.8, 126.0, 124.3, 122.2, 121.5, 116.4; HRMS (ESI) *m*/*z*: [M + Na]^+^ calcd for C_12_H_8_^81^BrNO_3_SNa, 349.9280; found, 349.9274.

### Benzyl [4-(4′-Nitrophenylthio)­phenyl]­carbamate (**9e**)

The reaction was performed as described in the general procedure using *N*-(benzyloxycarbonyl)­aniline (0.0520 g, 0.229 mmol) and *N*-(4-nitrophenylthio)­saccharin (**8a**) (0.100 g, 0.299 mmol). The reaction mixture was stirred at room temperature for 1 h. Purification by flash column chromatography (9–20% ethyl acetate in hexane) gave benzyl [4-(4′-nitrophenylthio)­phenyl] carbamate (**9e**) (0.0555 g, 64%) as a light-yellow solid. Mp 132–135 °C; IR (neat) 3281, 1697, 1512, 1335, 1229, 1063, 826 cm^–1^; ^1^H NMR (400 MHz, CDCl_3_): δ 8.05 (d, *J* = 8.0 Hz, 2H), 7.54–7.46 (m, 4H), 7.43–7.32 (m, 5H), 7.12 (d, *J* = 8.0 Hz, 2H), 6.81 (br s, 1H), 5.23 (s, 2H); ^13^C­{^1^H} NMR (101 MHz, CDCl_3_): δ 153.1, 149.4, 145.4, 139.7, 136.5, 135.8, 128.9, 128.7, 128.6, 126.2, 124.2, 123.8, 119.9, 67.6; HRMS (ESI) *m*/*z*: [M – H]^−^ calcd for C_20_H_15_N_2_O_4_S, 379.0758; found, 379.0755.

### Mesityl-(4′-nitrophenyl)­sulfane (**9f**)^27^

The reaction was performed as described in the general procedure using mesitylene (32.0 μL, 0.230 mmol) and *N*-(4-nitrophenylthio)­saccharin (**8a**) (0.0998 g, 0.297 mmol). The reaction mixture was stirred at room temperature for 2 h. Purification by flash column chromatography (20% dichloromethane in hexane) gave mesityl-(4′-nitrophenyl)­sulfane (**9f**) (0.0463 g, 74%) as a light-yellow solid. Mp 83–86 °C (lit.^27^ 86–88 °C); ^1^H NMR (400 MHz, CDCl_3_): δ 8.03 (d, *J* = 9.0 Hz, 2H), 7.06 (s, 2H), 6.98 (d, *J* = 9.0 Hz, 2H), 2.36 (s, 6H), 2.35 (s, 3H); ^13^C­{^1^H} NMR (101 MHz, CDCl_3_): δ 148.9, 144.9, 143.9, 140.7, 129.9, 124.9, 124.8, 124.2, 21.6, 21.3; MS (ESI) *m*/*z*: [M + H]^+^ calcd for C_15_H_15_NO_2_SH, 274.09; found, 274.09.

### 1*H*-Indol-3-yl­(5-nitro)­(4′-nitrophenyl)­sulfane (**9g**)

The reaction was performed as described in the general procedure using 5-nitroindole (0.0373 g, 0.230 mmol) and *N*-(4-nitrophenylthio)­saccharin (**8a**) (0.0999 g, 0.297 mmol). The reaction mixture was stirred at room temperature for 1 h. Purification by flash column chromatography (70–100% dichloromethane in hexane) gave 1*H*-indol-3-yl­(5-nitro)­(4′-nitrophenyl)­sulfane (**9g**) (0.0432 g, 60%) as a yellow solid. Mp 242–244 °C; IR (neat) 3291, 1577, 1505, 1473, 1330, 1081, 736 cm^–1^; ^1^H NMR (400 MHz, DMSO-*d*_6_): δ 8.23 (d, *J* = 2.2 Hz, 1H), 8.18 (br s, 1H), 8.11 (dd, *J* = 9.0, 2.2 Hz, 1H), 8.05 (d, *J* = 8.0 Hz, 2H), 7.73 (d, *J* = 9.0 Hz, 1H), 7.21 (d, *J* = 8.0 Hz, 2H); ^13^C­{^1^H} NMR (101 MHz, DMSO-*d*_6_): δ 148.6, 144.8, 141.9, 140.2, 137.5, 127.9, 125.3, 124.2, 117.9, 114.6, 113.6, 100.0; HRMS (ESI) *m*/*z*: [M + Na]^+^ calcd for C_14_H_9_N_3_O_4_SNa, 338.0206; found, 338.0206.

### *N*-Tosyl-4-(4′-nitrophenylthio)­aniline (**9h**)

The reaction was performed as described in the general procedure using *N*-tosylaniline (0.0565 g, 0.230 mmol) and *N*-(4-nitrophenylthio)­saccharin (**8a**) (0.100 g, 0.299 mmol). The reaction mixture was stirred at room temperature for 1.5 h. Purification by flash column chromatography (15–20% ethyl acetate in hexane) gave *N*-tosyl-4-(4′-nitrophenylthio)­aniline (**9h**) (0.0720 g, 79%) as a light-yellow solid. Mp 152–155 °C; IR (neat) 3245, 1594, 1514, 1493, 1457, 1392, 1335, 1155 cm^–1^; ^1^H NMR (400 MHz, CDCl_3_): δ 8.05–8.02 (m, 2H), 7.78–7.74 (m, 2H), 7.42–7.39 (m, 2H), 7.32–7.28 (m, 3H), 7.19–7.16 (m, 2H), 7.10–7.07 (m, 2H), 2.42 (s, 3H); ^13^C­{^1^H} NMR (101 MHz, CDCl_3_): δ 148.6, 145.5, 144.6, 138.5, 136.3, 136.0, 130.0, 127.5, 126.5, 126.1, 124.2, 121.6, 21.8; HRMS (ESI) *m*/*z*: [M – H]^−^ calcd for C_19_H_15_N_2_O_4_S_2_ 399.0479; found, 399.0478.

### 2-Cyano-4-(4′-nitrophenylthio)­aniline (**9i**)

The reaction was performed as described in the general procedure using 2-cyanoaniline (0.0270 g, 0.230 mmol) and *N*-(4-nitrophenylthio)­saccharin (**8a**) (0.100 g, 0.299 mmol). The reaction mixture was stirred at room temperature for 1.5 h. Purification by flash column chromatography (20–30% ethyl acetate in hexane) gave 2-cyano-4-(4′-nitrophenylthio)­aniline (**9i**) (0.0508 g, 82%) as a yellow solid. Mp 162–165 °C; IR (neat) 3457, 3364, 2211, 1637, 1575, 1333, 1259, 1084 cm^–1^; ^1^H NMR (400 MHz, CDCl_3_): δ 8.09–8.05 (m, 2H), 7.63 (d, *J* = 2.1 Hz, 1H), 7.48 (dd, *J* = 8.7, 2.1 Hz, 1H), 7.13–7.09 (m, 2H), 6.83 (br d, *J* = 8.7 Hz, 1H), 4.73 (br s, 2H); ^13^C­{^1^H} NMR (101 MHz, CDCl_3_): δ 150.8, 148.9, 145.5, 141.4, 139.9, 126.0, 124.3, 117.5, 116.8, 116.4, 97.6; HRMS (ESI) *m*/*z*: [M – H]^−^ calcd for C_13_H_8_N_3_O_2_S, 270.0343; found, 270.0344.

### 2-Trifluoro-4-(4′-nitrophenylthio)­aniline (**9j**)

The reaction was performed as described in the general procedure using 2-(trifluoromethyl)­aniline (0.0372 g, 0.231 mmol) and *N*-(4-nitrophenylthio)­saccharin (**8a**) (0.100 g, 0.299 mmol). The reaction mixture was stirred at room temperature for 1.25 h. Purification by flash column chromatography (15% ethyl acetate in hexane) gave 2-trifluoro-4-(4′-nitrophenylthio)­aniline (**9j**) (0.0448 g, 62%) as a yellow solid. Mp 88–93 °C; IR (neat) 3467, 3388, 3087, 2916, 1631, 1574, 1491, 1330, 1069, 835 cm^–1^; ^1^H NMR (400 MHz, CDCl_3_): δ 8.08–8.04 (m, 2H), 7.65 (d, *J* = 2.1 Hz, 1H), 7.45 (dd, *J* = 8.5, 2.1 Hz, 1H), 7.12–7.08 (m, 2H), 6.82 (br d, *J* = 8.5 Hz, 1H), 4.50 (br s, 2H); ^13^C­{^1^H} NMR (101 MHz, CDCl_3_): δ 149.7, 146.3 (q, ^4^ *J*_CF_ = 1.6 Hz), 145.3, 140.4, 134.5 (q, ^3^ *J*_CF_ = 5.2 Hz), 125.7, 124.2, 123.0 (q, ^1^ *J*_CF_ = 273.0 Hz), 118.7, 116.5, 114.9 (q, ^2^ *J*_CF_ = 30.8 Hz); HRMS (APCI) *m*/*z*: [M + Cl]^−^ calcd for C_13_H_9_F_3_N_2_O_2_S^35^Cl, 349.0031; found, 349.0028.

### 3,4-Methylenedioxy-6-(4′-nitrophenylthio)­phenol (**9k**)

The reaction was performed as described in the general procedure using sesamol (0.0317 g, 0.230 mmol) and *N*-(4-nitrophenylthio)­saccharin (**8a**) (0.100 g, 0.299 mmol). The reaction mixture was stirred at room temperature for 1 h. Purification by flash column chromatography (15% ethyl acetate in hexane) gave 3,4-methylenedioxy-6-(4′-nitrophenylthio)­phenol (**9k**) (0.0466 g, 70%) as an orange/yellow solid. Mp 177–181 °C; IR (neat) 3412, 3099, 2923, 1575, 1501, 1469, 1332, 1179, 926, 849 cm^–1^; ^1^H NMR (400 MHz, CDCl_3_): δ 8.11–8.07 (m, 2H), 7.15–7.11 (m, 2H), 6.90 (s, 1H), 6.67 (s, 1H), 6.14 (s, 1H), 6.02 (s, 2H); ^13^C­{^1^H} NMR (101 MHz, CDCl_3_): δ 154.1, 152.1, 146.5, 146.0, 142.5, 125.8, 124.4, 114.3, 102.8, 102.1, 98.1; HRMS (ESI) *m*/*z*: [M – H]^−^ calcd for C_13_H_8_NO_5_S, 290.0129; found, 290.0131.

### [1-(Phenylsulfonyl)­indol-3-yl]-(4′-nitrophenyl)­sulfane (**9l**)

The reaction was performed as described in the general procedure using 1-(phenylsulfonyl)­indole (0.0589 g, 0.230 mmol) and *N*-(4-nitrophenylthio)­saccharin (**8a**) (0.100 g, 0.299 mmol). The reaction mixture was stirred at room temperature for 1 h. Purification by flash column chromatography (50% dichloromethane in hexane) gave [1-(phenylsulfonyl)­indol-3-yl]-(4′-nitrophenyl)­sulfane (**9l**) (0.0686 g, 73%) as a light-yellow solid. Mp 133–136 °C; IR (neat) 3128, 3058, 1577, 1509, 1475, 1445, 1368, 1335, 1172, 1086, 729 cm^–1^; ^1^H NMR (400 MHz, CDCl_3_) δ 8.07 (dt, *J* = 8.3, 0.9 Hz, 1H), 7.95–8.02 (m, 4H), 7.94 (s, 1H), 7.65–7.61 (m, 1H), 7.54–7.50 (m, 2H), 7.44–7.40 (m, 1H), 7.40–7.37 (m, 1H), 7.28–7.24 (m, 1H), 7.10–7.06 (m, 2H); ^13^C­{^1^H} NMR (101 MHz, CDCl_3_): δ 146.8, 145.6, 137.9, 135.6, 134.6, 132.3, 130.5, 129.8, 127.1, 126.2, 125.9, 124.5, 124.2, 120.2, 114.2, 108.8; HRMS (ESI) *m*/*z*: [M – H]^−^ calcd for C_20_H_13_N_2_O_4_S_2_, 409.0322; found, 409.0323.

### 1*H*-Indol-3-yl-(4-fluoro)­(4′-nitrophenyl)­sulfane (**9m**)

The reaction was performed as described in the general procedure using 4-fluoroindole (0.0312 g, 0.230 mmol) and *N*-(4-nitrophenylthio)­saccharin (**8a**) (0.100 g, 0.299 mmol). The reaction mixture was stirred at room temperature for 1 h. Purification by flash column chromatography (60–80% dichloromethane in hexane) gave 1*H*-indol-3-yl-(4-fluoro)­(4′-nitrophenyl)­sulfane (**9m**) (0.0461 g, 69%) as a yellow solid. Mp 182–185 °C; IR (neat) 3437, 3110, 1631, 1573, 1493, 1404, 1322, 1083, 838 cm^–1^; ^1^H NMR (400 MHz, CDCl_3_): δ 8.65 (br s, 1H), 8.05–8.02 (m, 2H), 7.49 (d, *J* = 2.7 Hz, 1H), 7.27 (dd, *J* = 8.2, 1.0 Hz, 1H), 7.24–7.16 (m, 3H), 6.82 (ddd, *J* = 10.8, 7.8, 1.0 Hz, 1H); ^13^C­{^1^H} NMR (101 MHz, CDCl_3_): δ 156.8 (d, ^1^ *J*_CF_ = 250.3 Hz), 150.4, 145.1, 139.5 (d, ^3^ *J*_CF_ = 9.8 Hz), 131.7, 125.3, 124.4 (d, ^3^ *J*_CF_ = 7.8 Hz), 124.0, 117.4 (d, ^2^ *J*_CF_ = 18.3 Hz), 108.2 (d, ^4^ *J*_CF_ = 4.3 Hz), 107.0 (d, ^2^ *J*_CF_ = 19.2 Hz), 98.7; HRMS (ESI) *m*/*z*: [M – H]^−^ calcd for C_14_H_8_FN_2_O_2_S, 287.0296; found, 287.0294.

### Methyl 4-(4′-Nitrophenylthio)-1*H*-pyrrole-2-carboxylate (**9n**)

The reaction was performed as described in the general procedure using 1*H*-pyrrole-2-carboxylate (0.0287 g, 0.230 mmol) and *N*-(4-nitrophenylthio)­saccharin (**8a**) (0.100 g, 0.299 mmol). The reaction mixture was stirred at room temperature for 1 h. Purification by flash column chromatography (0–40% ethyl acetate in hexane) gave methyl 4-(4′-nitrophenylthio)-1*H*-pyrrole-2-carboxylate (**9n**) (0.0227 g, 36%) as a white solid. Mp 154–156 °C; IR (neat) 3260, 1688, 1551, 1502, 1443, 1373, 1246, 1206, 1089, 839 cm^–1^; ^1^H NMR (400 MHz, CDCl_3_): δ 9.68 (br s, 1H), 8.07–8.03 (m, 2H), 7.21 (dd, *J* = 2.8, 1.5 Hz, 1H), 7.17–7.13 (m, 2H), 7.02 (dd, *J* = 2.8, 1.5 Hz, 1H), 3.90 (s, 3H); ^13^C­{^1^H} NMR (101 MHz, CDCl_3_): δ 161.1, 149.9, 145.2, 129.1, 125.2, 124.9, 124.1, 120.9, 109.2, 52.1; HRMS (APCI) *m*/*z*: [M – H]^−^ calcd for C_12_H_9_N_2_O_4_S, 277.0289; found, 277.0296.

### *N*-(Benzyloxycarbonyl)-3′-(4″-nitrophenylthio)-l-tyrosine Methyl Ester (**9o**)

The reaction was performed as described in the general procedure using *N*-(benzyloxycarbonyl)-l-tyrosine methyl ester (0.0753 g, 0.229 mmol) and *N*-(4-nitrophenylthio)­saccharin (**8a**) (0.100 g, 0.299 mmol). The reaction mixture was stirred at room temperature for 1 h. Purification by flash column chromatography (dichloromethane/diethyl ether, 100:2) gave *N*-(benzyloxycarbonyl)-3′-(4″-nitrophenylthio)-l-tyrosine methyl ester (**9o**) (0.0671 g, 61%) as a yellow crystalline solid. Mp 106–108 °C; [α]_D_^22^ +67.9 (*c* 0.1, CHCl_3_); IR (neat); 3344, 2952, 1697, 1577, 1505, 1333, 1211, 1173, 1057 cm^–1^; ^1^H NMR (400 MHz, CDCl_3_): δ 8.04–8.01 (m, 2H), 7.35–7.27 (m, 6H), 7.17 (dd, *J* = 8.4, 2.2 Hz, 1H), 7.07–7.04 (m, 2H), 7.00 (d, *J* = 8.4 Hz, 1H), 6.42–6.37 (m, 1H), 5.36 (br d, *J* = 8.0 Hz, 1H), 5.10 (d, *J* = 12.0 Hz, 1H), 5.05 (d, *J* = 12.0 Hz, 1H), 4.65–4.60 (m, 1H), 3.69 (s, 3H), 3.11 (dd, *J* = 14.0, 5.5 Hz, 1H), 2.98 (dd, *J* = 14.0, 6.3 Hz, 1H); ^13^C­{^1^H} NMR (101 MHz, CDCl_3_): δ 171.8, 156.6, 155.7, 146.0, 145.8, 137.7, 136.2, 134.4, 129.5, 128.6, 128.4, 128.1, 126.0, 124.3, 116.5, 113.8, 67.2, 55.0, 52.6, 37.4; HRMS (ESI) *m*/*z*: [M + Na]^+^ calcd for C_24_H_22_N_2_O_7_SNa, 505.1040; found, 505.1032.

### 5-{[4′-(4-Nitrophenylthio)-3′,5′-dimethylphenoxy]­methyl}­oxazolidine-2″-one (**9p**)

The reaction was performed as described in the general procedure using metaxalone (0.0503 g, 0.227 mmol) and *N*-(4-nitrophenylthio)­saccharin (**8a**) (0.100 g, 0.299 mmol). The reaction mixture was stirred at room temperature for 1.5 h. Purification by flash column chromatography (0–1% methanol in dichloromethane) gave 5-{[4′-(4-nitrophenylthio)-3′,5′-dimethylphenoxy]­methyl}­oxazolidine-2″-one (**9p**) (0.0741 g, 87%) as a light-yellow solid. Mp 105–108 °C; IR (neat) 3251, 2918, 1758, 1733, 1504, 1338, 1312, 1171 cm^–1^; ^1^H NMR (400 MHz, CDCl_3_): δ 8.04–8.02 (m, 2H), 6.97–6.95 (m, 2H), 6.80 (s, 2H), 5.57 (br s, 1H), 5.04–4.98 (m, 1H), 4.23–4.17 (m, 2H), 3.84–3.80 (m, 1H), 3.67–3.63 (m, 1H), 2.37 (s, 6H); ^13^C­{^1^H} NMR (101 MHz, CDCl_3_): δ 159.4, 159.1, 148.9, 146.0, 145.0, 124.8, 124.3, 120.4, 115.1, 74.1, 68.0, 42.7, 22.0; HRMS (ESI) *m*/*z*: [M + H]^+^ calcd for C_18_H_18_N_2_O_5_SH, 375.1009; found, 375.1002.

### 4-(4′-Nitrophenylthio)­gemfibrozil Methyl Ester (**9q**)

The reaction was performed as described in the general procedure using gemfibrozil methyl ester (0.0608 g, 0.230 mmol) and *N*-(4-nitrophenylthio)­saccharin (**8a**) (0.100 g, 0.299 mmol). The reaction mixture was stirred at room temperature for 0.75 h. Purification by flash column chromatography (0–13% ethyl acetate in hexane) gave 4-(4′-nitrophenylthio)-gemfibrozil methyl ester (**9q**) (0.0690 g, 72%) as a yellow oil. IR (neat) 2950, 1727, 1577, 1511, 1473, 1334, 1246, 1144 cm^–1^; ^1^H NMR (400 MHz, CDCl_3_): δ 8.03–8.00 (m, 2H), 7.31 (s, 1H), 7.02–6.98 (m, 2H), 6.78 (s, 1H), 3.98 (2H, t, *J* = 5.6 Hz, 2H), 3.68 (s, 3H), 2.31 (s, 3H), 2.19 (s, 3H), 1.82–1.71 (m, 4H), 1.24 (s, 6H); ^13^C­{^1^H} NMR (101 MHz, CDCl_3_): δ 178.3, 159.0, 150.0, 144.9, 142.1, 138.9, 126.3, 125.1, 124.1, 118.2, 113.5, 68.3, 51.9, 42.2, 37.2, 25.3, 25.2, 20.9, 15.7; HRMS (ESI) *m*/*z*: [M + Na]^+^ calcd for C_22_H_27_NO_5_SNa, 440.1502; found, 440.1496.

### 3-(4″-Nitrophenylthio)­diclofenac Methyl Ester (**9r**)

The reaction was performed as described in the general procedure using diclofenac methyl ester (0.0709 g, 0.229 mmol) and *N*-(4-nitrophenylthio)­saccharin (**8a**) (0.100 g, 0.299 mmol). The reaction mixture was stirred at room temperature for 0.5 h. Purification by flash column chromatography (40–50% dichloromethane in hexane) gave 3-(4″-nitrophenylthio)­diclofenac methyl ester (**9r**) (0.0822 g, 78%) as a light-yellow solid. Mp 109–111 °C; IR (neat) 3370, 3326, 2954, 1723, 1574, 1505 1449, 1338 cm^–1^; ^1^H NMR (400 MHz, CDCl_3_): δ 8.07–8.04 (m, 2H), 7.43–7.42 (m, 1H), 7.39 (br d, *J* = 8.1 Hz, 2H), 7.30 (d, *J* = 8.4 Hz, 1H), 7.22 (br s, 1H), 7.14–7.12 (m, 2H), 7.07 (t, *J* = 8.1 Hz, 1H), 6.57 (d, *J* = 8.4 Hz, 1H), 3.81 (s, 2H), 3.78 (s, 3H); ^13^C­{^1^H} NMR (101 MHz, CDCl_3_): δ 172.4, 149.9, 145.2, 144.8, 138.1, 136.7, 135.6, 130.4, 129.2, 125.9, 125.4, 125.1, 124.1, 120.8, 118.8, 52.9, 38.5; HRMS (ESI) *m*/*z*: [M + H]^+^ calcd for C_21_H_16_^35^Cl_2_N_2_O_4_SH, 463.0281; found, 463.0283.

### 5-(4′-Nitrophenylthio)-(*S*)-naproxen Methyl Ester (**9s**)

The reaction was performed as described in the general procedure using (*S*)-naproxen methyl ester (1.09 g, 4.45 mmol) and *N*-(4-nitrophenylthio)­saccharin (**8a**) (1.95 g, 5.79 mmol). The reaction mixture was stirred at room temperature for 1 h. Purification by flash column chromatography (ethyl acetate/hexane, 3:1) gave 5-(4′-nitrophenylthio)-(*S*)-naproxen methyl ester (**9s**) (1.63 g, 92%) as a yellow crystalline solid. Mp 98–101 °C; [α]_D_^22^ +69.5 (*c* 0.1, CHCl_3_); IR (neat) 2948, 1721, 1591, 1500, 1475, 1330, 1269, 1059 cm^–1^; ^1^H NMR (400 MHz, CDCl_3_): δ 8.26 (br d, *J* = 8.8 Hz, 1H), 8.02 (br d, *J* = 9.1 Hz, 1H), 7.98–7.94 (m, 2H), 7.77 (d, *J* = 1.9 Hz, 1H), 7.48 (dd, *J* = 8.8, 1.9 Hz, 1H), 7.39 (br d, *J* = 9.1 Hz, 1H), 7.04–7.00 (m, 2H), 3.95 (s, 3H), 3.88 (q, *J* = 7.2 Hz, 1H), 3.67 (s, 1H), 1.58 (br d, *J* = 7.2 Hz, 3H); ^13^C­{^1^H} NMR (101 MHz, CDCl_3_): δ 174.8, 159.4, 148.4, 144.9, 136.7, 135.1, 133.1, 129.6, 128.3, 126.7, 125.4, 125.2, 123.9, 113.5, 109.9, 56.9, 52.1, 45.1, 18.5; HRMS (ESI) *m*/*z*: [M + Na]^+^ calcd for C_21_H_19_NO_5_SNa, 420.0876; found, 420.0892.

### 5-(4′-Nitrophenylthio)-8-methoxyfuro­[3′,2′-g]­chromen-2-one (**9t**)

The reaction was performed as described in the general procedure using methoxsalen (0.0494 g, 0.229 mmol) and *N*-(4-nitrophenylthio)­saccharin (**8a**) (0.100 g, 0.299 mmol). The reaction mixture was stirred at room temperature for 1 h. Purification by flash column chromatography (100% dichloromethane) gave 5-(4′-nitrophenylthio)-8-methoxyfuro­[3′,2′-g]­chromen-2-one (**9t**) (0.0634 g, 75%) as a yellow solid. Mp 251–253 °C; IR (neat) 3099, 2951, 1718, 1576, 1508, 1331, 1286, 1109 cm^–1^; ^1^H NMR (400 MHz, CDCl_3_): δ 8.26 (br d, *J* = 9.9 Hz, 1H), 8.07–8.03 (m, 2H), 7.74 (d, *J* = 2.2 Hz, 1H), 7.04–7.00 (m, 2H), 6.85 (d, *J* = 2.2 Hz, 1H), 6.45 (br d, *J* = 9.9 Hz, 1H), 4.43 (s, 3H); ^13^C­{^1^H} NMR (101 MHz, CDCl_3_): δ 159.7, 148.0, 146.7, 146.3, 145.8, 143.8, 141.4, 135.2, 132.5, 125.9, 124.5, 120.0, 116.8, 110.1, 106.9, 61.5; HRMS (ESI) *m*/*z*: [M + H]^+^ calcd for C_18_H_11_NO_6_SH, 370.0380; found, 370.0392.

### 5-Chloro-3-(4′-nitrophenylthio)-1*H*-indole-2-carboxylic Acid (**11a**)

The reaction was performed as described in the general procedure using 5-chloro-1*H*-indole-2-carboxylic acid (**10**) (0.0585 g, 0.299 mmol) and *N*-(4-nitrophenylthio)­saccharin (**8a**) (0.131 g, 0.390 mmol). The reaction mixture was stirred at room temperature for 3 h. Purification by flash column chromatography (5% methanol in diethyl ether) gave 5-chloro-3-(4′-nitrophenylthio)-1*H*-indole-2-carboxylic acid (**11a**) (0.0469 g, 45%) as a yellow solid. Mp 238 °C (decomposition); IR (neat) 3337, 2575, 1670, 1523, 1497, 1330, 1267, 1241 cm^–1^; ^1^H NMR (400 MHz, DMSO-*d*_6_): δ 12.75 (br s, 1H), 8.08–8.04 (m, 2H), 7.59 (d, *J* = 8.8 Hz, 1H), 7.44 (d, *J* = 2.1 Hz, 1H), 7.37 (dd, *J* = 8.8, 2.1 Hz, 1H), 7.22–7.18 (m, 2H); ^13^C­{^1^H} NMR (101 MHz, DMSO-*d*_6_): δ 161.5, 148.6, 144.6, 134.6, 130.3, 126.3, 125.54, 125.45, 124.1, 118.6, 115.3, 114.2, 102.1; HRMS (ESI) *m*/*z*: [M – H]^−^ calcd for C_15_H_8_^35^ClN_2_O_4_S, 346.9899; found, 346.9902.

### 5-Chloro-3-phenylthio-1*H*-indole-2-carboxylic Acid (**11b**)

The reaction was performed as described in the general procedure using 5-chloro-1*H*-indole-2-carboxylic acid (**10**) (0.0588 g, 0.301 mmol) and *N*-(phenylthio)­saccharin (**8b**) (0.114 g, 0.391 mmol), except that iron­(III) chloride (2.5 mol %), 1-butyl-3-methylimidazolium bis­(trifluoromethanesulfonyl)­imide (7.5 mol %), and diphenyl selenide (2.5 mol %) were used. The reaction mixture was stirred at room temperature for 7 h. Purification by reverse phase chromatography (50–99% acetonitrile/0.1% formic acid in water/0.1% formic acid) gave 5-chloro-3-phenylthio-1*H*-indole-2-carboxylic acid (**11b**) (0.0651 g, 71%) as an off-white solid. Mp 197–199 °C; IR (neat) 3402, 1682, 1654, 1523, 1437, 1327, 1243, 792 cm^–1^; ^1^H NMR (400 MHz, DMSO-*d*_6_): δ 13.49 (br s, 1H), 12.48 (s, 1H), 7.53 (d, *J* = 8.6 Hz, 1H), 7.34 (d, *J* = 2.1 Hz, 1H), 7.31 (dd, *J* = 8.6, 2.1 Hz, 1H), 7.26–7.20 (m, 2H), 7.15–7.04 (m, 3H); ^13^C­{^1^H} NMR (101 MHz, DMSO-*d*_6_): δ 161.5, 137.5, 134.5, 132.0, 130.2, 129.0, 126.5, 125.6, 125.3, 119.0, 115.0, 106.0; HRMS (ESI) *m*/*z*: [M – H]^−^ calcd for C_15_H_9_^35^ClNO_2_S, 302.0048; found, 302.0034.

### 5-Chloro-3-(4′-chlorophenylthio)-1*H*-indole-2-carboxylic Acid (**11c**)

The reaction was performed as described in the general procedure using 5-chloro-1*H*-indole-2-carboxylic acid (**10**) (0.0588 g, 0.301 mmol) and *N*-(4-chlorophenylthio)­saccharin (**8c**) (0.127 g, 0.390 mmol), except that iron­(III) chloride (2.5 mol %), 1-butyl-3-methylimidazolium bis­(trifluoromethanesulfonyl)­imide (7.5 mol %), and diphenyl selenide (2.5 mol %) were used. The reaction mixture was stirred at room temperature for 3.5 h. Purification by flash column chromatography (10–13% methanol in dichloromethane) gave 5-chloro-3-(4′-chlorophenylthio)-1*H*-indole-2-carboxylic acid (**11c**) (0.0779 g, 77%) as a light-yellow solid. Mp 192–194 °C (decomposition); IR (neat) 3363, 2506, 1660, 1515, 1474, 1321, 1235, 1090 cm^–1^; ^1^H NMR (400 MHz, DMSO-*d*_6_): δ 12.51 (br s, 1H), 7.54 (d, *J* = 8.8 Hz, 1H), 7.38 (d, *J* = 2.0 Hz, 1H), 7.31 (dd, *J* = 8.8, 2.0 Hz, 1H), 7.27 (d, *J* = 8.8 Hz, 2H), 7.05 (d, *J* = 8.8 Hz, 2H); ^13^C­{^1^H} NMR (101 MHz, DMSO-*d*_6_): δ 162.2, 137.5, 134.9, 133.7, 130.7, 130.2, 129.4, 128.3, 126.2, 125.7, 119.3, 115.5, 105.0; HRMS (ESI) *m*/*z*: [M – H]^−^ calcd for C_15_H_8_^35^Cl_2_NO_2_S, 335.9658; found, 335.9648.

### 5-Chloro-3-(4′-methoxyphenylthio)-1*H*-indole-2-carboxylic Acid (**11d**)

The reaction was performed as described in the general procedure using 5-chloro-1*H*-indole-2-carboxylic acid (**10**) (58.8 mg, 0.301 mmol) and *N*-(4-methoxyphenylthio)­saccharin (**8d**) (125 mg, 0.390 mmol), except that iron­(III) chloride (2.5 mol %), 1-butyl-3-methylimidazolium bis­(trifluoromethanesulfonyl)­imide (7.5 mol %), and diphenyl selenide (2.5 mol %) were used. The reaction mixture was stirred at room temperature for 4 h. Purification by flash column chromatography (10% methanol in dichloromethane) gave 5-chloro-3-(4′-methoxyphenylthio)-1*H*-indole-2-carboxylic acid (**11d**) (62.5 mg, 62%) as a white solid. Mp 215–220 °C; IR (neat) 3337, 1671, 1527, 1494, 1275, 1237, 1179, 1027 cm^–1^; ^1^H NMR (600 MHz, DMSO-*d*_6_): δ 7.49 (d, *J* = 8.7 Hz, 1H), 7.31 (d, *J* = 2.1 Hz, 1H), 7.28 (dd, *J* = 8.7, 2.1 Hz, 1H), 7.19–7.14 (m, 2H), 6.87–6.83 (m, 2H), 3.70 (s, 3H); ^13^C­{^1^H} NMR (151 MHz, DMSO-*d*_6_): δ 161.7, 158.0, 134.5, 130.9, 130.1, 129.9, 127.2, 125.3, 125.2, 119.2, 114.9, 114.8, 108.7, 55.2; HRMS (ESI) *m*/*z*: [M – H]^−^ calcd for C_16_H_11_^35^ClNO_3_S, 332.0154; found, 332.0154.

### 5-Chloro-3-phenylthio-1*H*-indole-2-carboxamide (**12**)^28^

A solution of 5-chloro-3-phenylthio-1*H*-indole-2-carboxylic acid (**11b**) (0.160 g, 0.526 mmol) and thionyl chloride (0.115 mL, 1.58 mmol) in chloroform (1.0 mL) was heated under reflux (70 °C) for 2.25 h. The reaction mixture was cooled to −20 °C. A 25% aqueous solution of ammonium hydroxide (0.9 mL) was then added dropwise, and the reaction mixture was stirred at room temperature for 2.5 h. The precipitate formed was filtered and washed with water to give 5-chloro-3-phenylthio-1*H*-indole-2-carboxamide (**12**) (0.121 g, 76%) as a light-yellow solid. Mp 205–208 °C (lit.^28^ 210–212 °C); ^1^H NMR (600 MHz, CDCl_3_): δ 10.01 (br s, 1H), 8.16 (br s, 1H), 7.63 (d, *J* = 2.1 Hz, 1H), 7.45 (d, *J* = 8.8 Hz, 1H), 7.31 (dd, *J* = 8.8, 2.1 Hz, 1H), 7.26–7.19 (m, 2H), 7.17–7.07 (m, 3H), 5.88 (br s, 1H); ^13^C­{^1^H} NMR (151 MHz, CDCl_3_): δ 162.1, 135.7, 133.9, 133.7, 131.5, 129.5, 128.0, 126.6, 126.5, 126.4, 120.4, 113.8, 103.0; MS (ESI) *m*/*z*: [M + H]^+^ calcd for C_15_H_11_^35^ClN_2_OSH, 303.04; found, 303.04.

### 5-Chloro-3-phenylsulfonyl-1*H*-indole-2-carboxamide (**6**)^10b^

To a stirred solution of 5-chloro-3-phenylthio-1*H*-indole-2-carboxamide (**12**) (0.0995 g, 0.329 mmol) in anhydrous dichloromethane (3.3 mL) was slowly added *meta*-chloroperoxybenzoic acid (0.142 g, 0.822 mmol) in anhydrous dichloromethane (3.3 mL) at 0 °C. The reaction mixture was stirred at room temperature for 4.5 h. A 5% aqueous solution of sodium thiosulfate (5 mL) was added, and the reaction mixture was stirred for 10 min. The mixture was then diluted with dichloromethane (10 mL), and the aqueous layer was extracted with dichloromethane (3 × 20 mL) and ethyl acetate (2 × 20 mL). The organic layers were combined and washed with brine (20 mL), passed through a phase separation filter, and concentrated in vacuo. Purification by normal phase chromatography (0–40% ethyl acetate in cyclohexane) gave 5-chloro-3-phenylsulfonyl-1*H*-indole-2-carboxamide (**6**) (0.0691 g, 63%) as an off-white solid. Mp 255–257 °C (lit.^10b^ 254–255 °C); ^1^H NMR (400 MHz, DMSO-*d*_6_): δ 13.02 (br s, 1H), 8.45 (br s, 1H), 8.21 (br s, 1H), 8.06–8.01 (m, 2H), 7.96 (d, *J* = 2.0 Hz, 1H), 7.68–7.56 (m, 3H), 7.53 (d, *J* = 8.8 Hz, 1H), 7.35 (dd, *J* = 8.8, 2.0 Hz, 1H); ^13^C­{^1^H} NMR (101 MHz, DMSO-*d*_6_): δ 160.6, 142.7, 137.2, 133.3, 132.8, 129.4, 127.3, 126.2, 125.4, 124.8, 118.9, 114.9, 111.1; MS (ESI) *m*/*z*: [M – H]^−^ calcd for C_15_H_10_^35^ClN_2_O_3_S, 333.01; found, 333.01.

### 5-(4′-Nitrophenylsulfonyl)-(*S*)-naproxen Methyl Ester (**13**)

To a stirred solution of 5-(4′-nitrophenylthio)-(*S*)-naproxen methyl ester (**9s**) (0.150 g, 0.378 mmol) in dichloromethane (7.6 mL) was slowly added *meta*-chloroperoxybenzoic acid (0.212 g, 1.23 mmol) at 0 °C. The mixture was stirred at room temperature for 2.5 h. The reaction mixture was diluted with dichloromethane (6 mL) and washed with a 7.5% aqueous solution of sodium sulfite (15 mL). The aqueous layer was extracted with dichloromethane (3 × 15 mL), and the combined organic layers were washed with brine (15 mL). The organic phase was dried (MgSO_4_), filtered, and concentrated in vacuo to give 5-(4′-nitrophenylsulfonyl)-(*S*)-naproxen methyl ester (**13**) (0.144 g, 88%) as a yellow crystalline solid. Mp 104–106 °C; [α]_D_^18^ +28.6 (*c* 0.1, CHCl_3_); IR (neat) 3103, 2949, 1730, 1598, 1526, 1501, 1346, 1281 cm^–1^; ^1^H NMR (400 MHz, CDCl_3_): δ 9.42 (d, *J* = 9.2 Hz, 1H), 8.32–8.27 (m, 2H), 8.15–8.09 (m, 2H), 8.05 (d, *J* = 9.1 Hz, 1H), 7.74 (d, *J* = 2.1 Hz, 1H), 7.65 (dd, *J* = 9.2, 2.1 Hz, 1H), 7.18 (d, *J* = 9.1 Hz, 1H), 3.90 (q, *J* = 7.2 Hz, 1H), 3.77 (s, 3H), 3.69 (s, 1H), 1.61 (br d, *J* = 7.2 Hz, 3H); ^13^C­{^1^H} NMR (101 MHz, CDCl_3_): δ 174.7, 158.0, 150.0, 149.9, 137.8, 137.3, 130.6, 129.7, 129.5, 128.5, 127.1, 124.1, 123.8, 120.2, 113.6, 56.9, 52.4, 45.1, 18.5; HRMS (ESI) *m*/*z*: [M + H]^+^ calcd for C_21_H_19_NO_7_SH, 430.0955; found, 430.0958.

### 5-(4′-Aminophenylsulfonyl)-(*S*)-naproxen Methyl Ester (**14**)

To a stirred solution of 5-(4′-nitrophenylsulfonyl)-(*S*)-naproxen methyl ester (**13**) (43.0 mg, 1.00 mmol) and tetrahydroxydiboron (26.9 mg, 0.30 mmol) in anhydrous DMF (1 mL) was added 4,4′-bipyridine (0.0781 mg, 0.000500 mmol, 0.5 mol %) dropwise at 0 °C over 5 min. The mixture was left to stir at 25 °C for 0.25 h. The reaction mixture was diluted with water (2 mL) and ethyl acetate (2 mL), and the two layers were separated. The organic layer was washed with brine (1 mL), dried (MgSO_4_), filtered, and concentrated in vacuo to give 5-(4′-aminophenylsulfonyl)-(*S*)-naproxen methyl ester (**14**) (32.3 mg, 81%) as an off-white solid. Mp 202–205 °C; [α]_D_^19^ +38.0 (*c* 0.1, CHCl_3_); IR (neat) 3498, 3390, 1725, 1593, 1498, 1279, 1258, 1130, 1080 cm^–1^; ^1^H NMR (400 MHz, CDCl_3_): δ 9.50 (d, *J* = 9.2 Hz, 1H), 7.95 (d, *J* = 9.1 Hz, 1H), 7.80–7.75 (m, 2H), 7.68 (d, *J* = 2.1 Hz, 1H), 7.59 (dd, *J* = 9.2, 2.1 Hz, 1H), 7.18 (d, *J* = 9.1 Hz, 1H), 6.66–6.60 (m, 2H), 4.05 (br s, 2H), 3.88 (q, *J* = 7.2 Hz, 1H), 3.82 (s, 3H), 3.69 (s, 3H), 1.59 (d, *J* = 7.2 Hz, 3H); ^13^C­{^1^H} NMR (101 MHz, CDCl_3_): δ 174.9, 157.6, 150.6, 136.7, 136.1, 132.8, 130.6, 129.8, 129.6, 128.8, 126.8, 124.9, 123.3, 114.3, 113.5, 57.2, 52.3, 45.1, 18.5; HRMS (ESI) *m*/*z*: [M + H]^+^ calcd for C_21_H_21_NO_5_SH, 400.1213; found, 400.1219.

### 5-(4′-(4″-Methoxyphenyl)­amino)­phenylsulfonyl)-(*S*)-naproxen Methyl Ester (**15**)

5-(4′-Aminophenylsulfonyl)-(*S*)-naproxen methyl ester (**14**) (0.140 g, 0.350 mmol), 4-iodoanisole (0.0818 g, 0.350 mmol), cesium carbonate (0.228 g, 0.701 mmol), and BrettPhos Pd G3 (0.0321 g, 0.0354 mmol, 10 mol %) were added to a sealed microwave vial. The vial was subsequently evacuated and refilled with nitrogen five times, and then, anhydrous 1,4-dioxane (2 mL) was added, and the mixture was degassed for 5 min. The reaction mixture was left to stir at 100 °C for 18 h. The reaction mixture was cooled to room temperature and concentrated under vacuum. The resulting residue was suspended in methanol (30 mL) and filtered through Celite, which was washed with an additional portion of methanol (30 mL). The resulting solution was concentrated in vacuo. Purification by normal phase chromatography (0–50% ethyl acetate/1% triethylamine in cyclohexane) gave 5-(4′-(4″-methoxyphenyl)­amino)­phenylsulfonyl)-(*S*)-naproxen methyl ester (**15**) (0.123 g, 70%) as a brown solid. Mp 65–68 °C; [α]_D_^23^ –16.0 (*c* 0.1, CHCl_3_); IR (neat) 3360, 2940, 1729, 1593, 1511, 1243, 1132, 1086 cm^–1^; ^1^H NMR (400 MHz, DMSO-*d*_6_): δ 9.30 (1H, d, *J* 9.2 Hz, 1H), 8.55 (br s, 1H), 8.20 (d, *J* = 9.2 Hz, 1H), 7.85 (d, *J* = 2.1 Hz, 1H), 7.70–7.65 (m, 2H), 7.59 (dd, *J* = 9.2, 2.1 Hz, 1H), 7.49 (d, *J* = 9.2 Hz, 1H), 7.13–7.07 (m, 2H), 6.94–6.87 (m, 4H), 3.97 (q, *J* = 7.1 Hz, 1H), 3.82 (s, 3H), 3.73 (s, 3H), 3.61 (s, 3H), 1.50 (d, *J* = 7.1 Hz, 3H); ^13^C­{^1^H} NMR (101 MHz, DMSO-*d*_6_): δ 174.1, 157.3, 155.4, 149.7, 136.3, 136.1, 133.5, 130.6, 129.2, 129.2, 128.8, 128.4, 126.9, 123.6, 123.1, 122.1, 115.0, 114.6, 112.2, 56.9, 55.2, 51.9, 43.9, 18.1; HRMS (ESI) *m*/*z*: [M + H]^+^ calcd for C_28_H_27_NO_6_SH, 506.1632; found, 506.1631.

### 5-[(4′-Benzamido)­phenylsulfonyl]-(*S*)-naproxen Methyl Ester (**16**)

To a solution of benzoic acid (0.0134 g, 0.110 mmol), 5-(4′-aminophenylsulfonyl)-(*S*)-naproxen methyl ester (**14**) (0.0529 g, 0.132 mmol) and 1-methylimidazole (27.6 μL, 0.348 mmol) in anhydrous acetonitrile (0.5 mL) was added *N*,*N*,*N′*,*N′*-tetramethylchloroformamidinium hexafluorophosphate (0.0511 g, 0.182 mmol). The reaction mixture was stirred at room temperature for 68 h. Following concentration under vacuum, purification by normal phase chromatography (0–50% ethyl acetate in cyclohexane) gave 5-[(4′-benzamido)­phenylsulfonyl]-(*S*)-naproxen methyl ester (**16**) (0.0305 g, 55%) as a white solid. Mp 177–179 °C, [α]_D_^23^ +12.0 (*c* 0.1, CHCl_3_); IR (neat) 3388, 1729, 1679, 1589, 1523, 1498, 1251, 1136 cm^–1^; ^1^H NMR (400 MHz, CDCl_3_): δ 9.48 (d, *J* = 9.2 Hz, 1H), 8.10 (br s, 1H), 7.98 (d, *J* = 9.1 Hz, 1H), 7.96–7.92 (m, 2H), 7.89–7.84 (m, 2H), 7.79–7.72 (m, 2H), 7.70 (d, *J* = 2.1 Hz, 1H), 7.60 (dd, *J* = 9.2, 2.1 Hz, 1H), 7.58–7.52 (m, 1H), 7.51–7.44 (m, 2H), 7.17 (d, *J* = 9.1 Hz, 1H), 3.89 (q, *J* = 7.2 Hz, 1H), 3.77 (s, 3H), 3.68 (s, 3H), 1.60 (br d, *J* = 7.2 Hz, 3H); ^13^C­{^1^H} NMR (101 MHz, CDCl_3_): δ 174.9, 166.0, 157.8, 142.0, 139.5, 136.9, 136.8, 134.4, 132.5, 130.7, 129.6, 129.2, 129.1, 128.8, 127.3, 126.9, 124.6, 122.0, 119.3, 114.0, 57.0, 52.3, 45.2, 18.5; HRMS (ESI) *m*/*z*: [M + H]^+^ calcd for C_28_H_25_NO_6_SH, 504.1475; found, 504.1475.

### 5-(4′-*N*-Di-*n*-propylsulfonamido)-(*S*)-naproxen Methyl Ester (**17**)

To a solution of 5-(4′-aminophenylsulfonyl)-(*S*)-naproxen methyl ester (**14**) (0.121 g, 0.302 mmol) and triethylamine (126 μL, 0.904 mmol) in anhydrous dichloromethane (0.4 mL) was added propane-1-sulfonyl chloride (84.8 μL, 0.754 mmol) dropwise at 0 °C. The mixture was warmed to room temperature and stirred for 2.5 h. The reaction mixture was concentrated. Purification by normal phase chromatography (0–50% ethyl acetate in cyclohexane) gave 5-(4′-*N*-di-*n*-propylsulfonamido)-(*S*)-naproxen methyl ester (**17**) (0.143 g, 78%) as a white solid. Mp 161–163 °C; [α]_D_^19^ +19.1 (*c* 0.1, CHCl_3_); IR (neat) 2973, 1739, 1371, 1351, 1285, 1151, 1081, 918 cm^–1^; ^1^H NMR (400 MHz, CDCl_3_): δ 9.41 (d, *J* = 9.1 Hz, 1H), 8.06–8.00 (m, 3H), 7.72 (d, *J* = 2.1 Hz, 1H), 7.64 (dd, *J* = 9.2, 2.1 Hz, 1H), 7.49–7.44 (m, 2H), 7.18 (d, *J* = 9.1 Hz, 1H), 3.90 (q, *J* = 7.2 Hz, 1H), 3.70 (s, 3H), 3.69 (s, 3H), 3.55–3.49 (m, 4H), 1.98–1.86 (m, 4H), 1.61 (d, *J* = 7.2 Hz, 3H), 1.07 (t, *J* = 7.4 Hz, 6H); ^13^C­{^1^H} NMR (101 MHz, CDCl_3_): δ 174.8, 157.8, 146.1, 137.5, 137.23, 137.19, 131.4, 130.6, 129.6, 129.4, 128.4, 127.0, 124.5, 121.3, 114.0, 57.5, 56.8, 52.3, 45.2, 18.5, 17.1, 13.0; HRMS (ESI) *m*/*z*: [M + H]^+^ calcd for C_27_H_32_NO_9_S_3_H, 612.1390; found, 612.1387.

## Supplementary Material



## Data Availability

The data underlying this study are available in the published article and its online Supporting Information.

## Notes

^1^H and ^13^C NMR spectra of all compounds (PDF)
