# Supplementary material for: Accelerated Thioarylation of Arenes Using Lewis Acid and Lewis Base Dual Catalysis
Source: J Org Chem. 2026 May 18;91(21):7338–48. doi: 10.1021/acs.joc.6c00744 (PMC13227468; doi:10.1021/acs.joc.6c00744)

Supporting Information for:

Accelerated Thioarylation of Arenes using Lewis Acid and Lewis Base Dual Catalysis

Oluwajuwon A. M. Okunade,† Pankaj K. Majhi,† R. Nisha Khanizeman‡

and Andrew Sutherland*†

†School of Chemistry, The Joseph Black Building, University of Glasgow, Glasgow G12 8QQ, United Kingdom. ‡GSK Medicines Research Centre, Gunnels Wood Road, Stevenage, SG1 2NY, UK. Email: Andrew.Sutherland@glasgow.ac.uk

Table of Contents

1. ^{1}H and ^{13}C NMR Spectra of all Compounds S2–S67

1. ^{1}H and ^{13}C NMR Spectra of all Compounds

^{1}H NMR (400 MHz, $CDCl_3$)

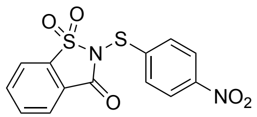

8a

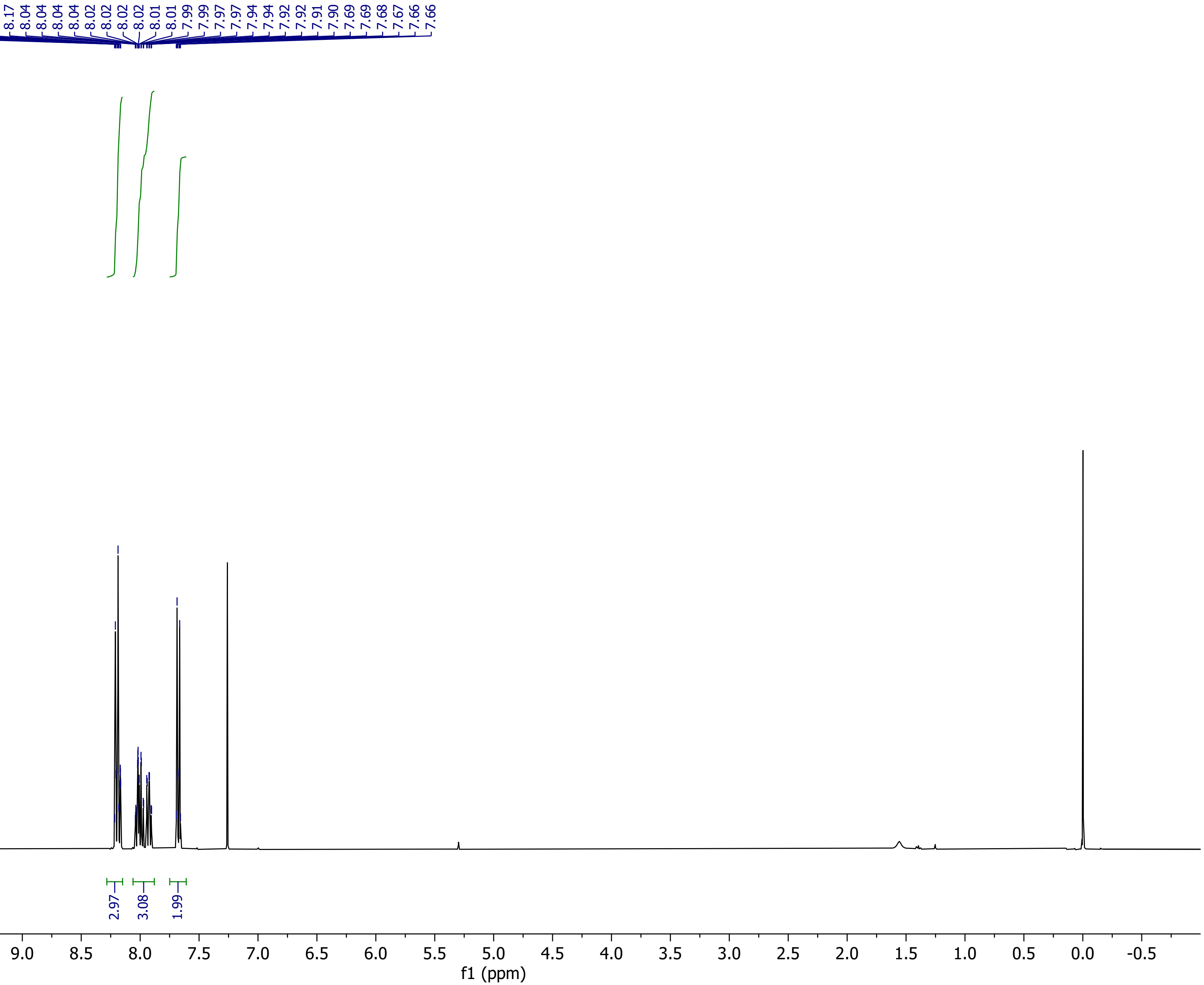

$^{13}C\{^{1}H\}$ NMR (101 MHz, $CDCl_3$)

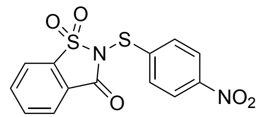

8a

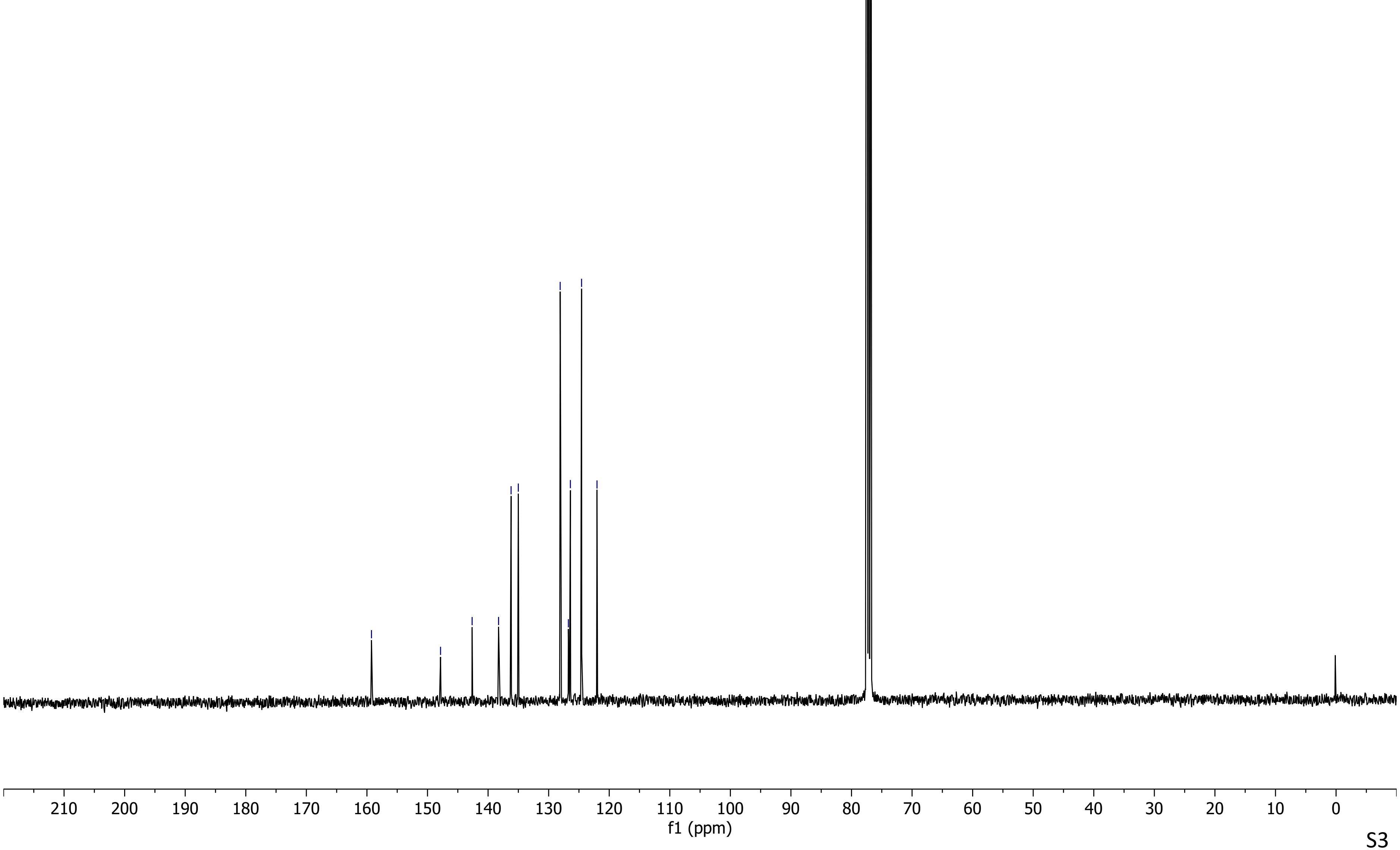

^{1}H NMR (400 MHz, $CDCl_3$)

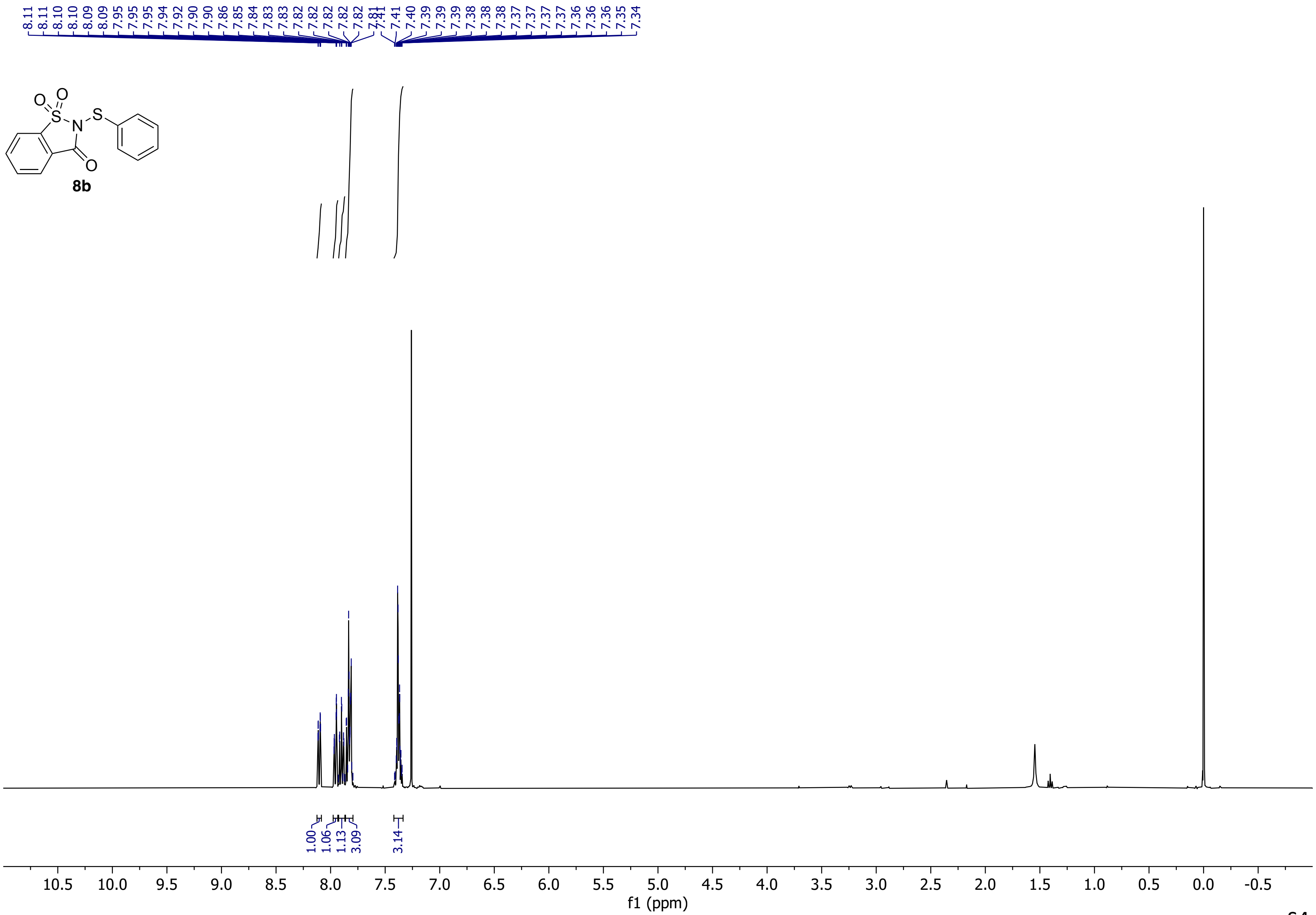

$^{13}C\{^1H\}$ NMR (101 MHz, $CDCl_3$)

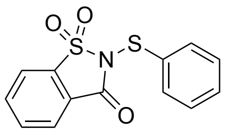

8b

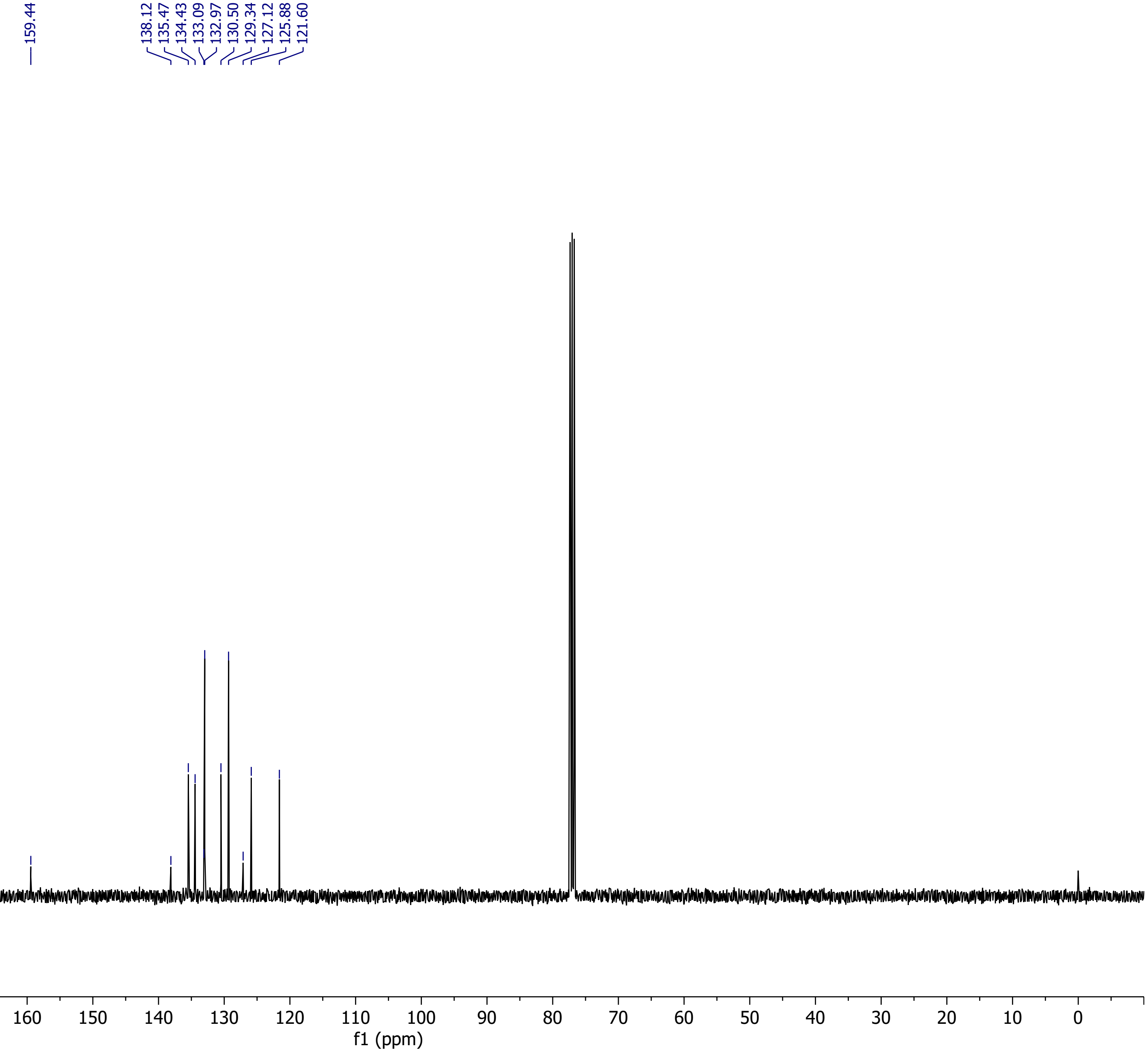

^{1}H NMR (400 MHz, $CDCl_3$)

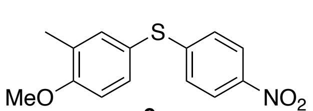

9a

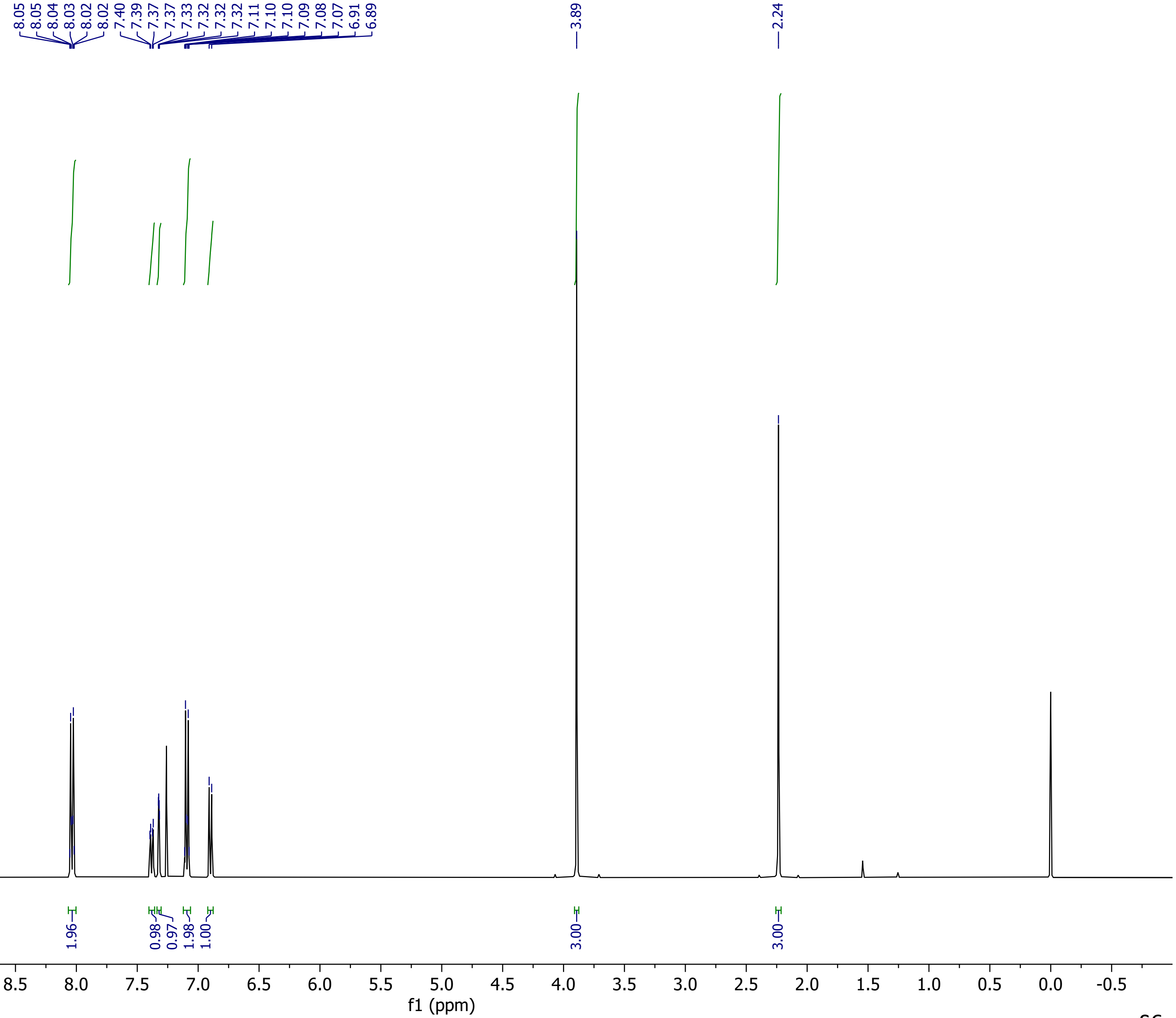

$^{13}C\{^1H\}$ NMR (101 MHz, $CDCl_3$)

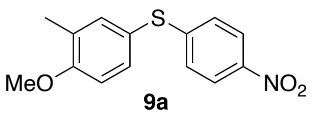

9a

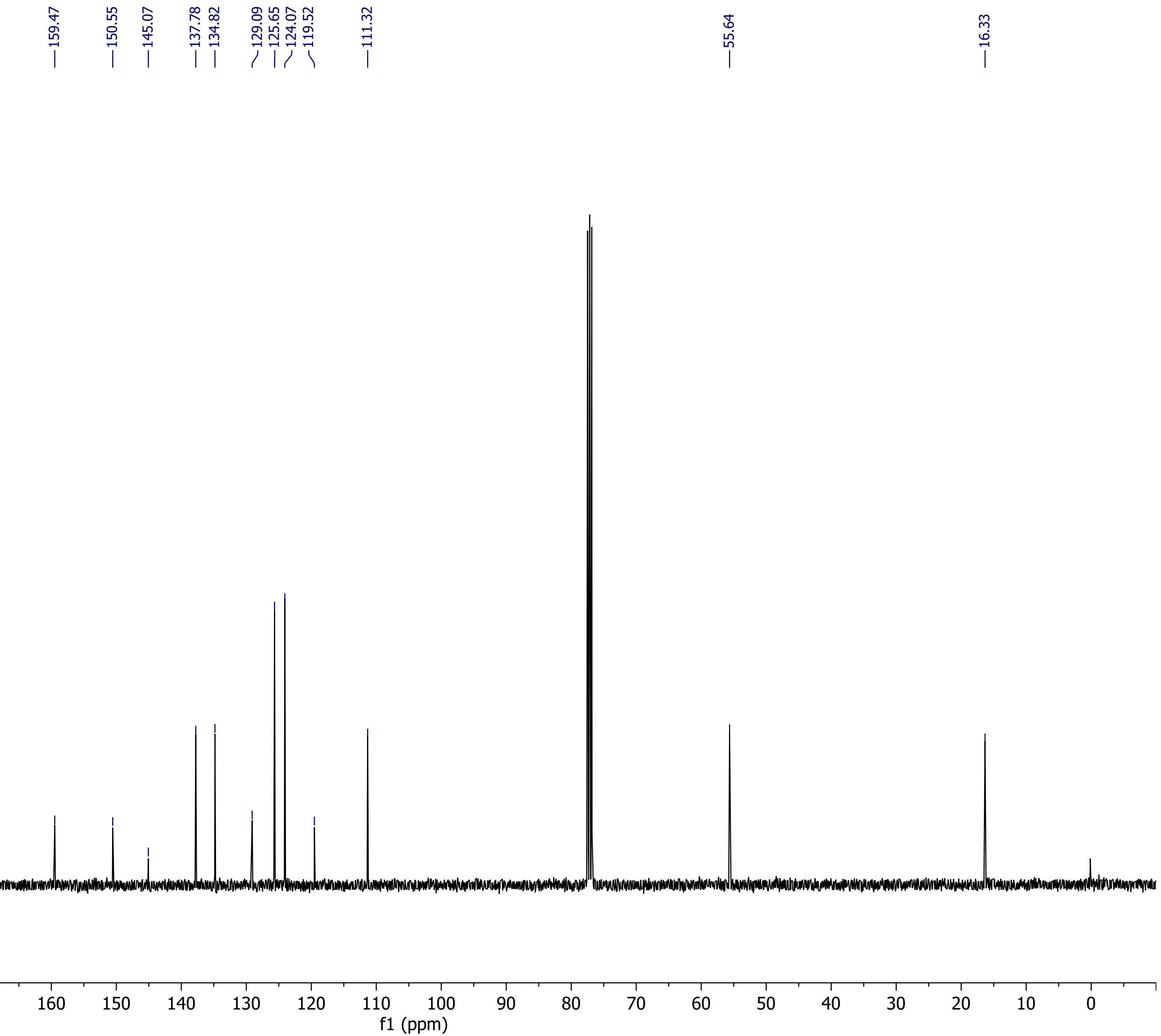

^{1}H NMR (400 MHz, $CDCl_3$)

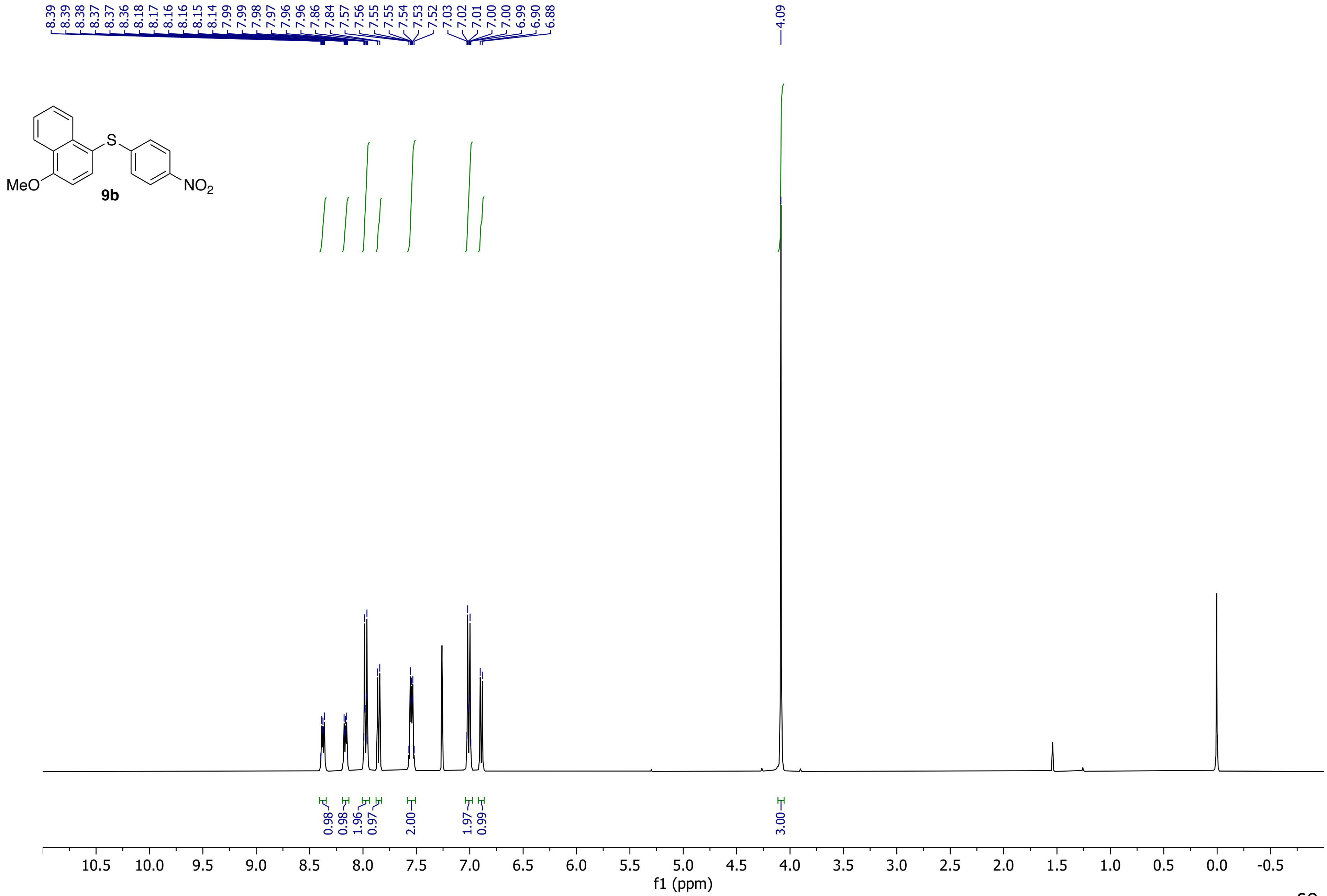

$^{13}C\{^1H\}$ NMR (101 MHz, $CDCl_3$)

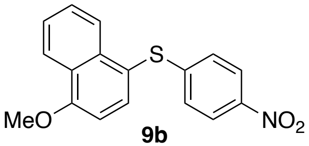

9b

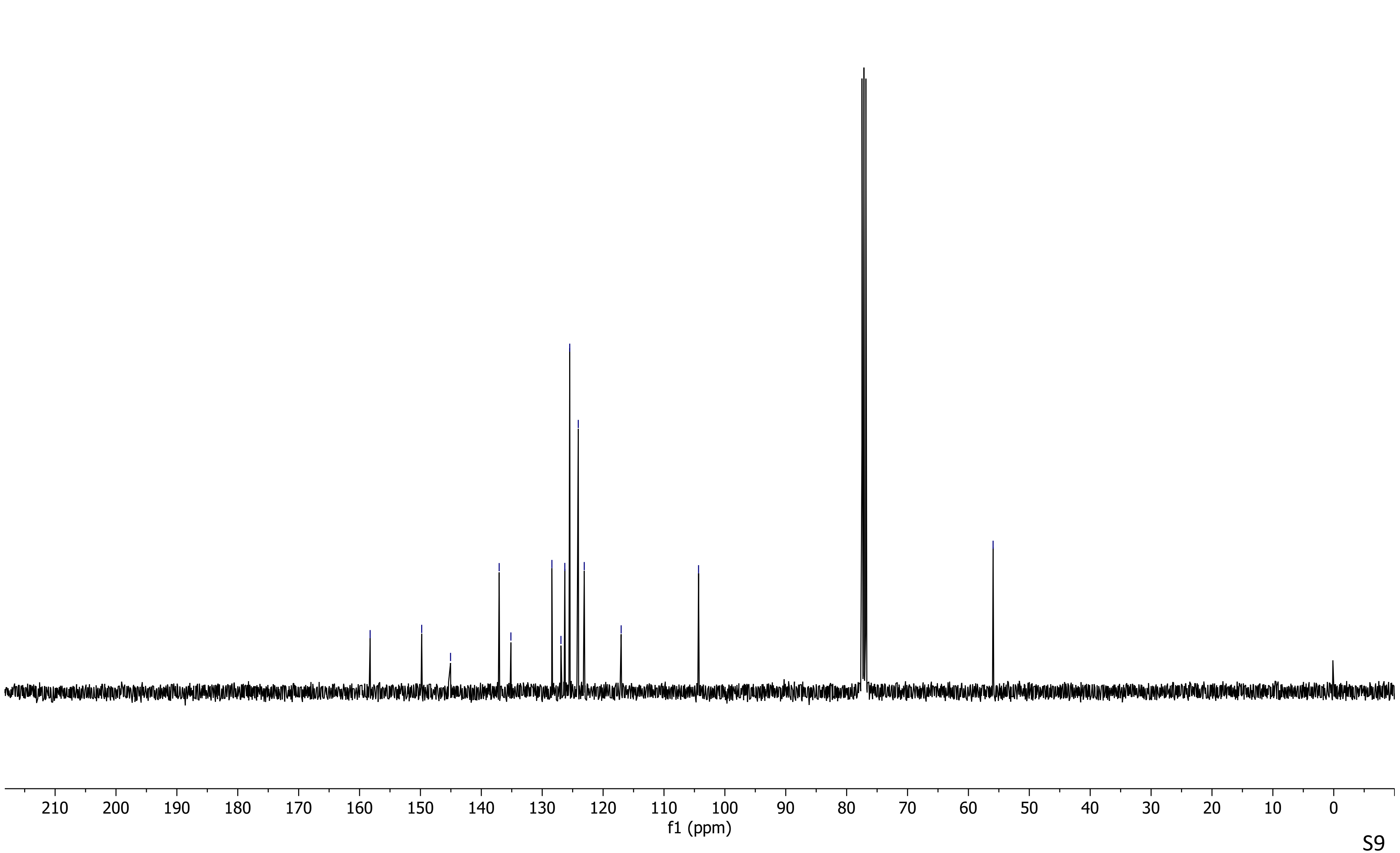

^{1}H NMR (400 MHz, CDCl$_3$)

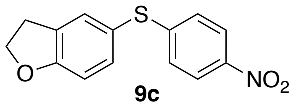

9c

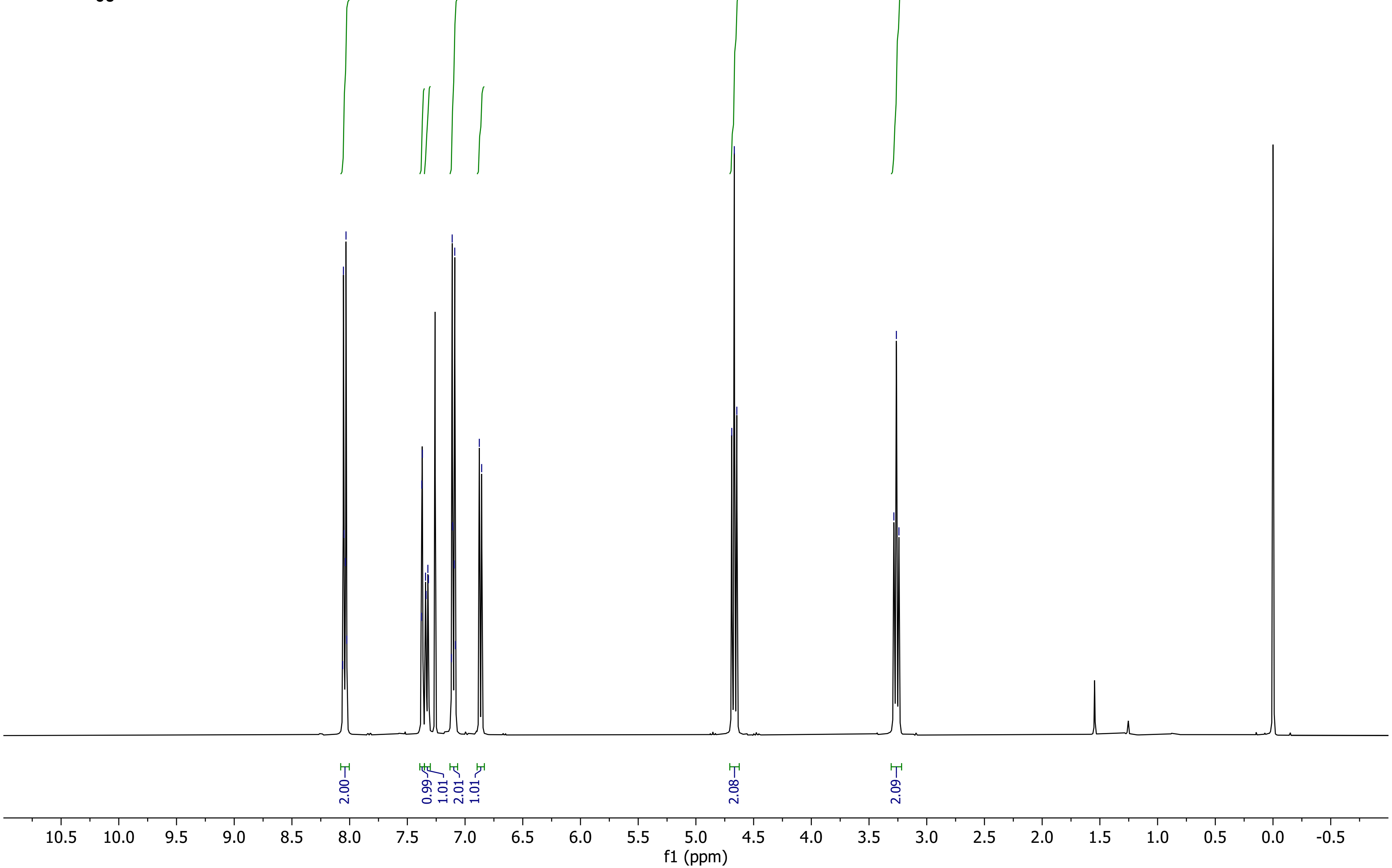

$^{13}C\{^1H\}$ **NMR (101 MHz, $CDCl_3$)**

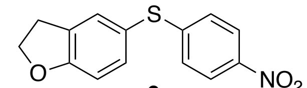

9c

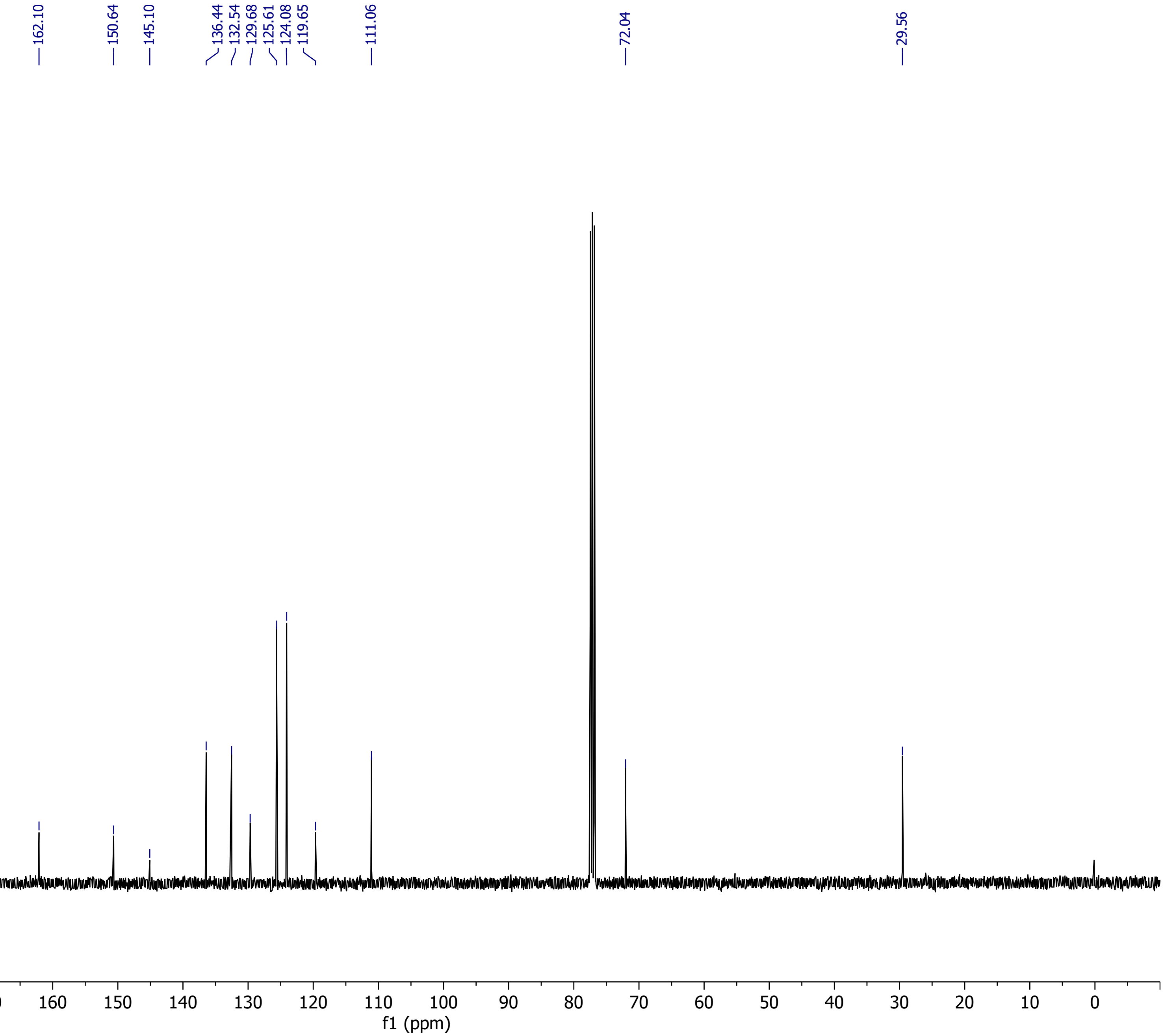

^{1}H NMR (400 MHz, $CDCl_3$)

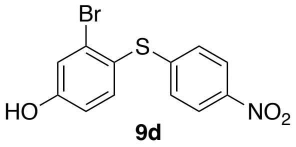

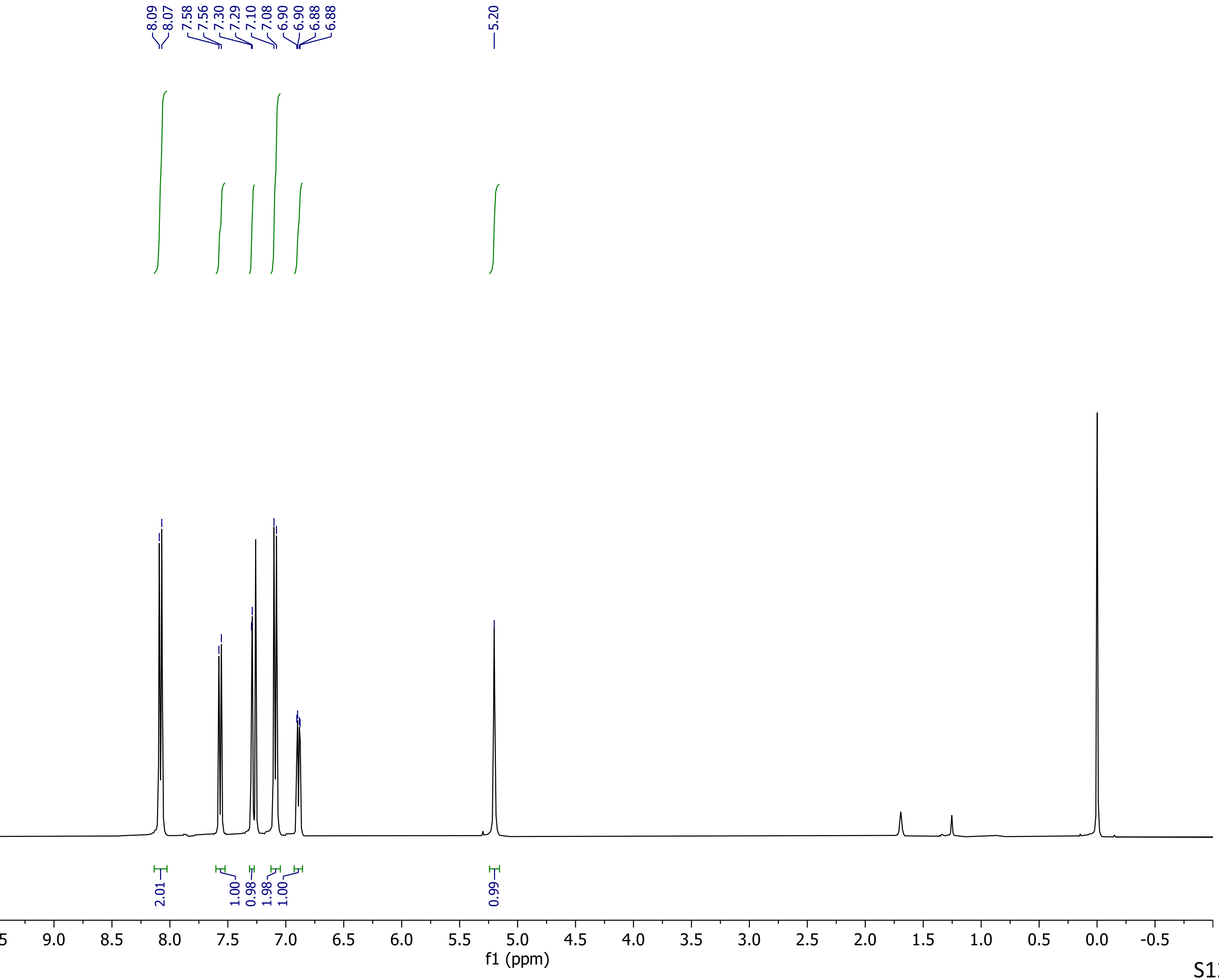

$^{13}C\{^{1}H\}$ NMR (101 MHz, $CDCl_3$)

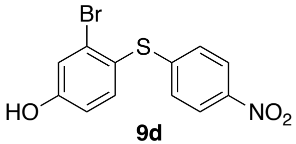

9d

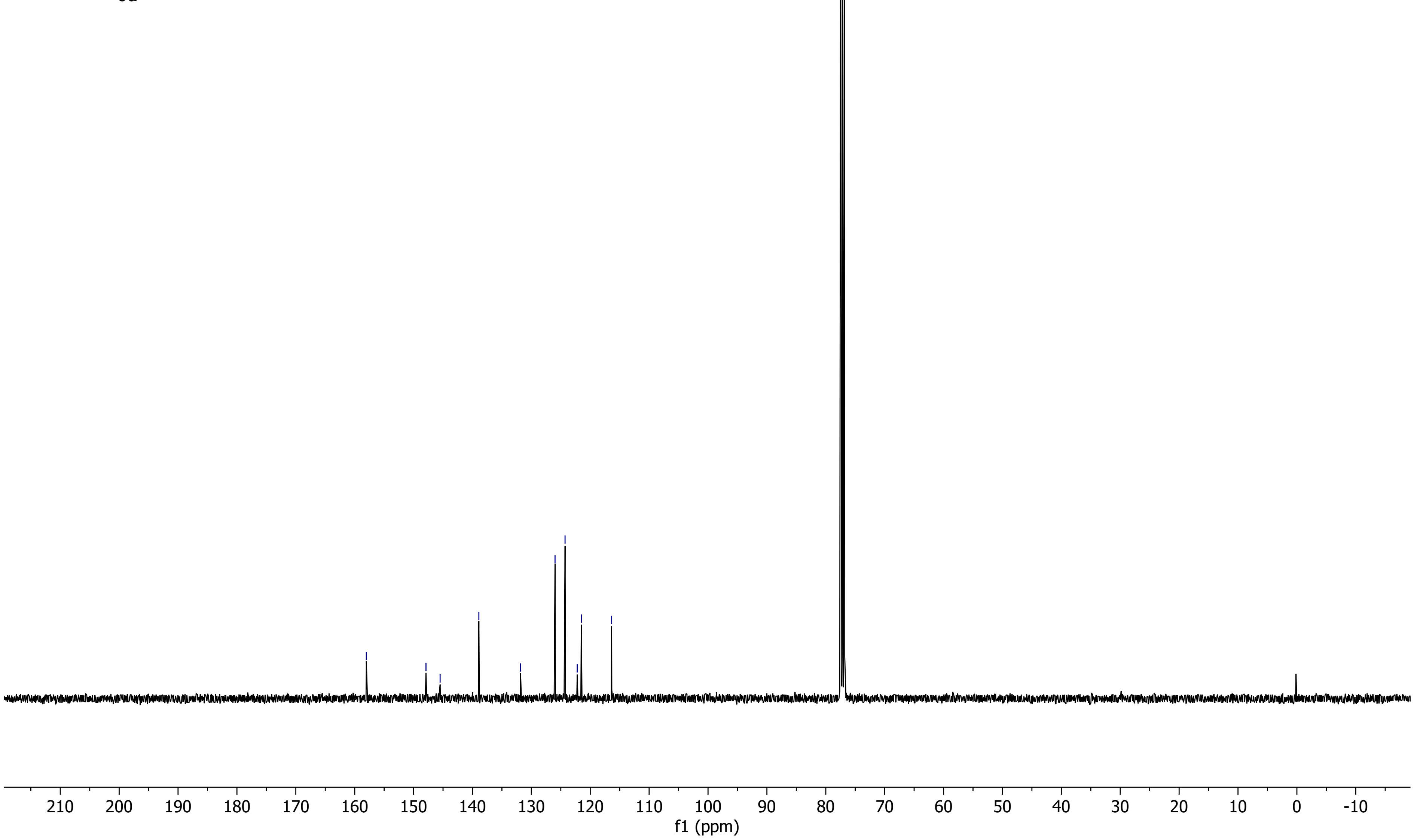

^{1}H NMR (400 MHz, $CDCl_3$)

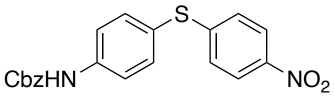

9e

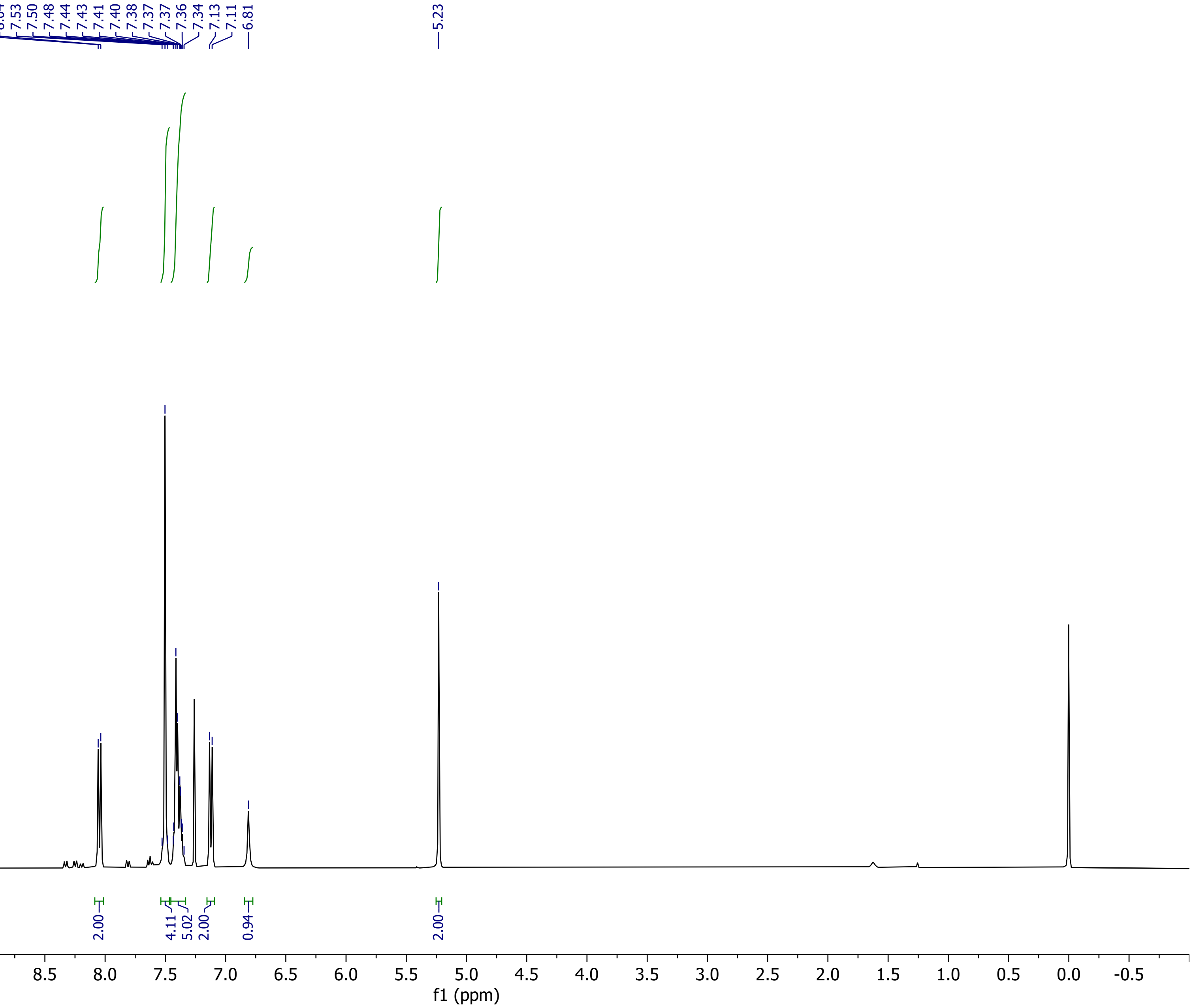

$^{13}C\{^{1}H\}$ NMR (101 MHz, CDCl$_3$)

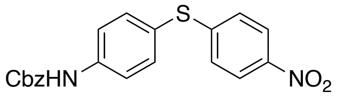

9e

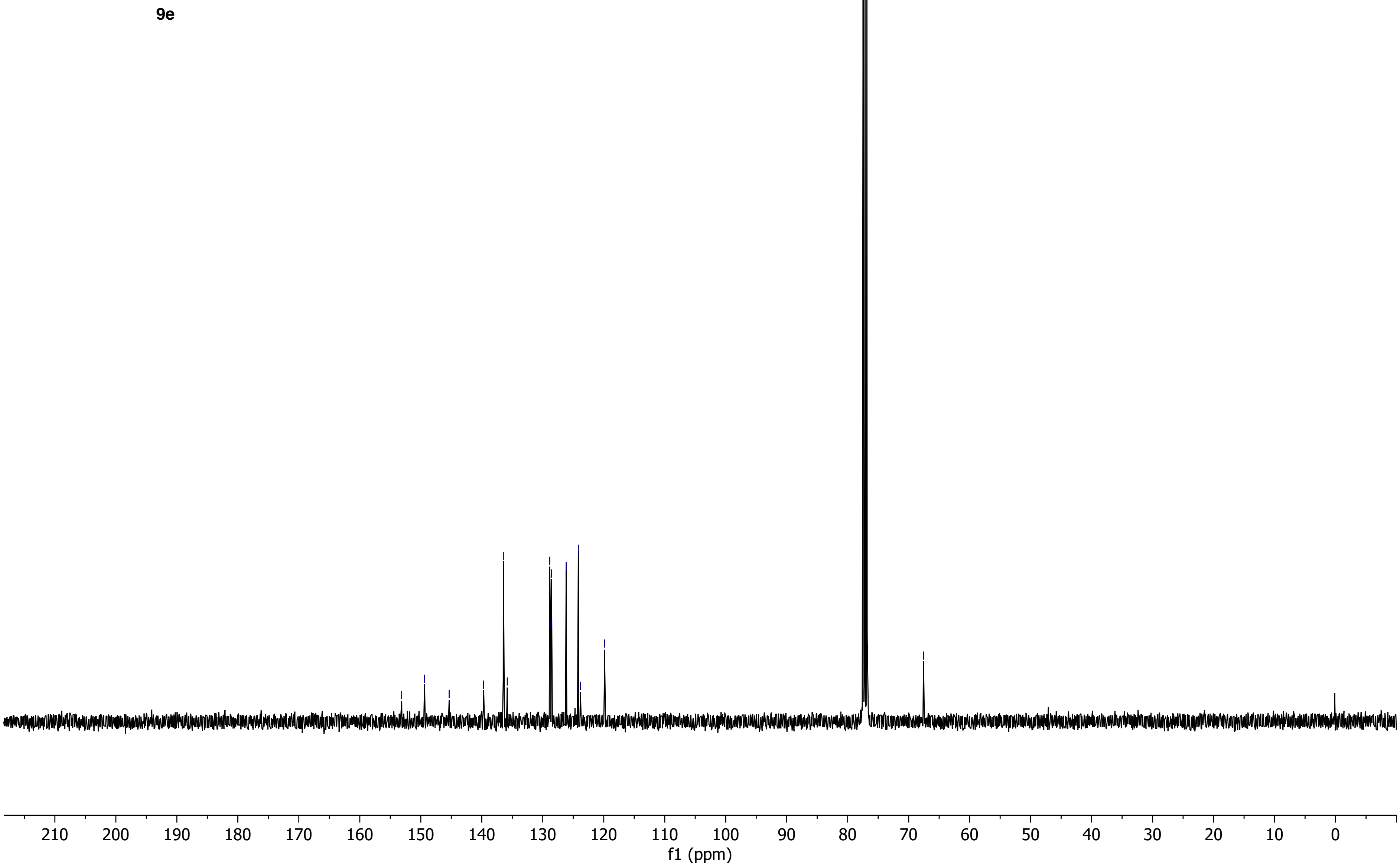

^{1}H NMR (400 MHz, $CDCl_3$)

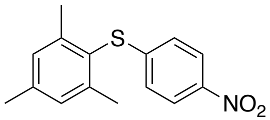

9f

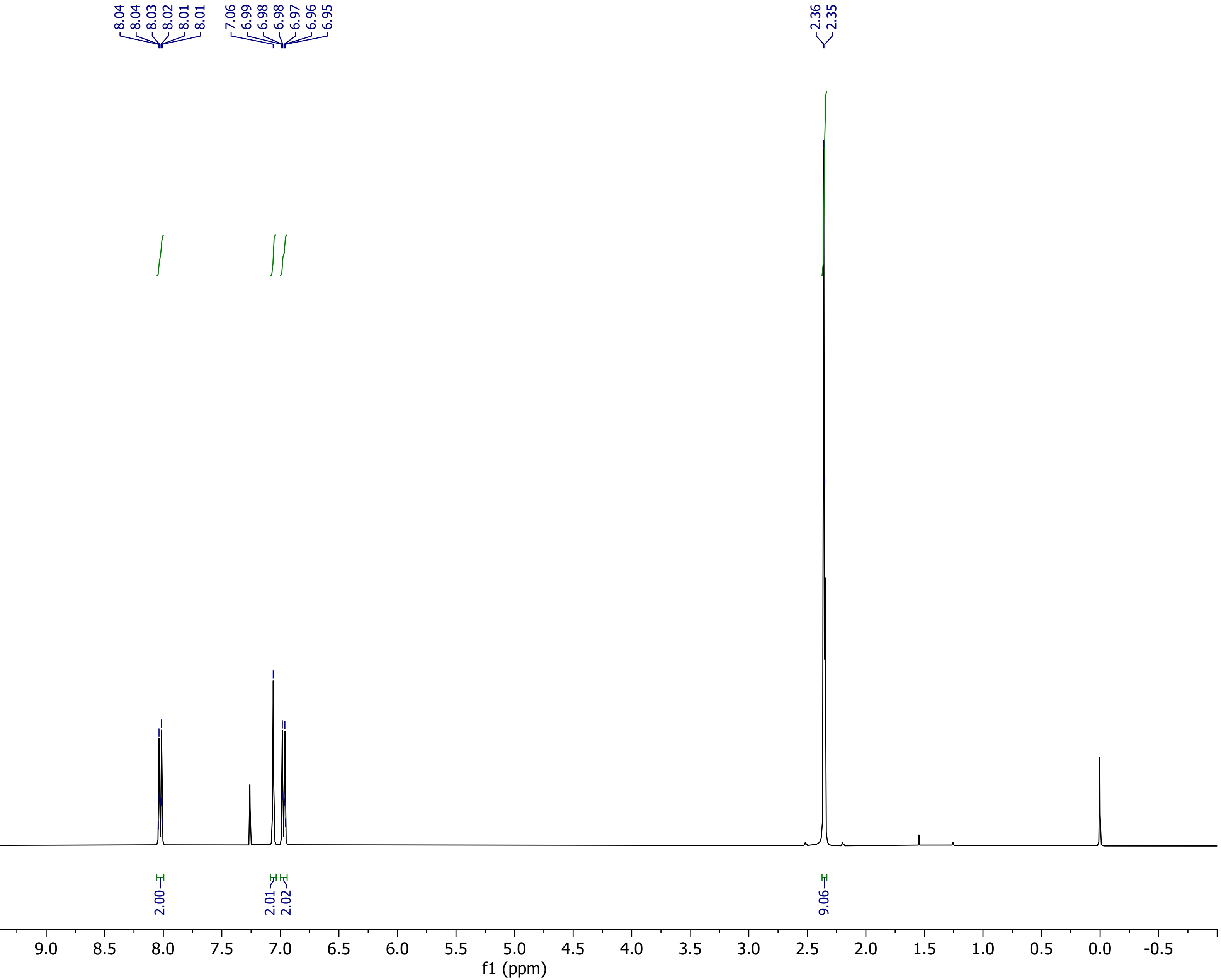

$^{13}C\{^{1}H\}$ NMR (101 MHz, $CDCl_3$)

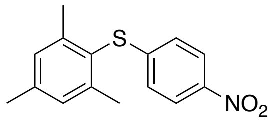

9f

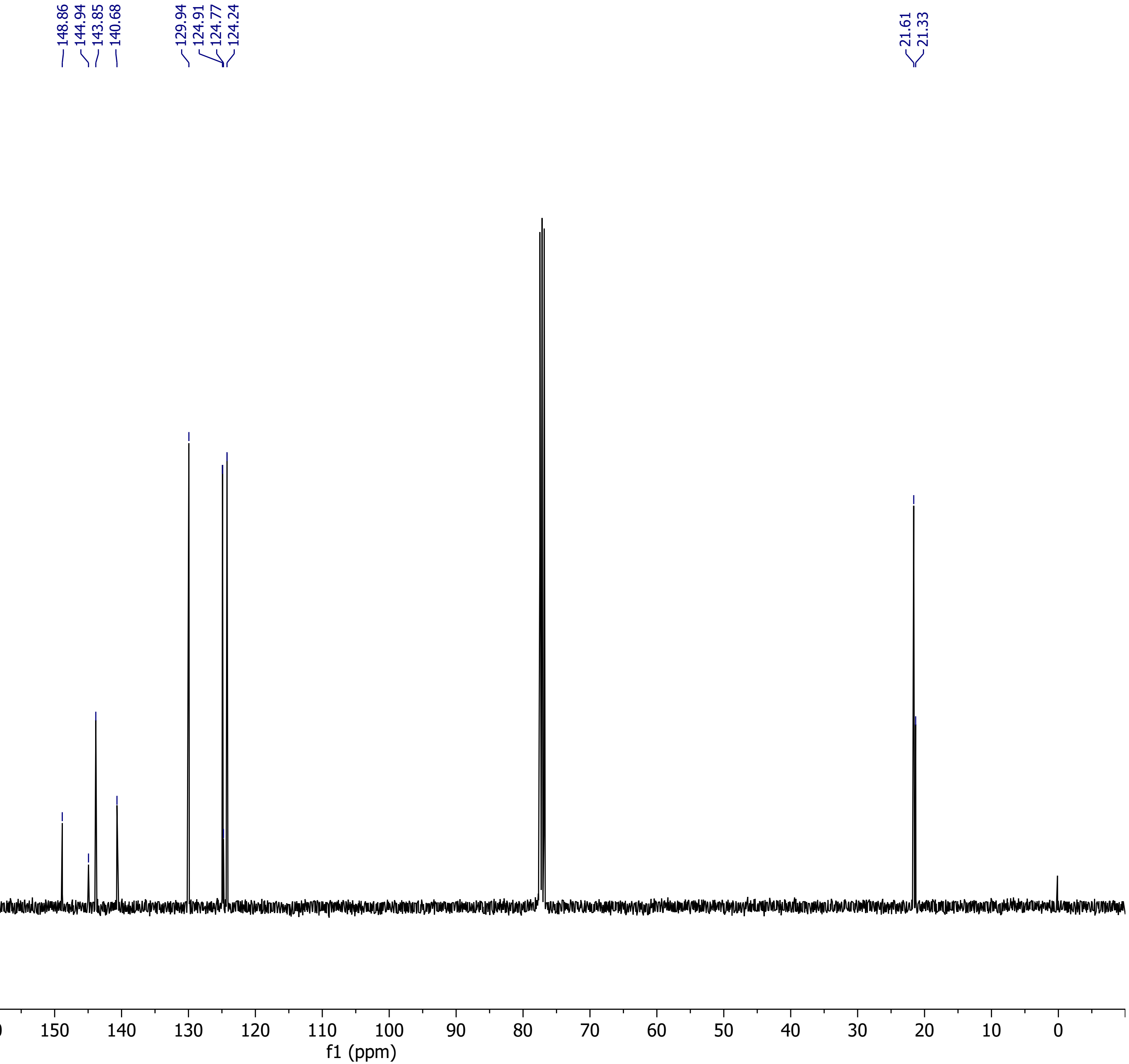

^{1}H NMR (400 MHz, DMSO-d_6)

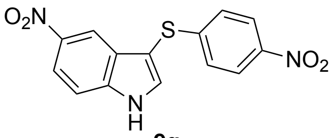

9g

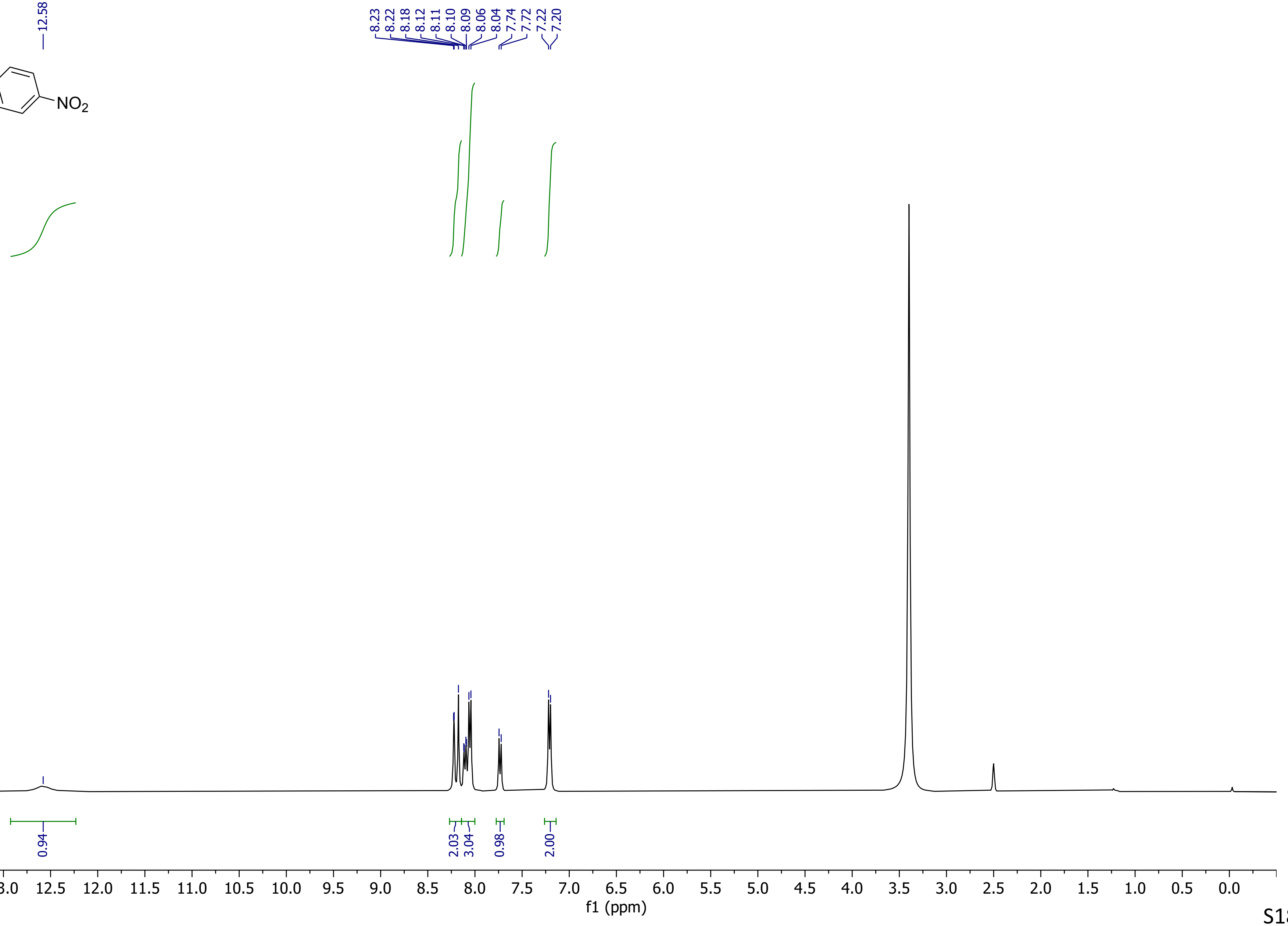

$^{13}C\{^{1}H\}$ NMR (101 MHz, DMSO-d_6)

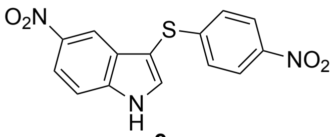

9g

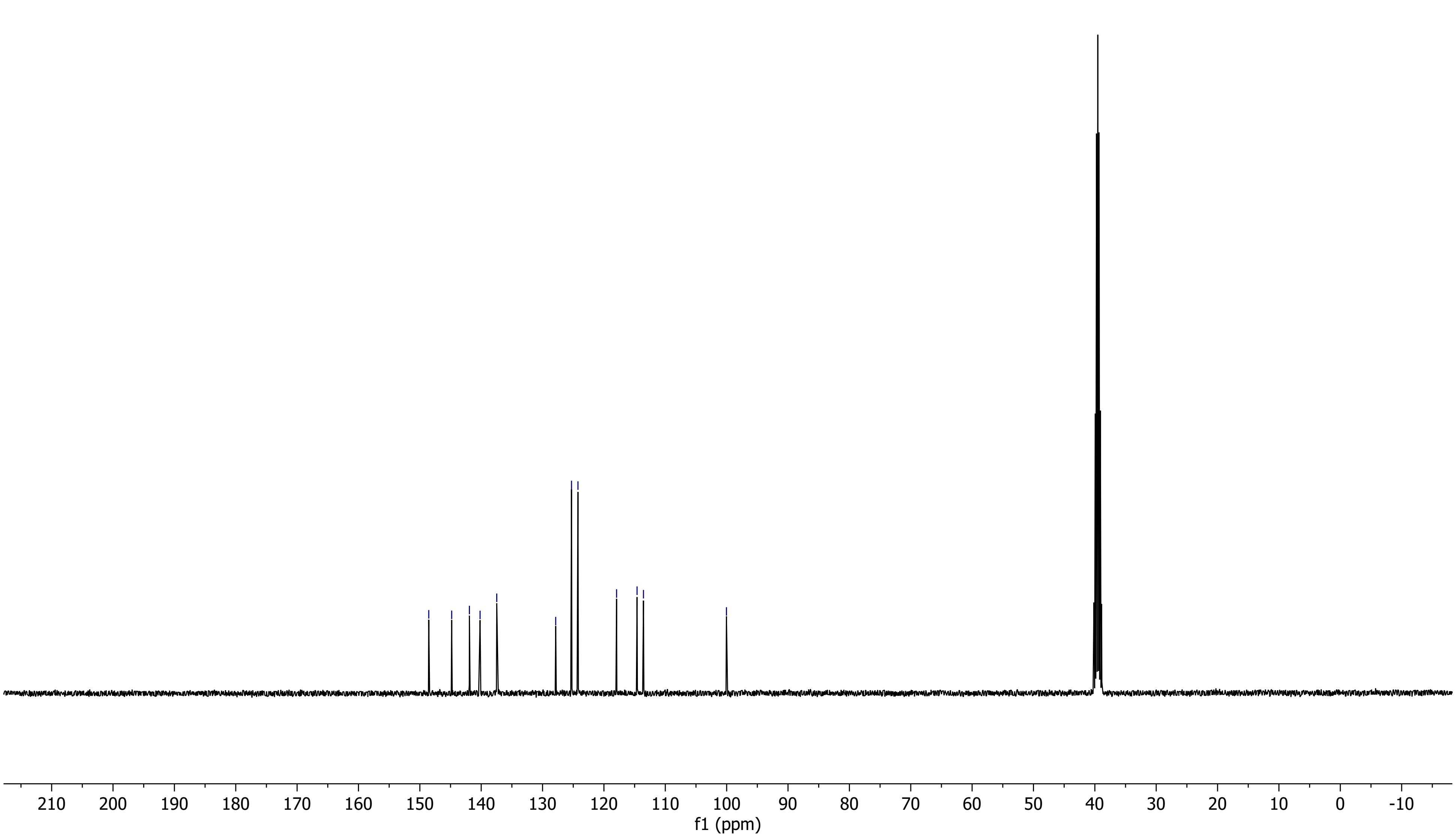

^{1}H NMR (400 MHz, $CDCl_3$)

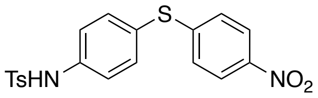

9h

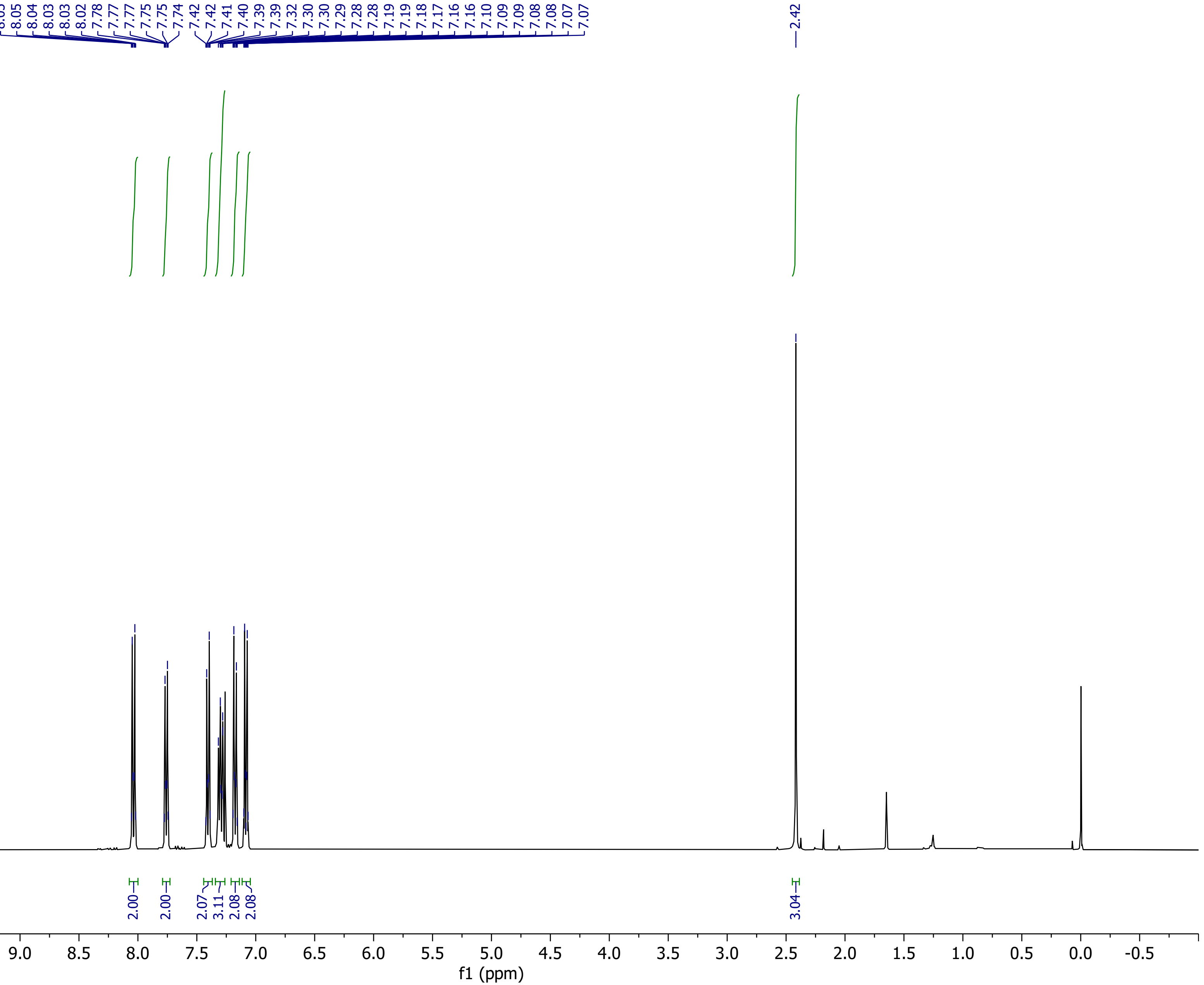

$^{13}C\{^{1}H\}$ NMR (101 MHz, $CDCl_3$)

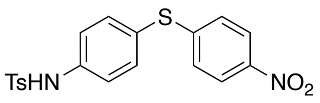

9h

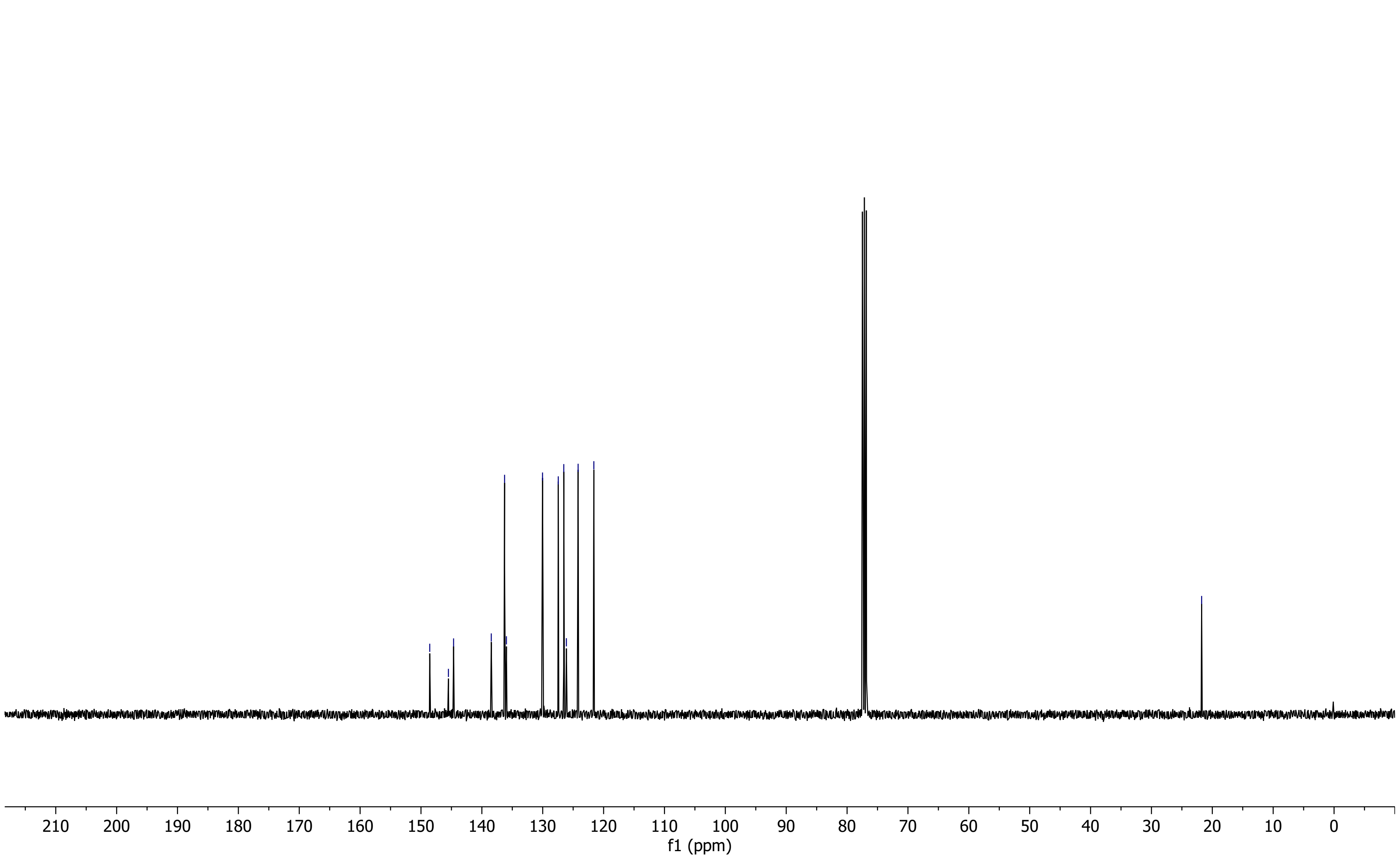

^{1}H NMR (400 MHz, $CDCl_3$)

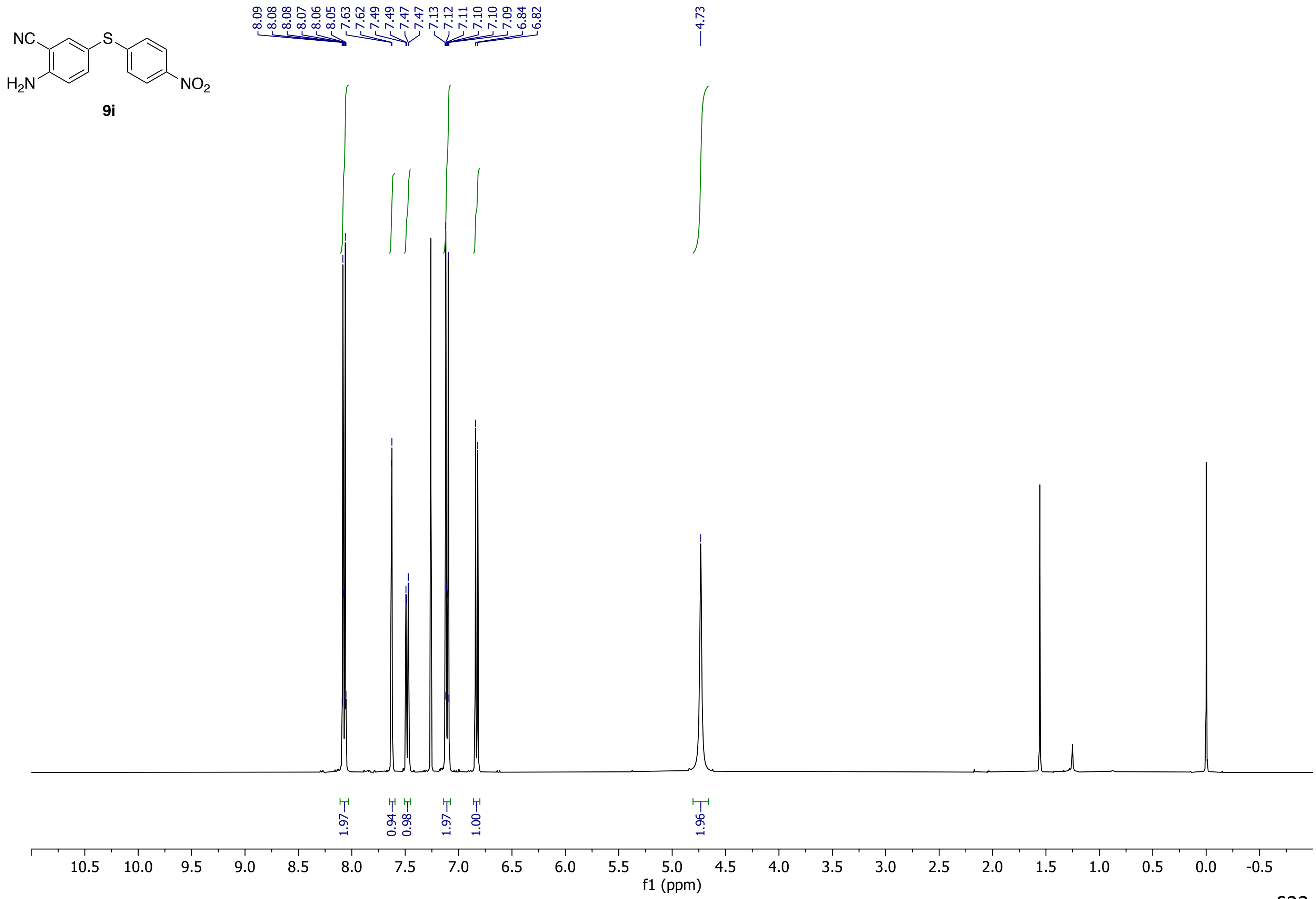

$^{13}C\{^1H\}$ NMR (101 MHz, $CDCl_3$)

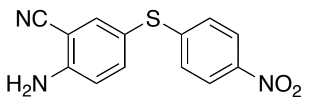

9i

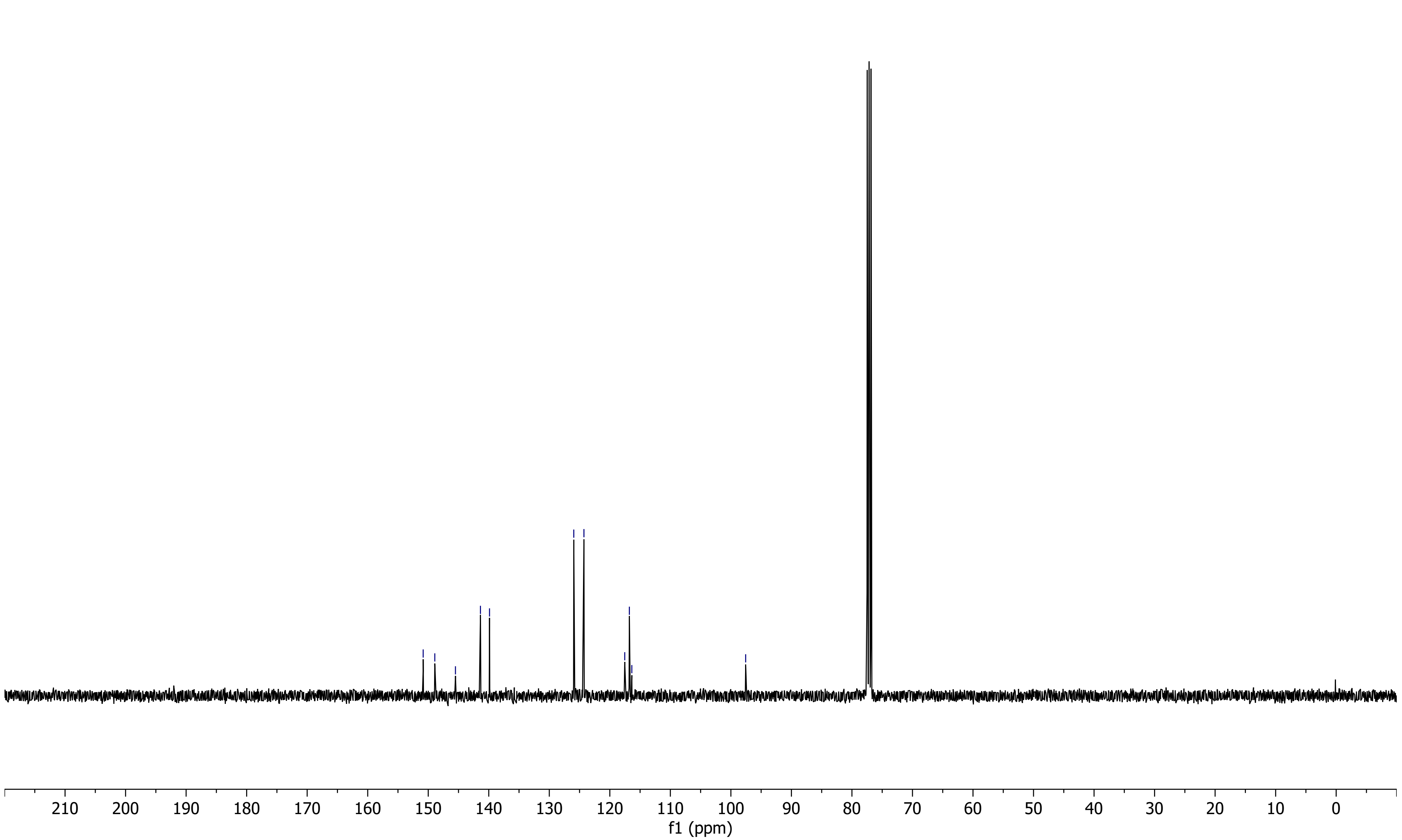

^{1}H NMR (400 MHz, $CDCl_3$)

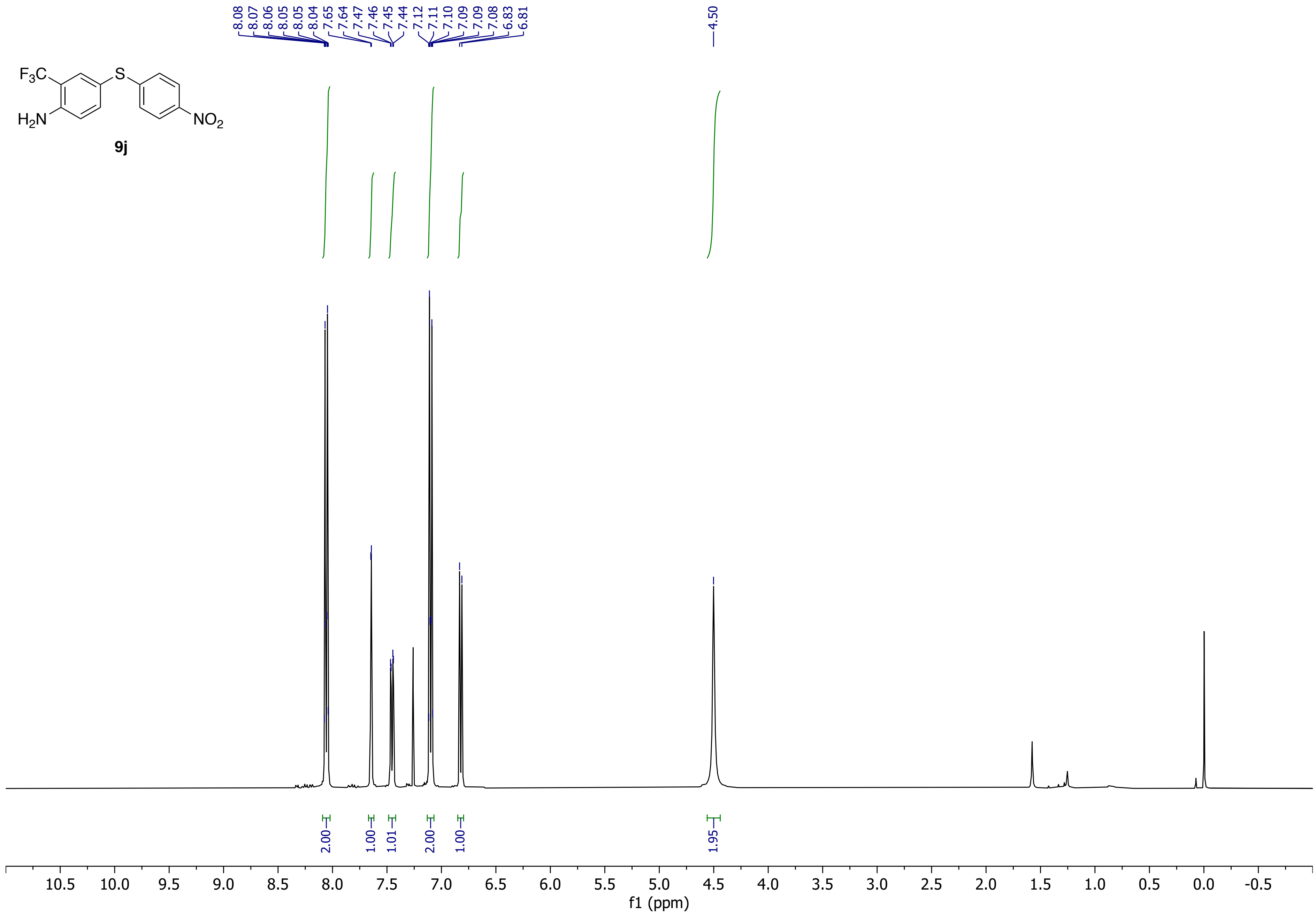

$^{13}C\{^1H\}$ NMR (101 MHz, $CDCl_3$)

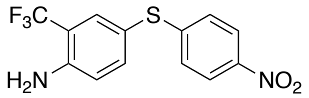

9j

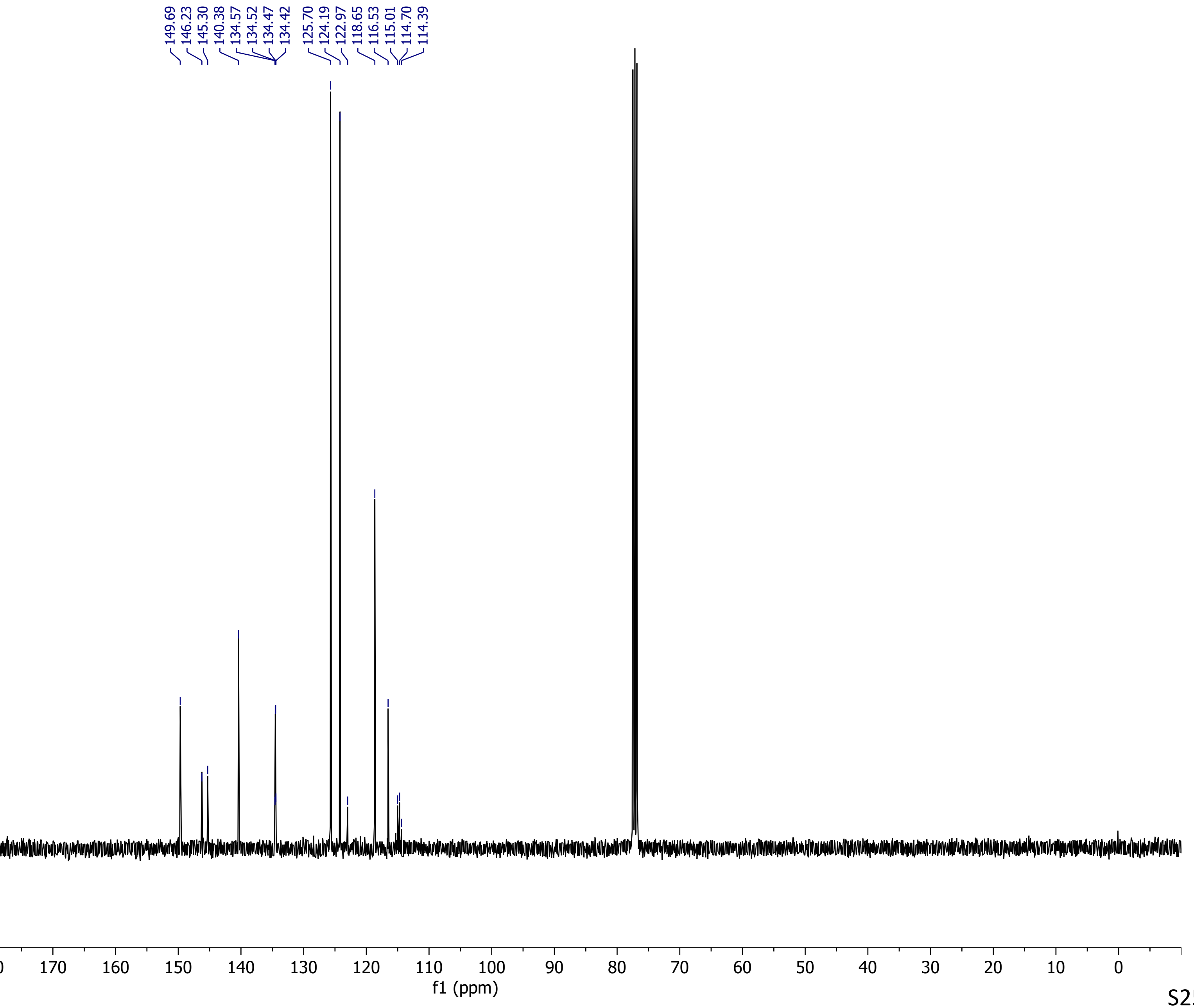

^{1}H NMR (400 MHz, $CDCl_3$)

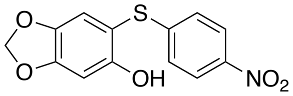

9k

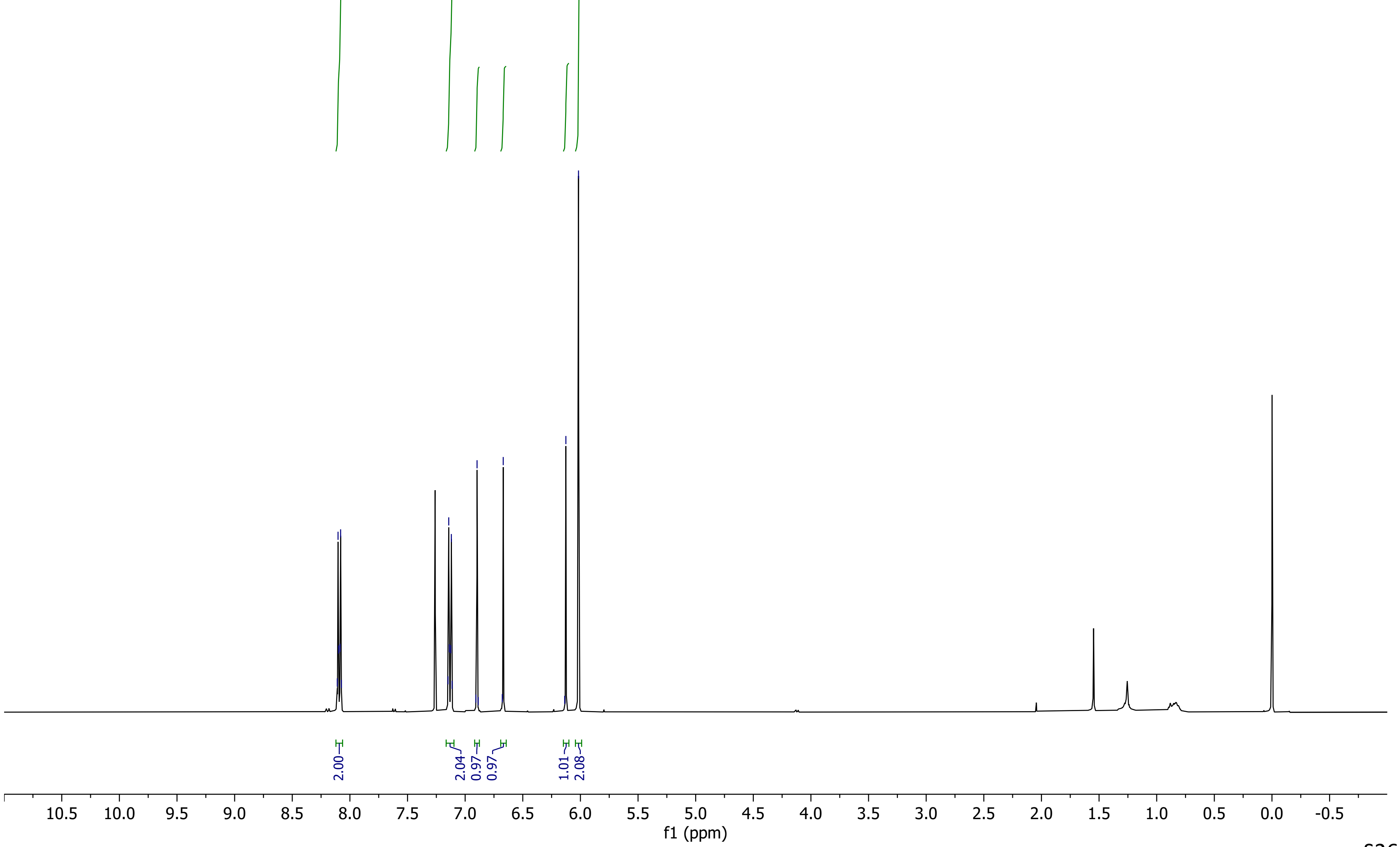

$^{13}C\{^1H\}$ NMR (101 MHz, $CDCl_3$)

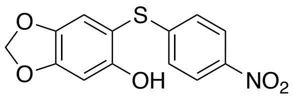

9k

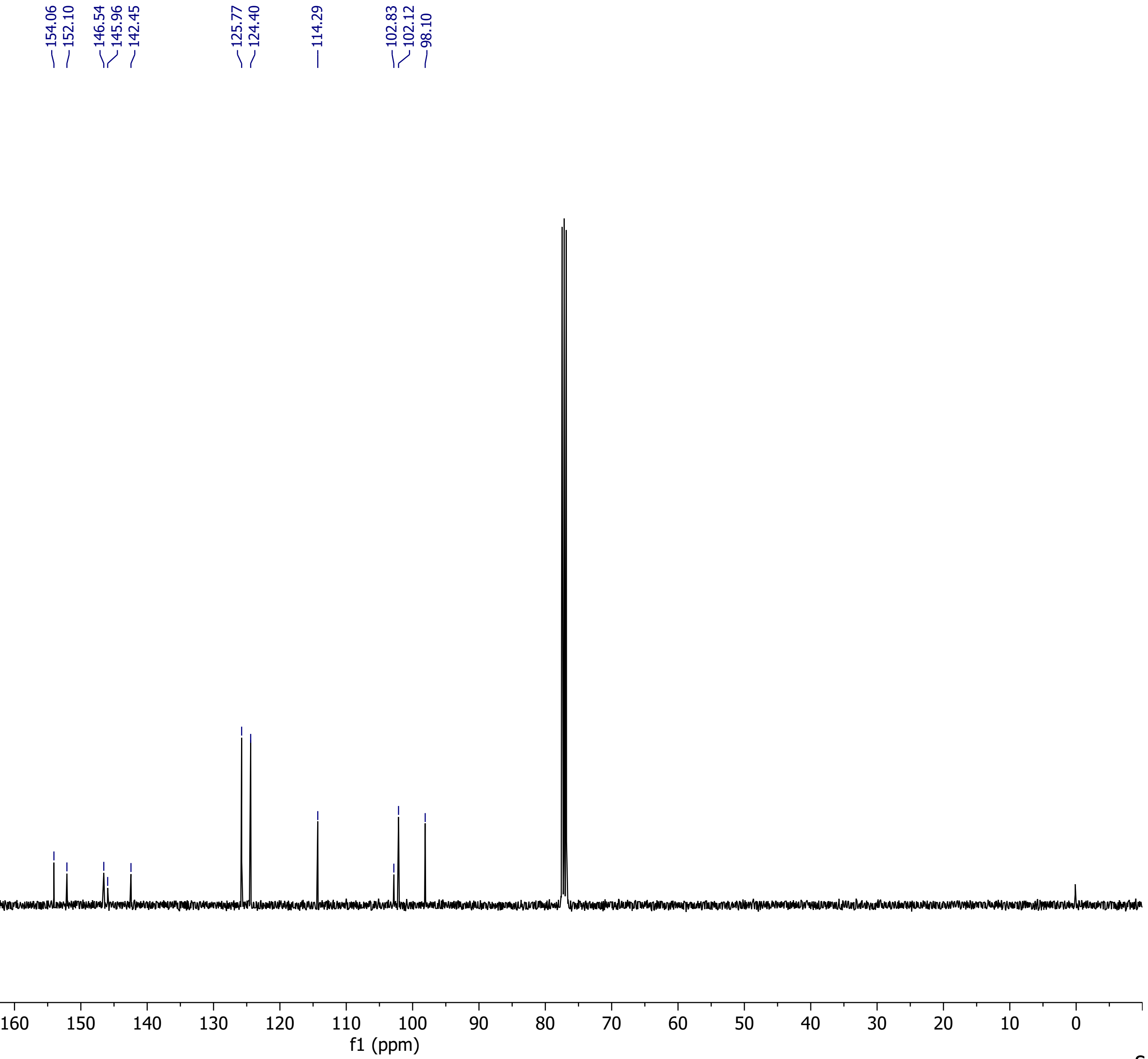

^{1}H NMR (400 MHz, $CDCl_3$)

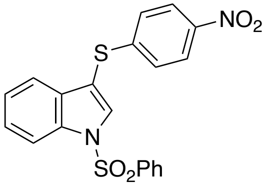

9l

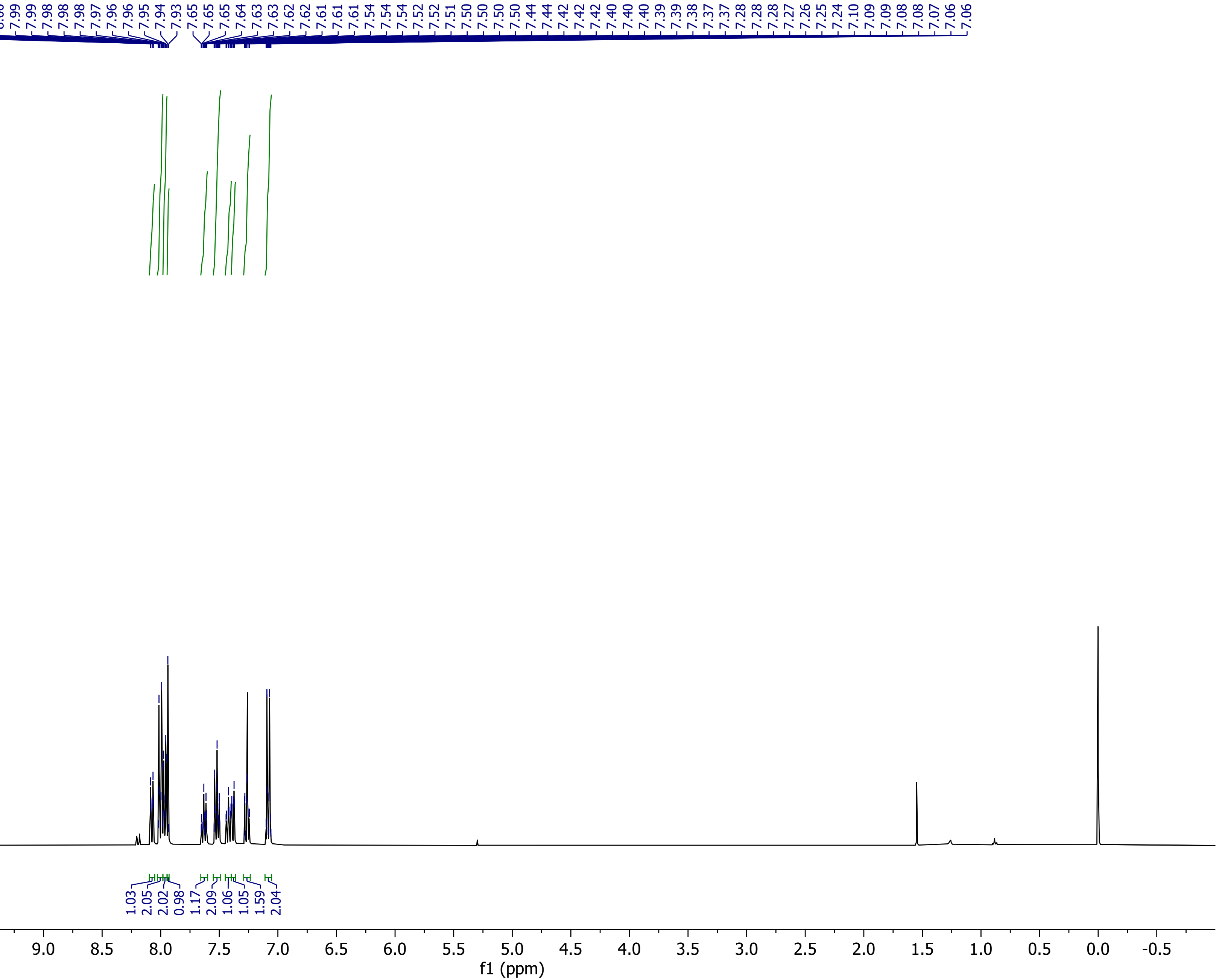

$^{13}C\{^{1}H\}$ NMR (101 MHz, $CDCl_3$)

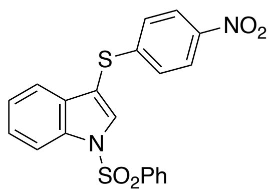

9l

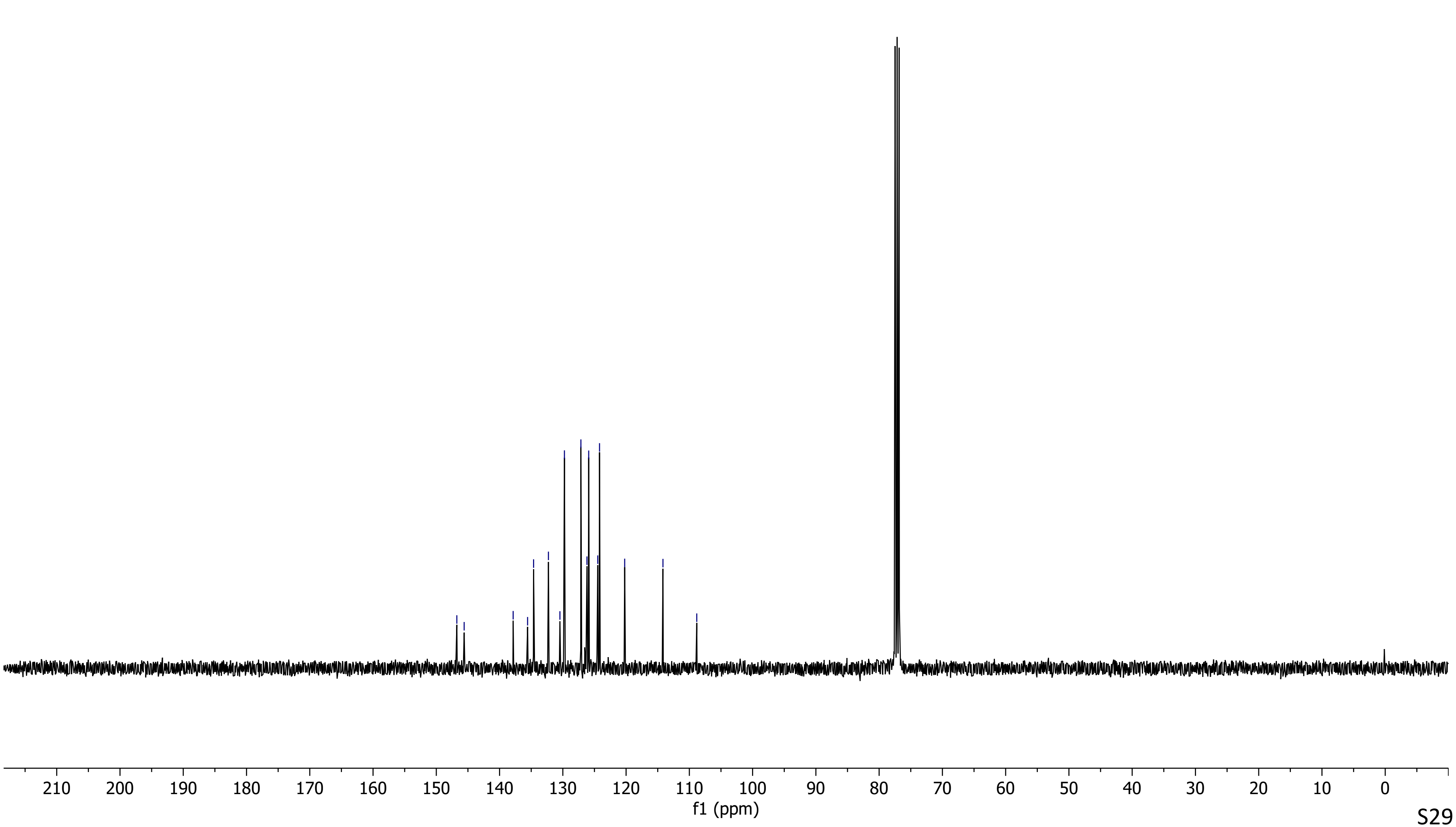

^{1}H NMR (400 MHz, $CDCl_3$)

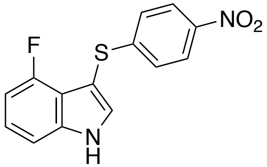

9m

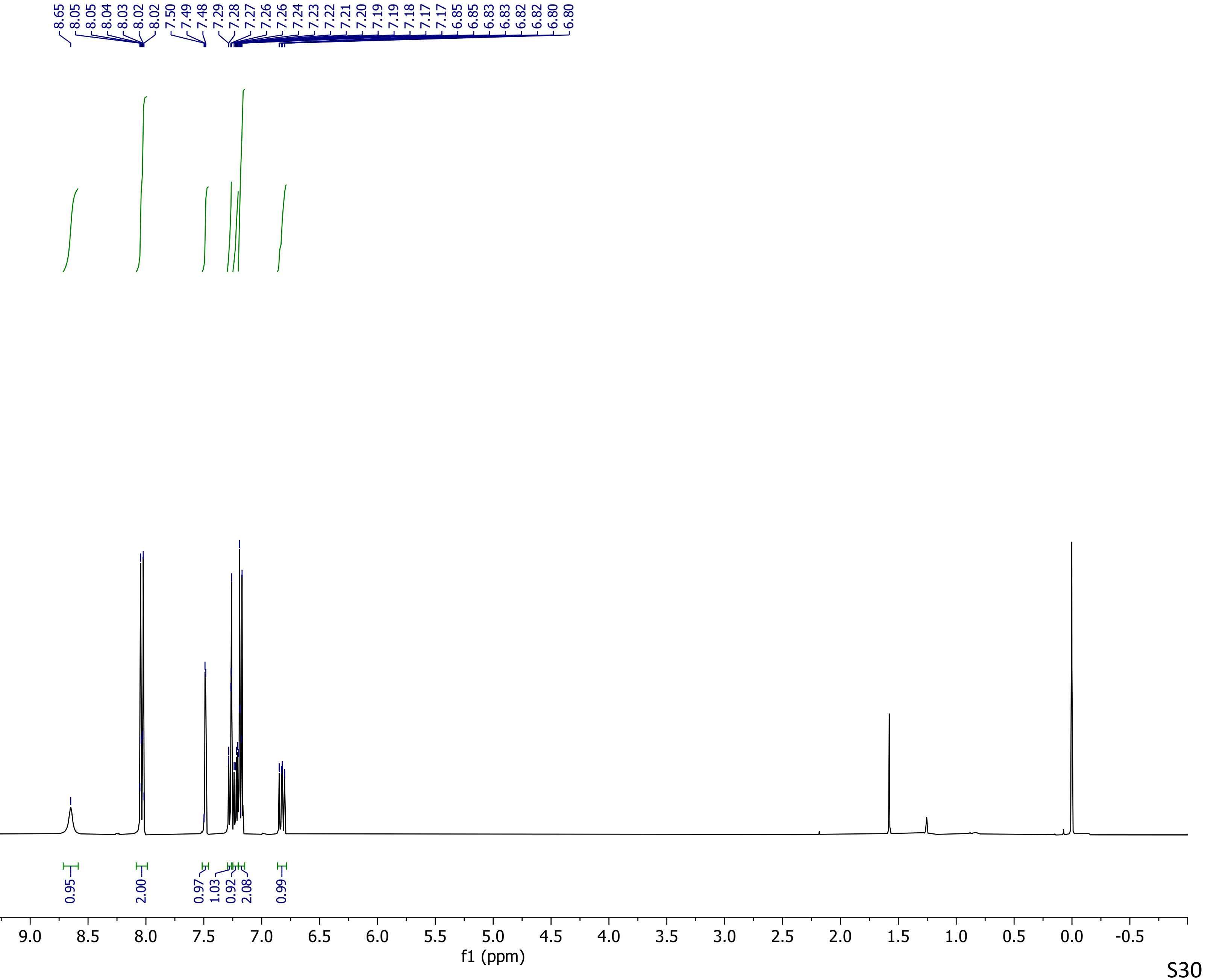

$^{13}C\{^{1}H\}$ NMR (101 MHz, $CDCl_3$)

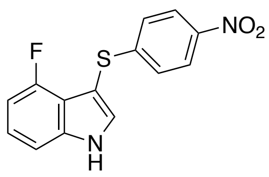

9m

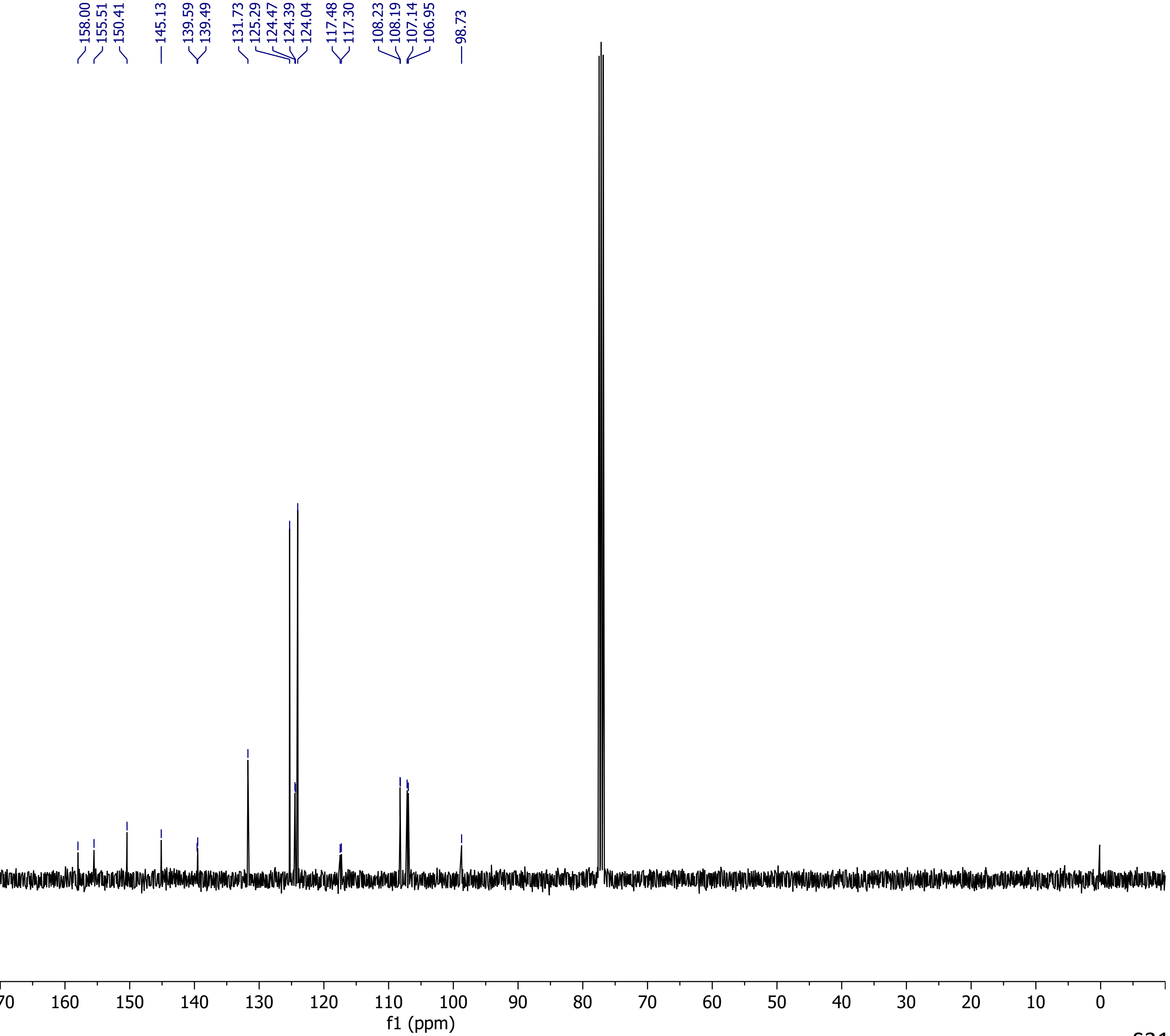

^{1}H NMR (400 MHz, $CDCl_3$)

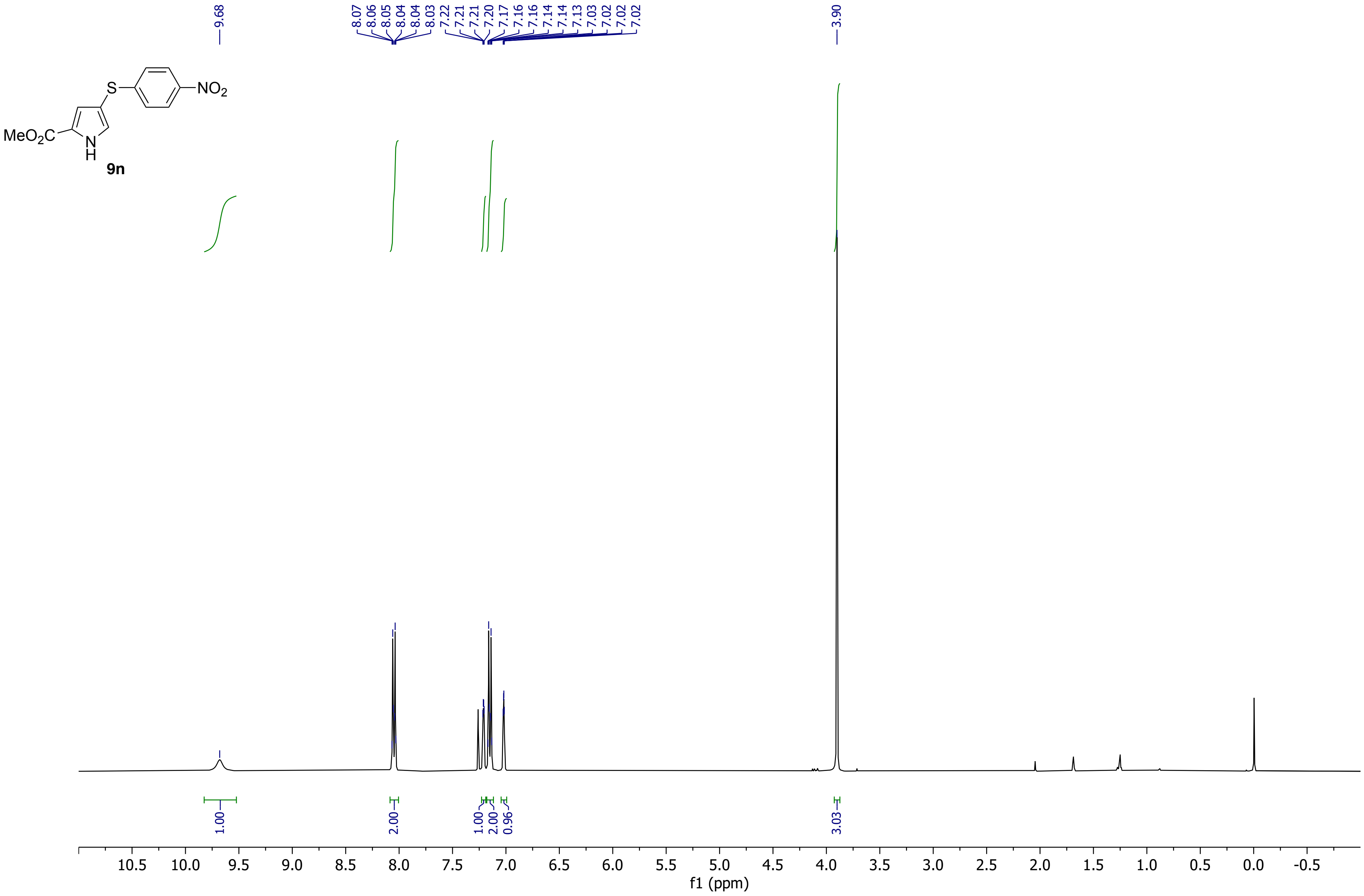

$^{13}C\{^1H\}$ NMR (101 MHz, $CDCl_3$)

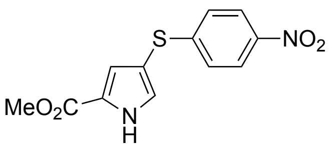

9n

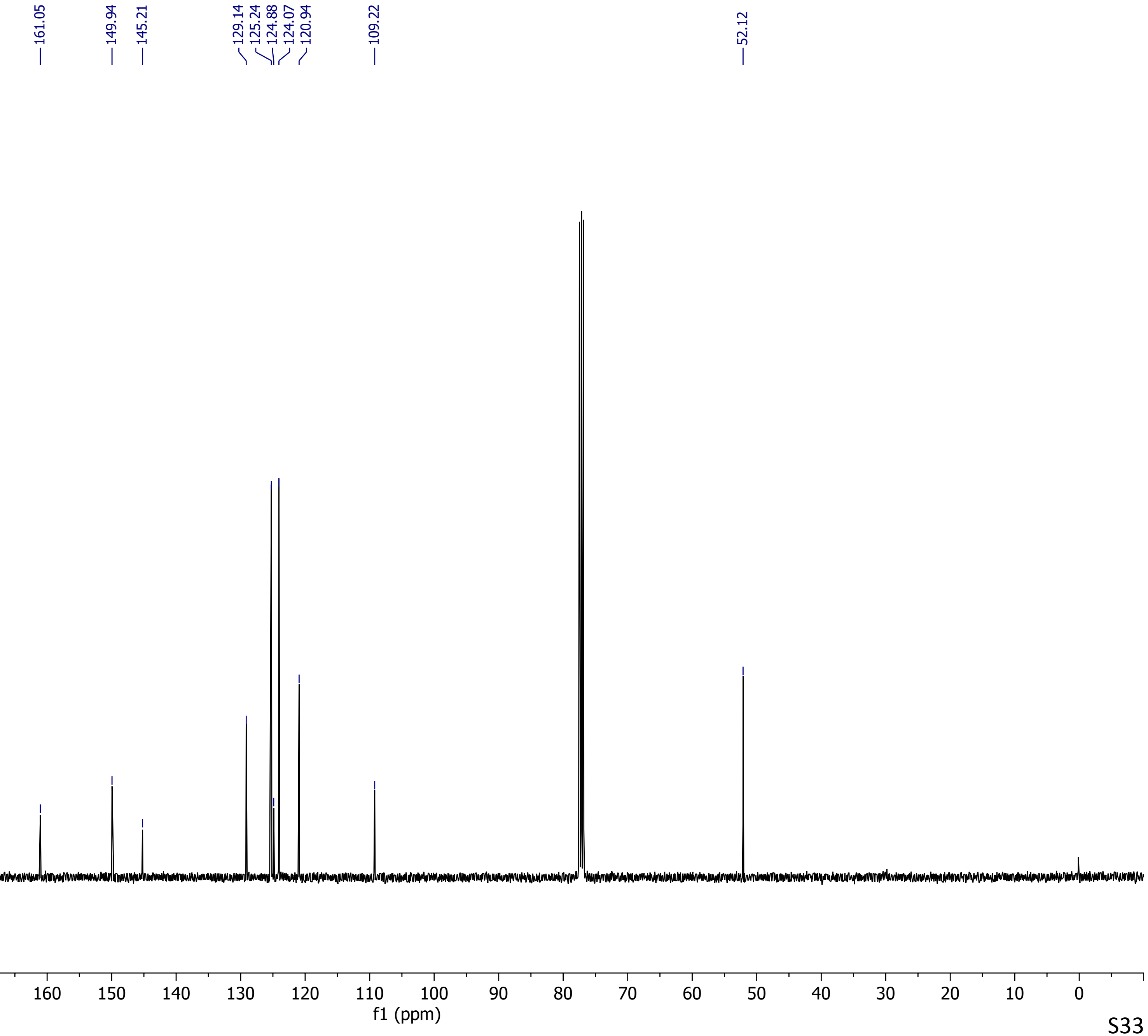

^{1}H NMR (400 MHz, CDCl$_3$)

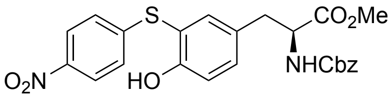

9o

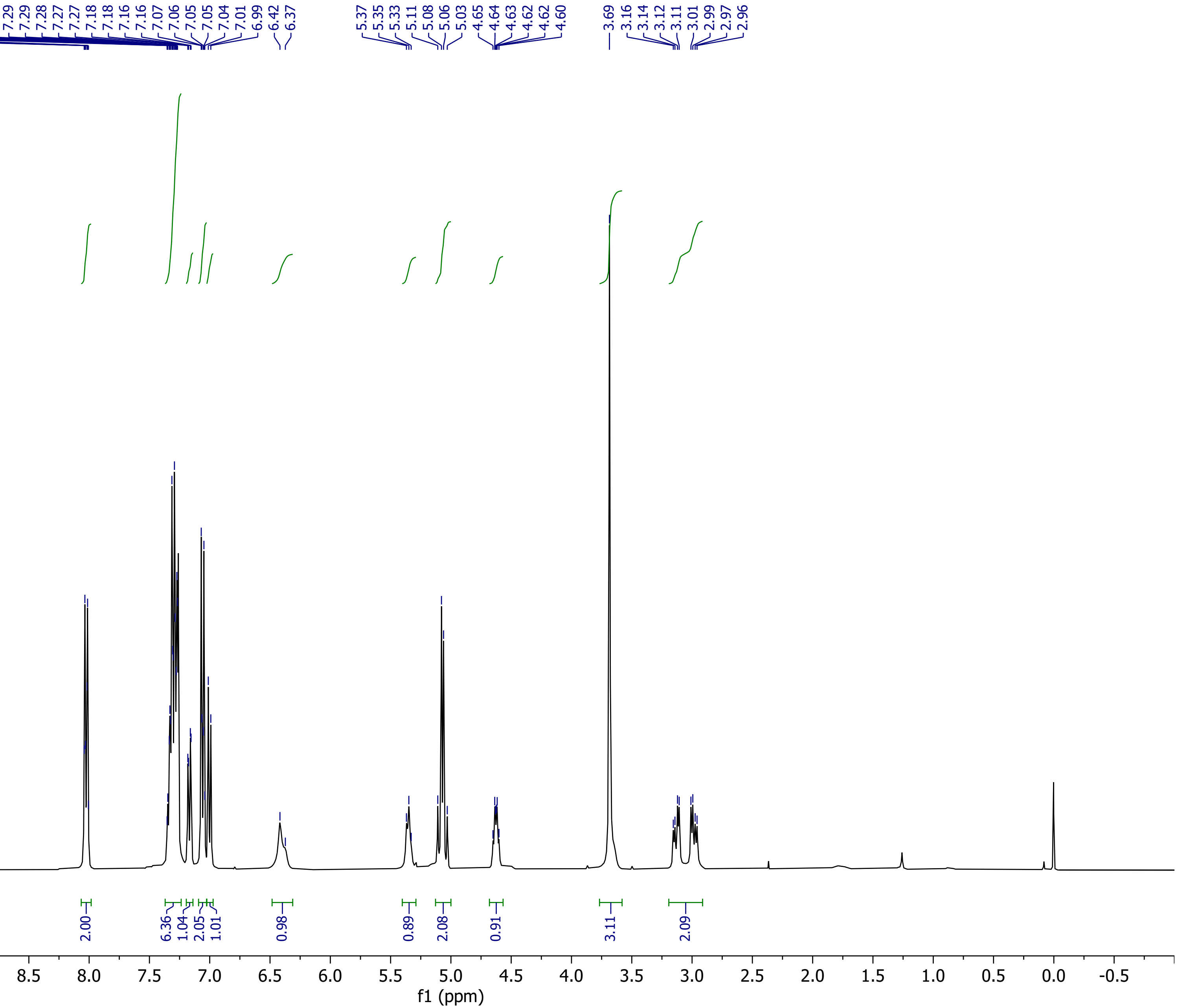

$^{13}C\{^1H\}$ NMR (101 MHz, $CDCl_3$)

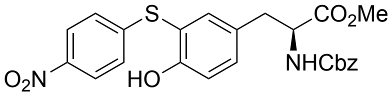

9o

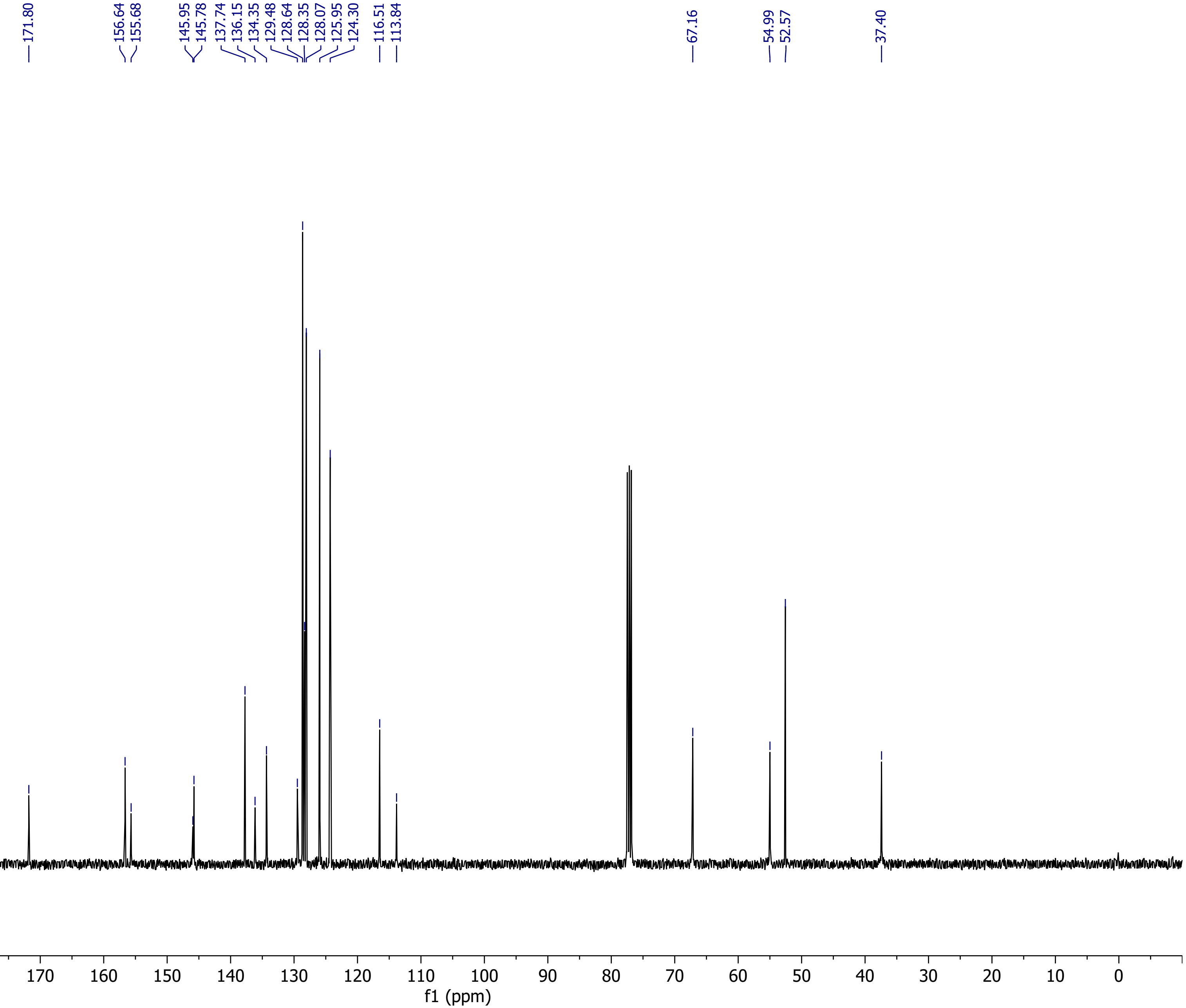

^1H NMR (400 MHz, $CDCl_3$)

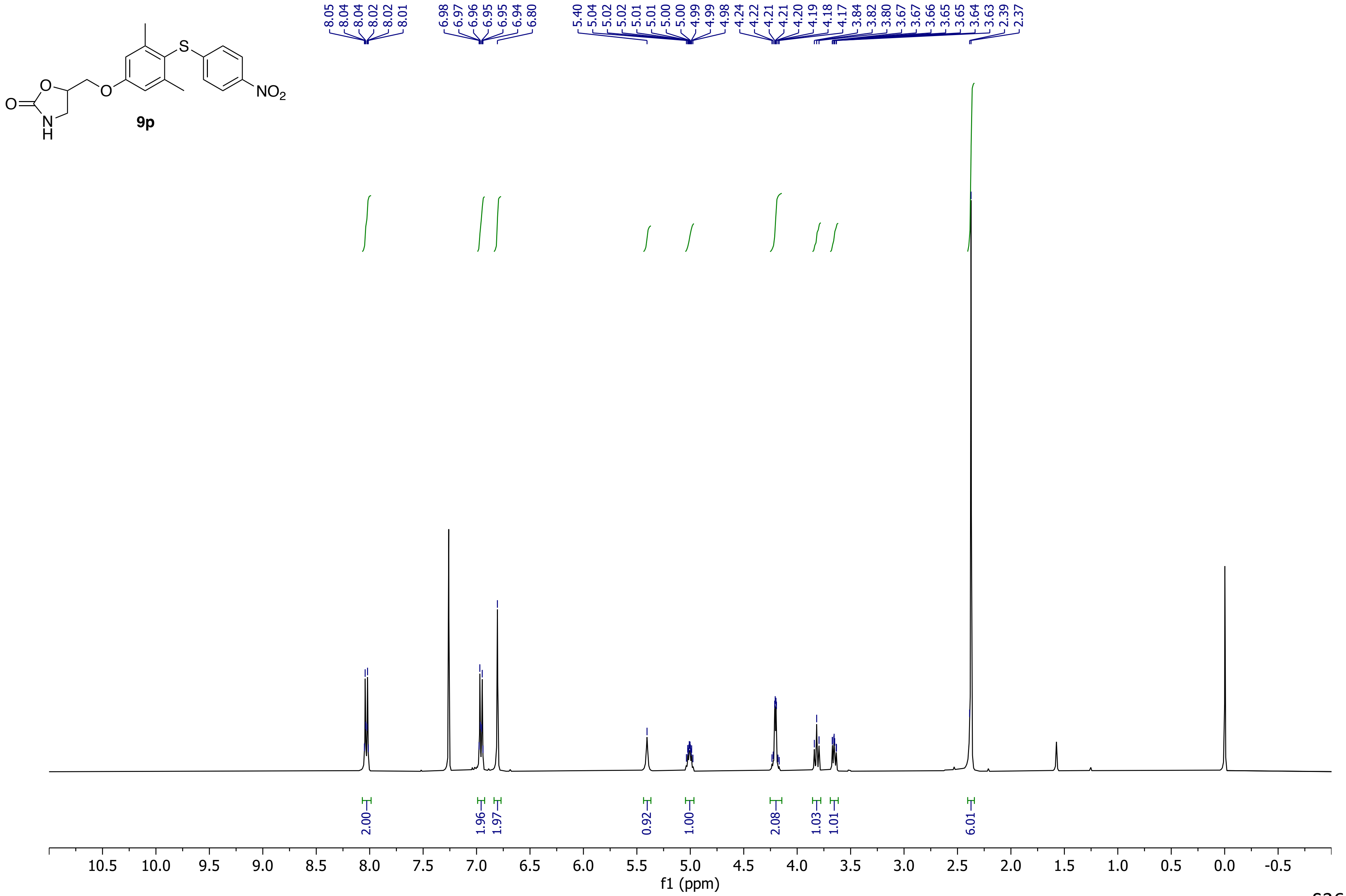

$^{13}C\{^{1}H\}$ NMR (101 MHz, $CDCl_3$)

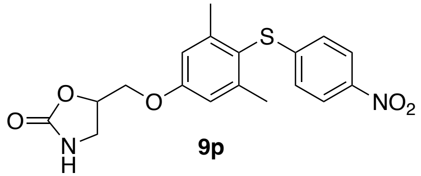

9p

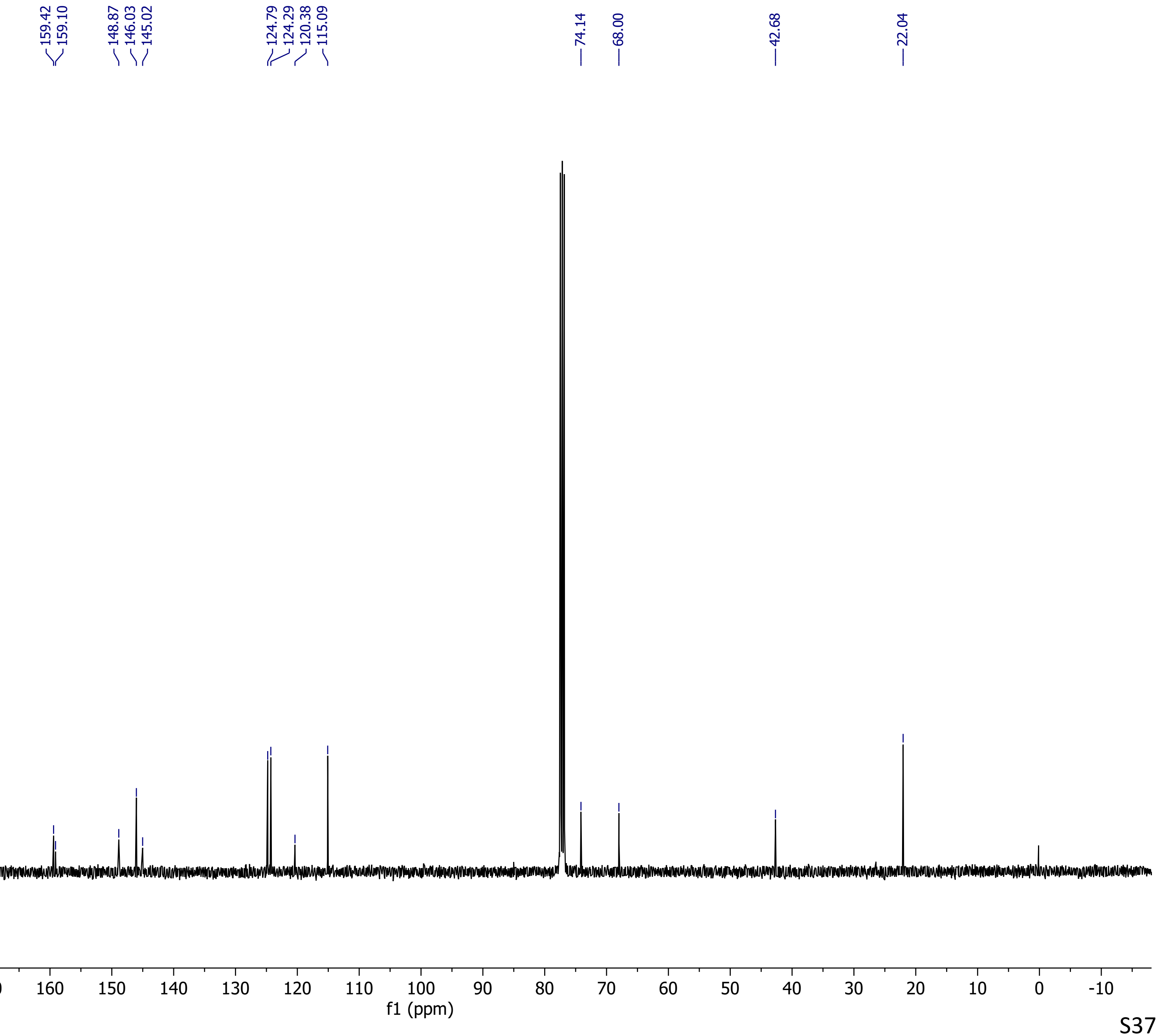

^{1}H NMR (400 MHz, $CDCl_3$)

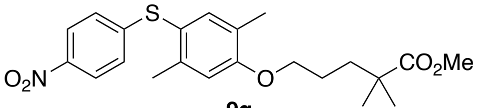

9q

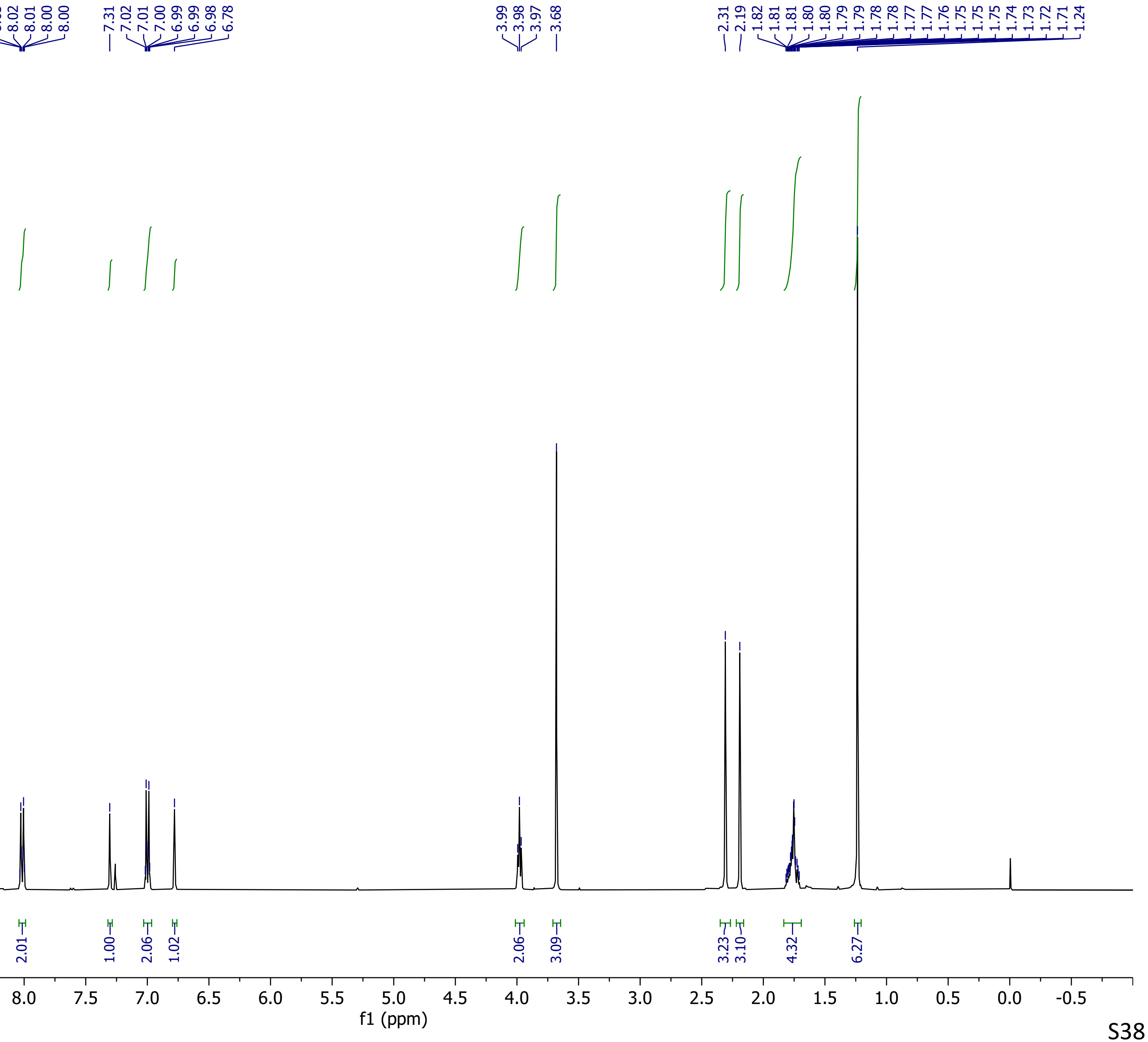

$^{13}C\{^1H\}$ NMR (101 MHz, $CDCl_3$)

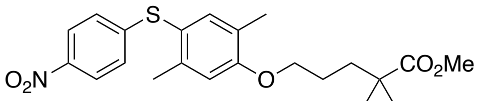

9q

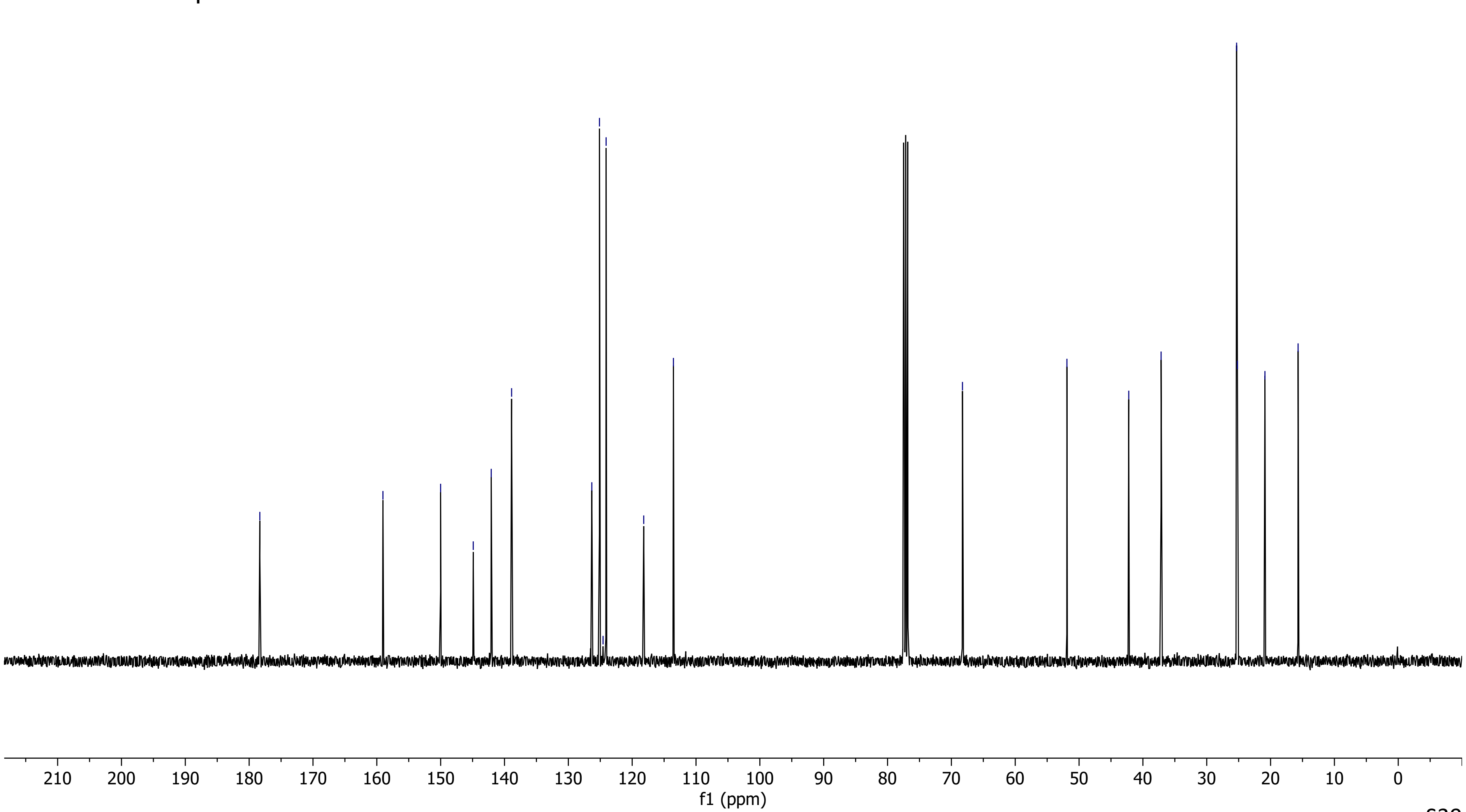

^{1}H NMR (400 MHz, $CDCl_3$)

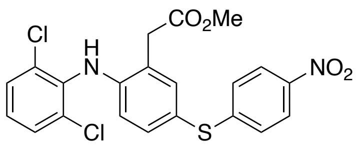

9r

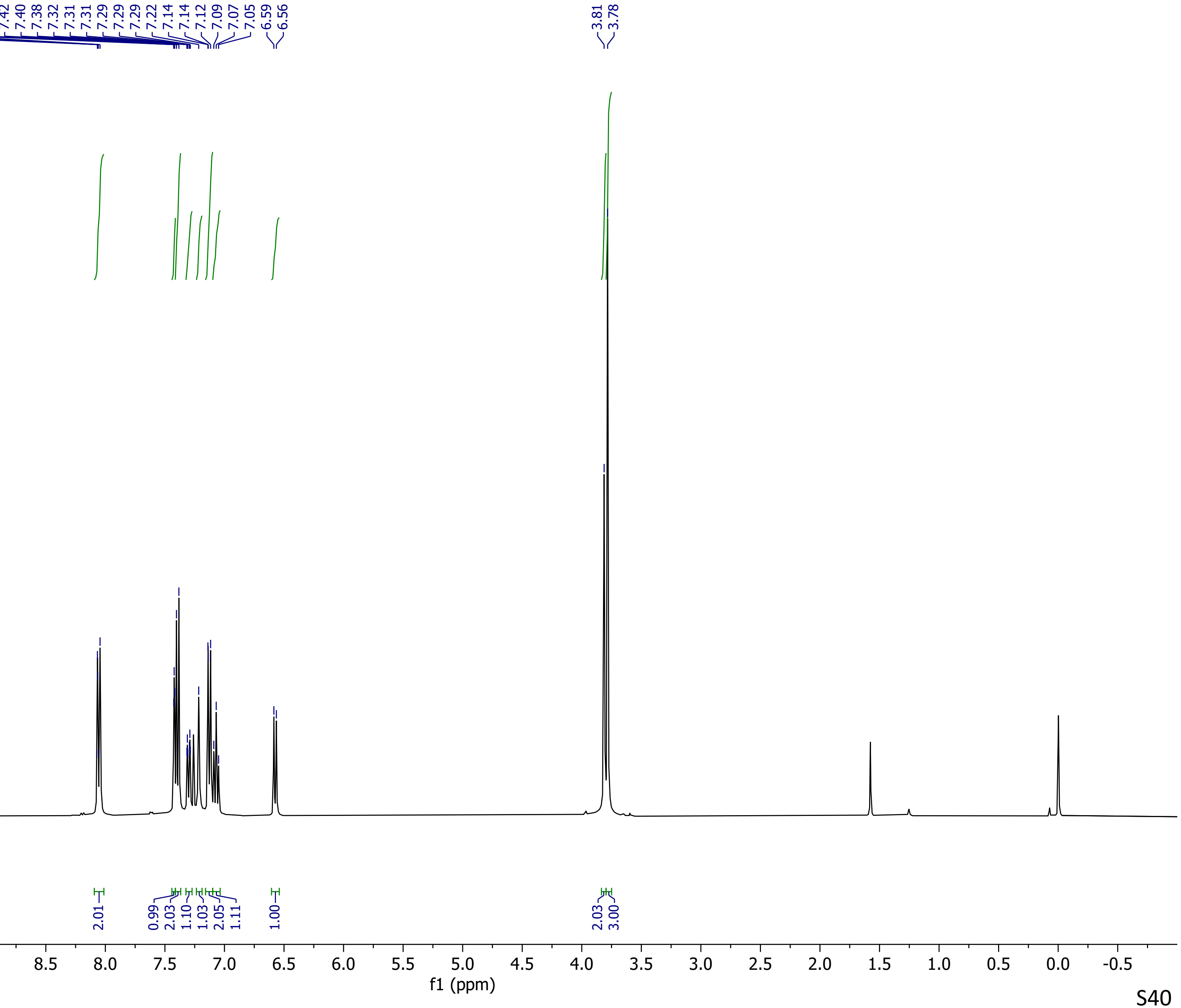

$^{13}C\{^{1}H\}$ NMR (101 MHz, $CDCl_3$)

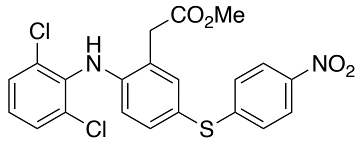

9r

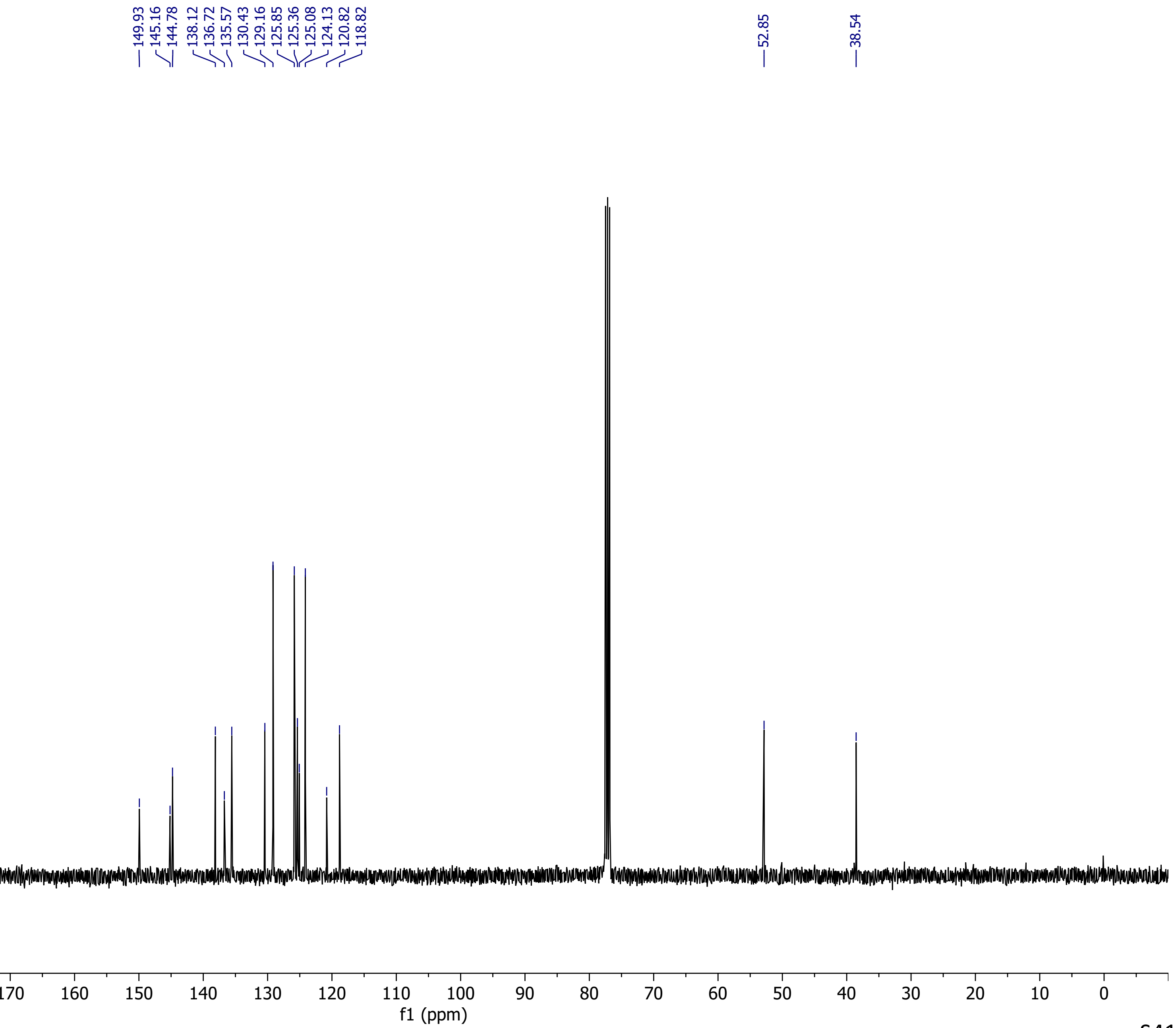

^{1}H NMR (400 MHz, $CDCl_3$)

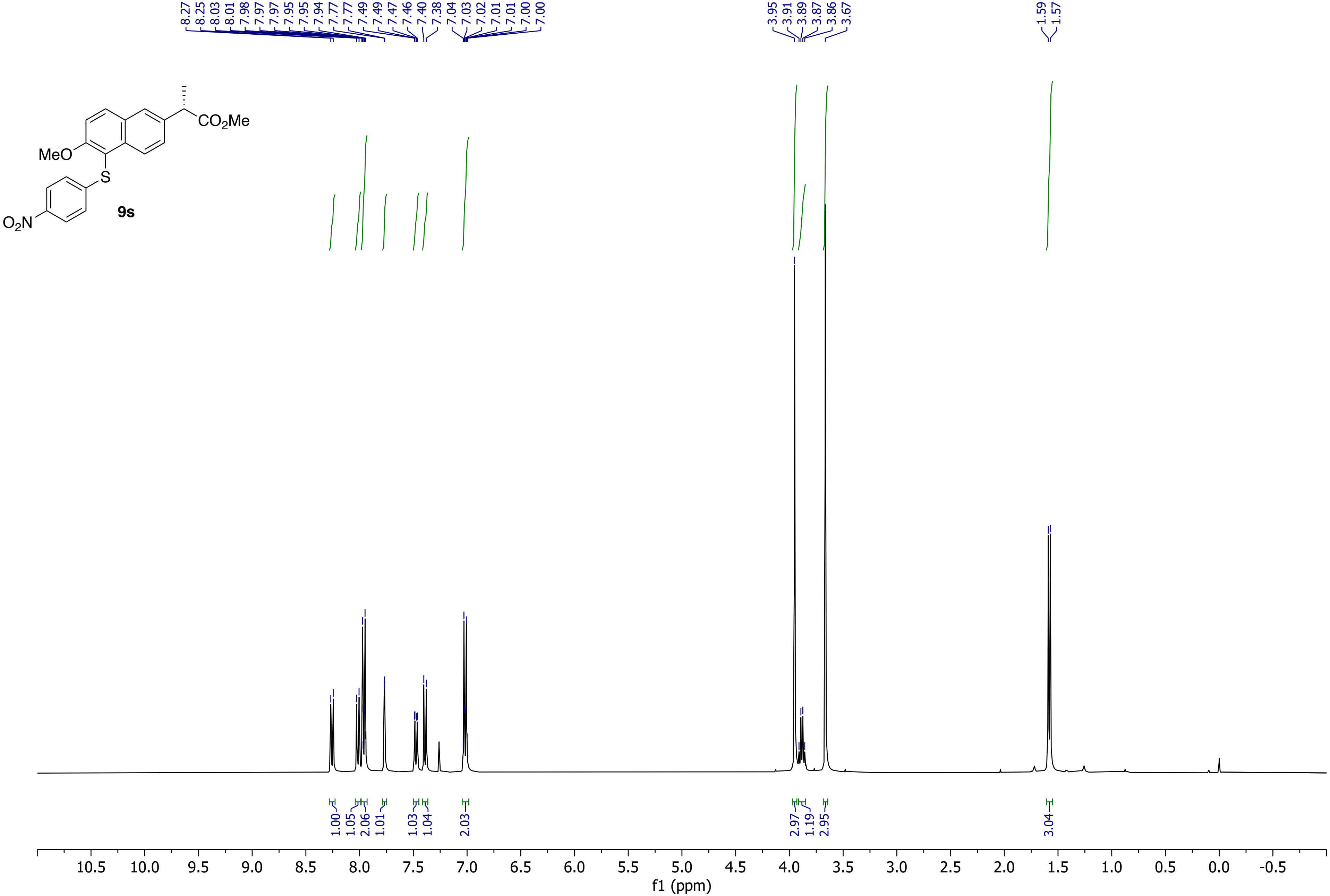

$^{13}C\{^1H\}$ NMR (101 MHz, $CDCl_3$)

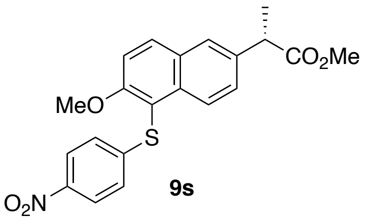

9s

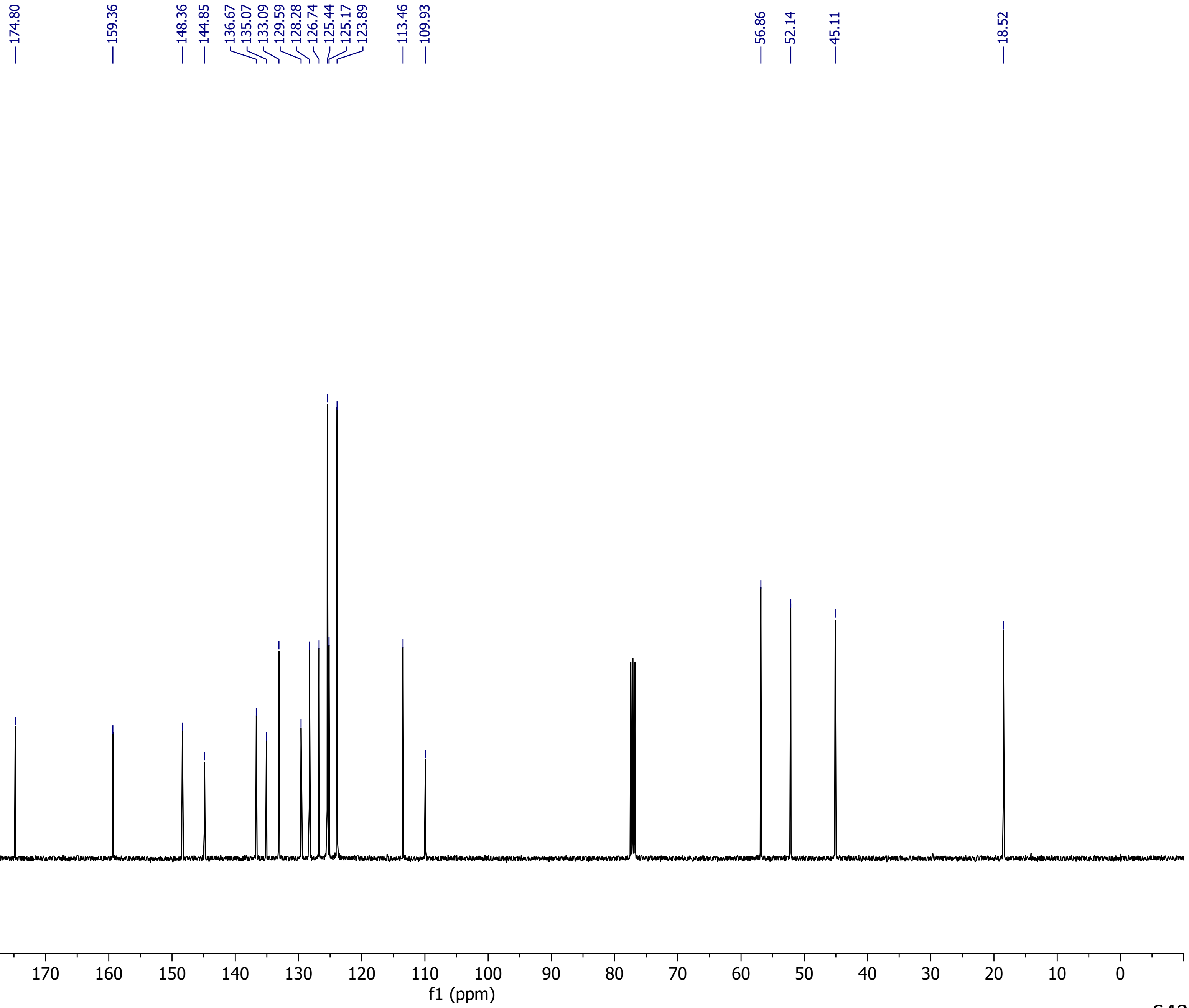

^{1}H NMR (400 MHz, $CDCl_3$)

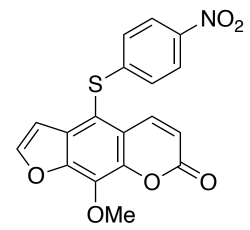

9t

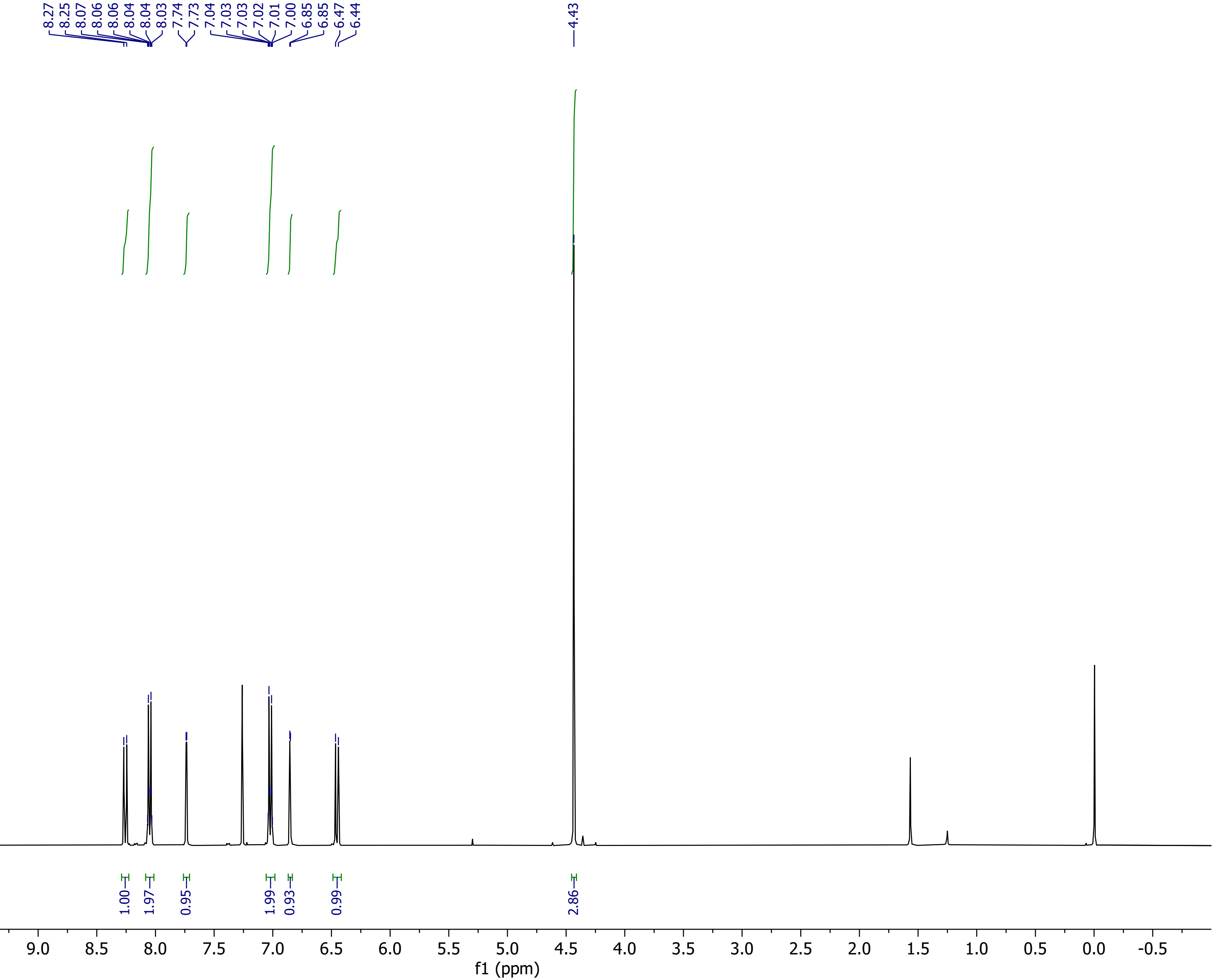

$^{13}C\{^{1}H\}$ NMR (101 MHz, $CDCl_3$)

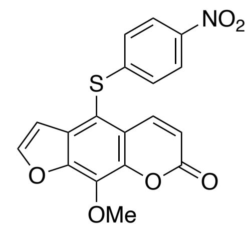

9t

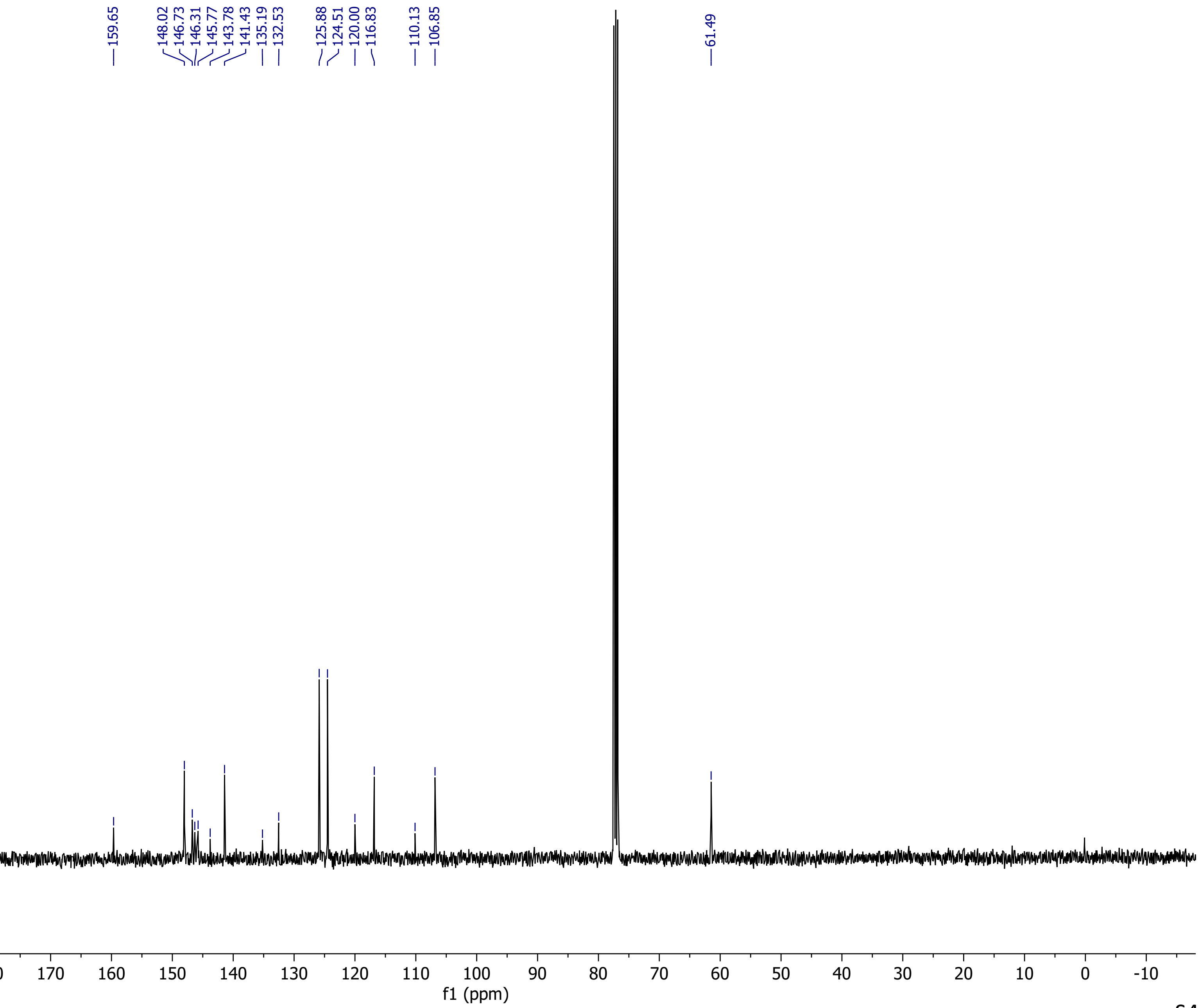

^{1}H NMR (400 MHz, DMSO-d_6)

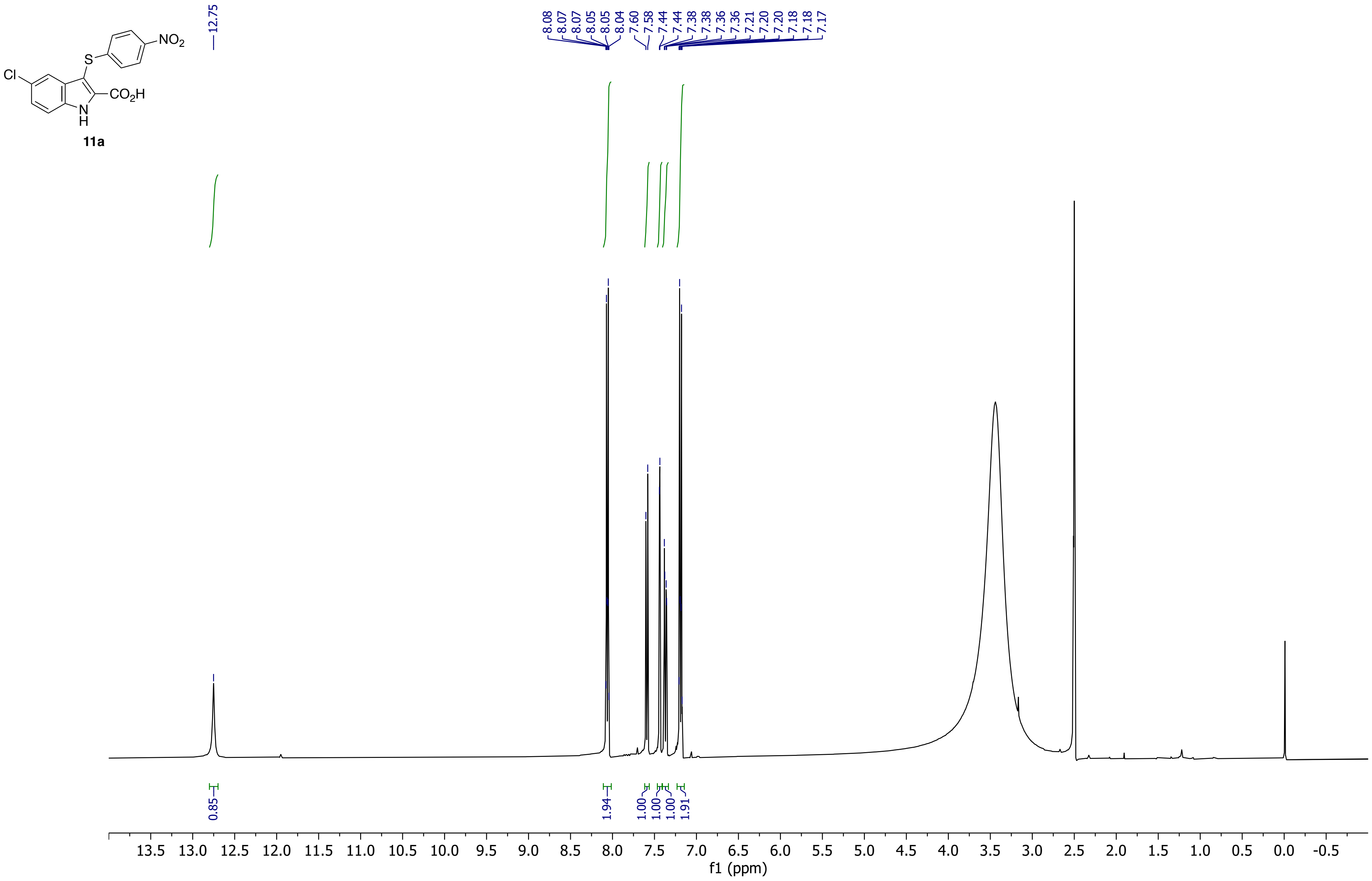

$^{13}C\{^1H\}$ NMR (101 MHz, DMSO-d_6)

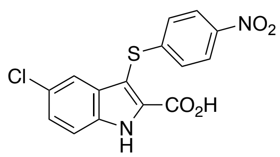

11a

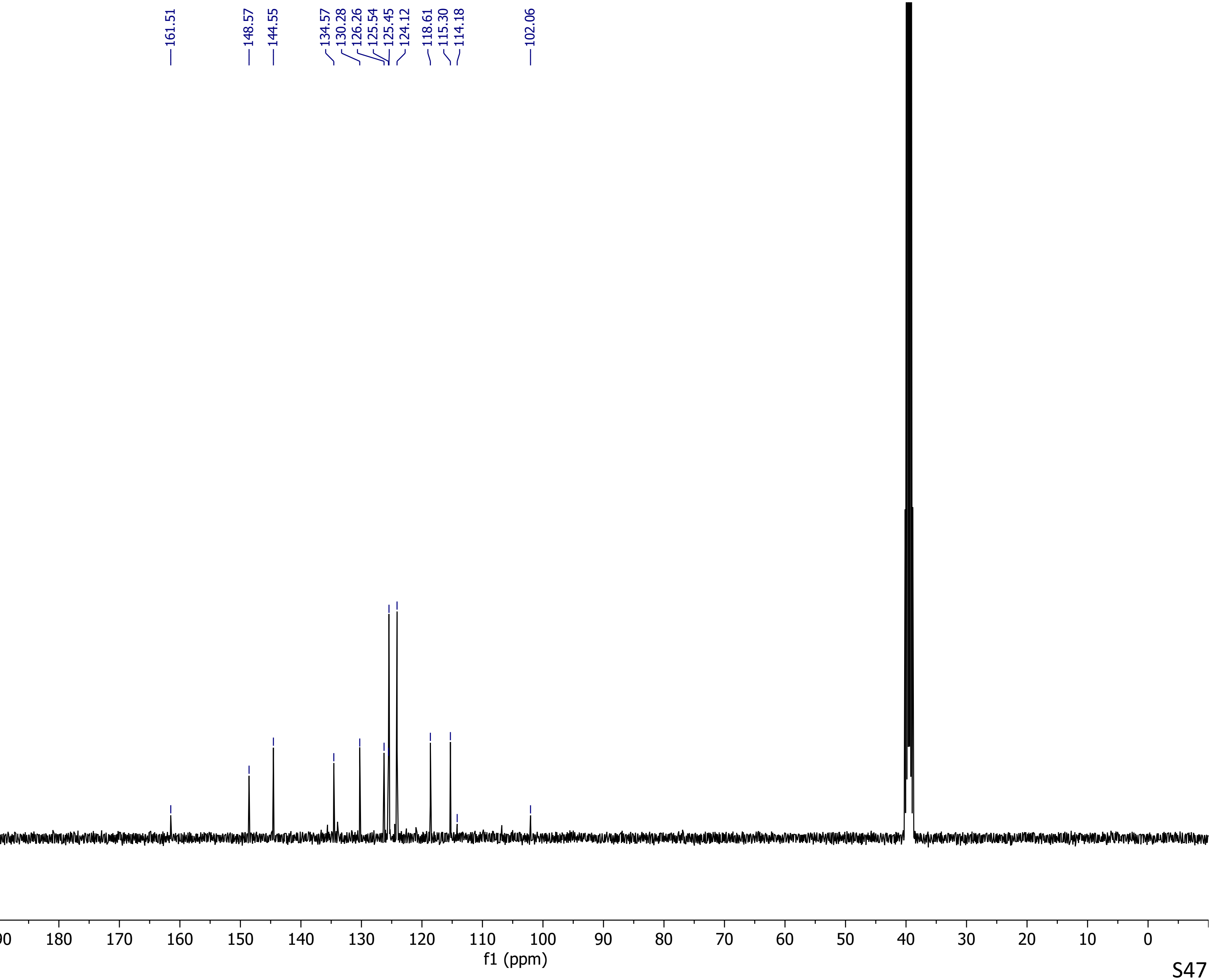

^{1}H NMR (400 MHz, DMSO-d_6)

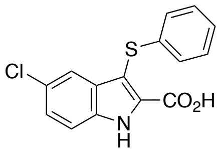

11b

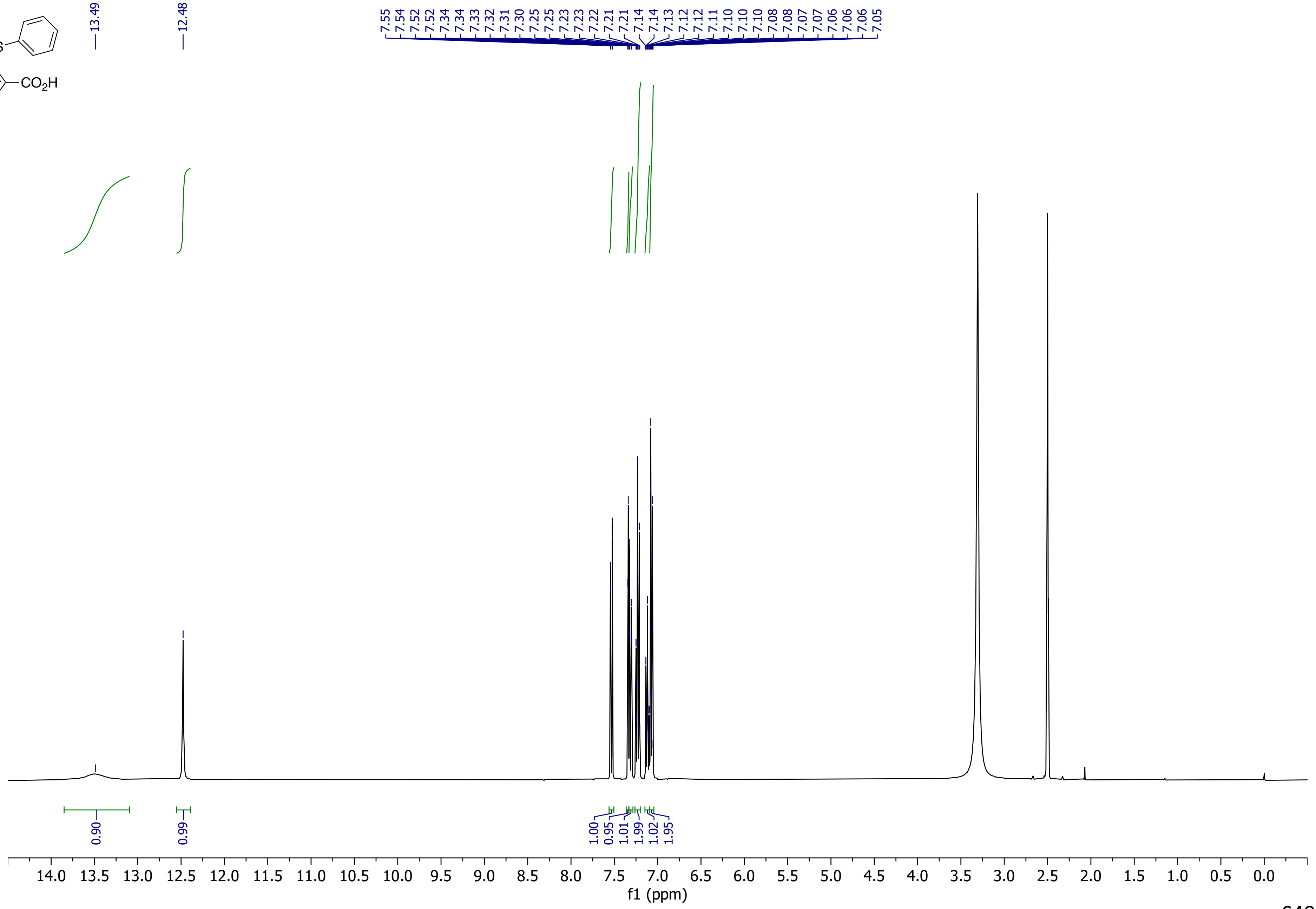

$^{13}C\{^{1}H\}$ NMR (101 MHz, DMSO-d_6)

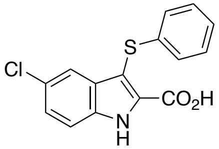

11b

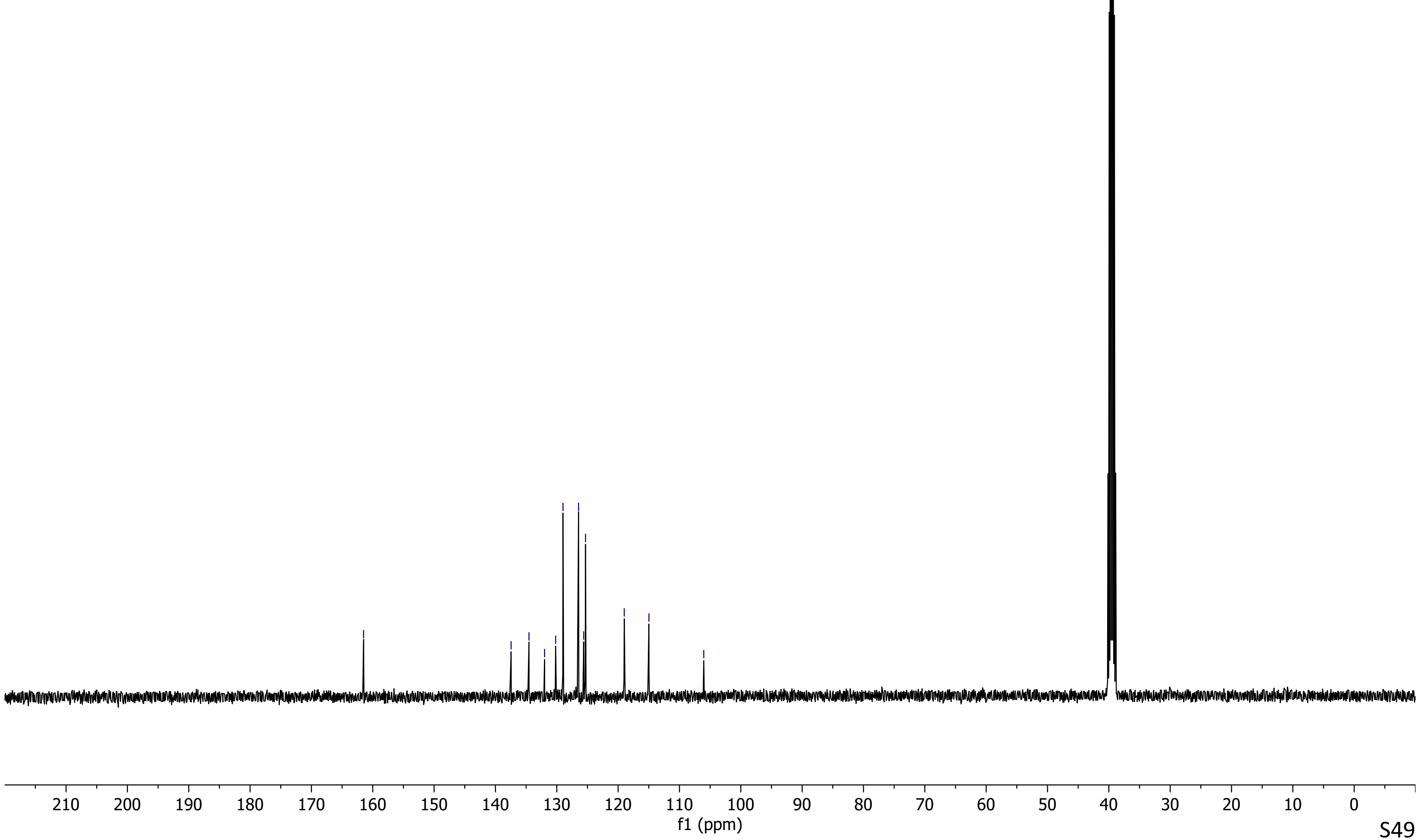

^{1}H NMR (400 MHz, DMSO-d_6)

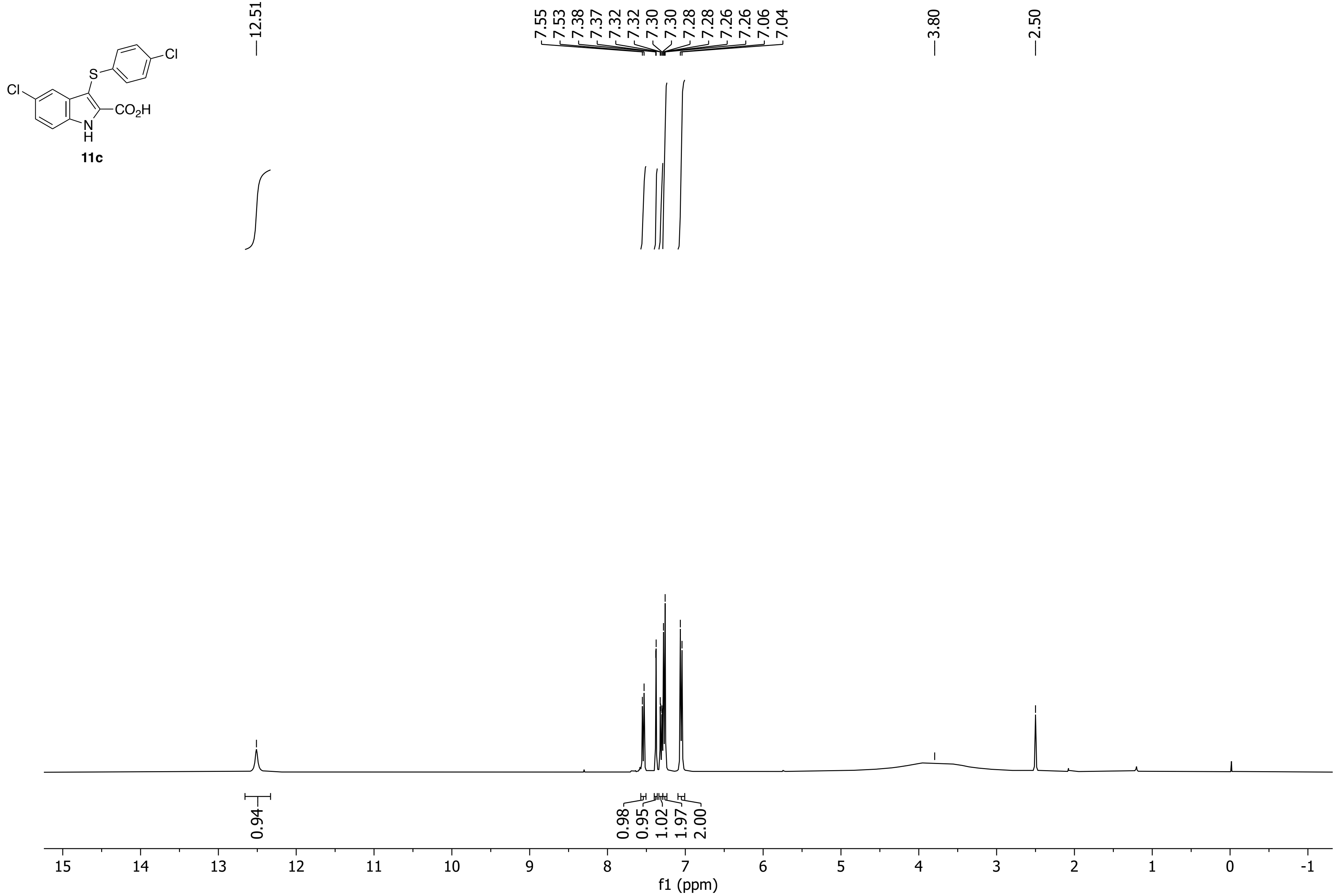

$^{13}C\{^{1}H\}$ NMR (101 MHz, DMSO-d_6)

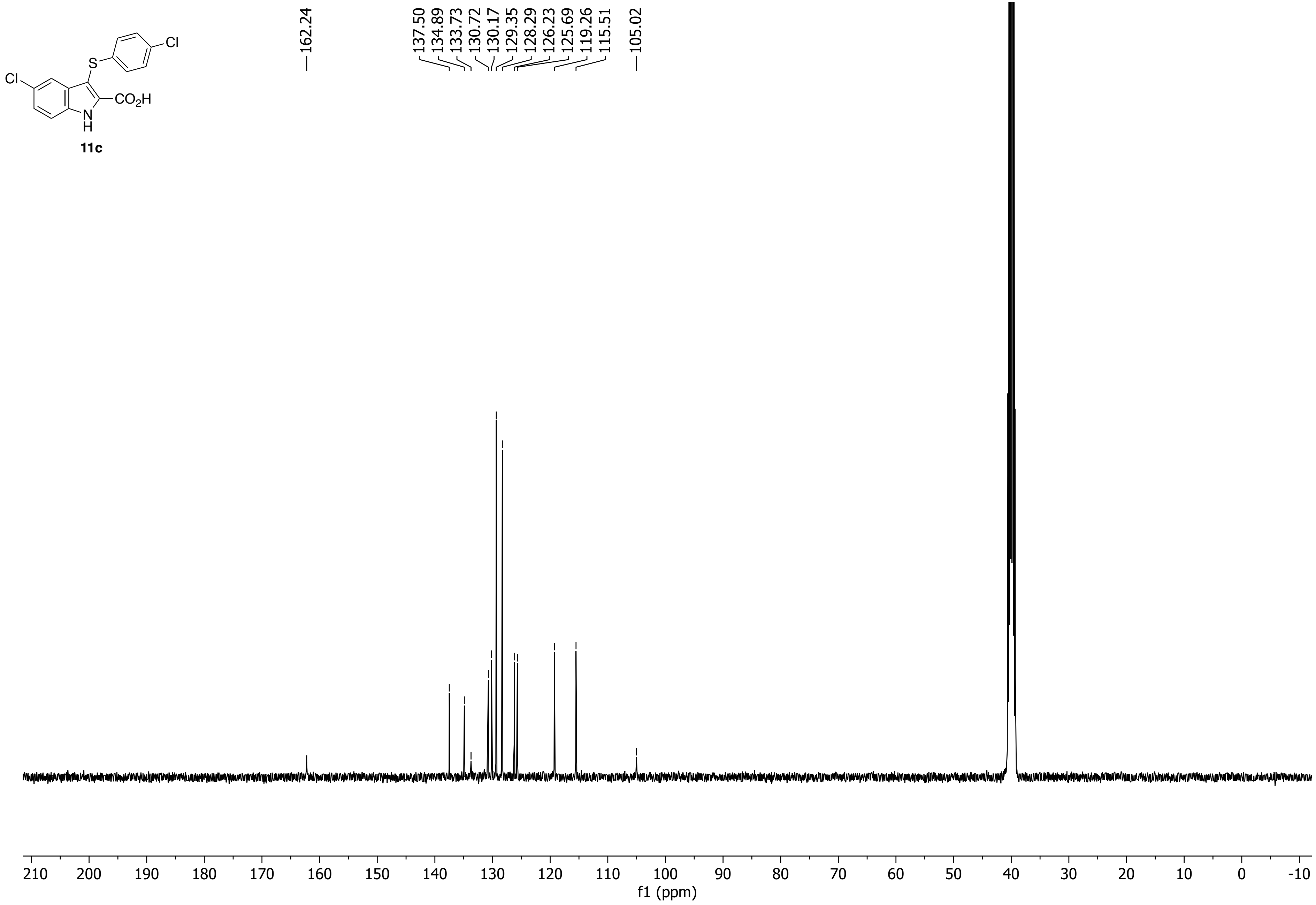

^{1}H NMR (600 MHz, DMSO-d_6)

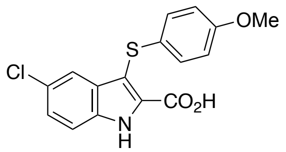

11d

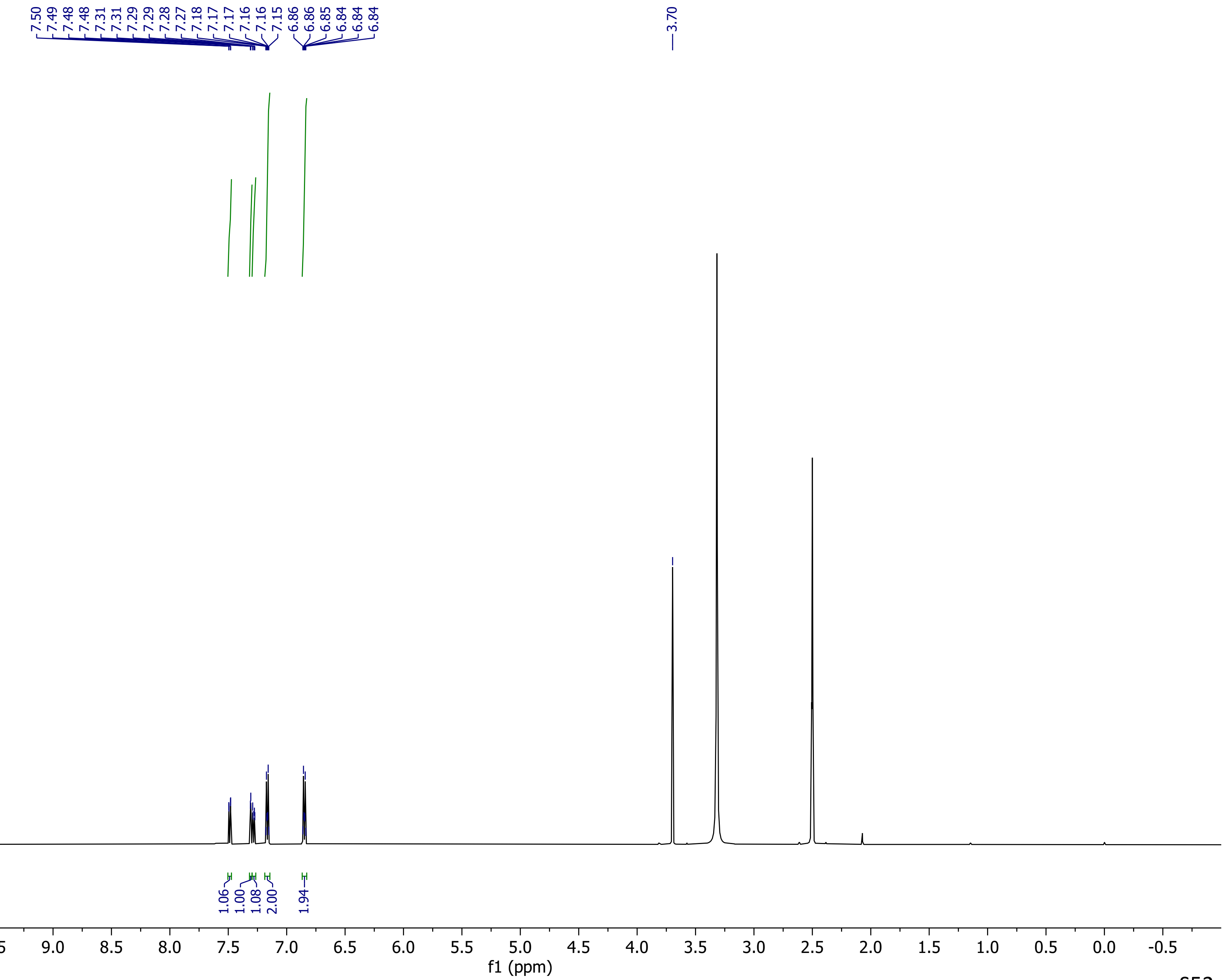

$^{13}C\{^{1}H\}$ NMR (151 MHz, DMSO-d_6)

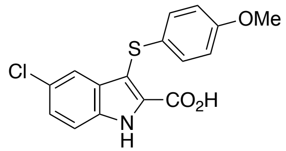

11d

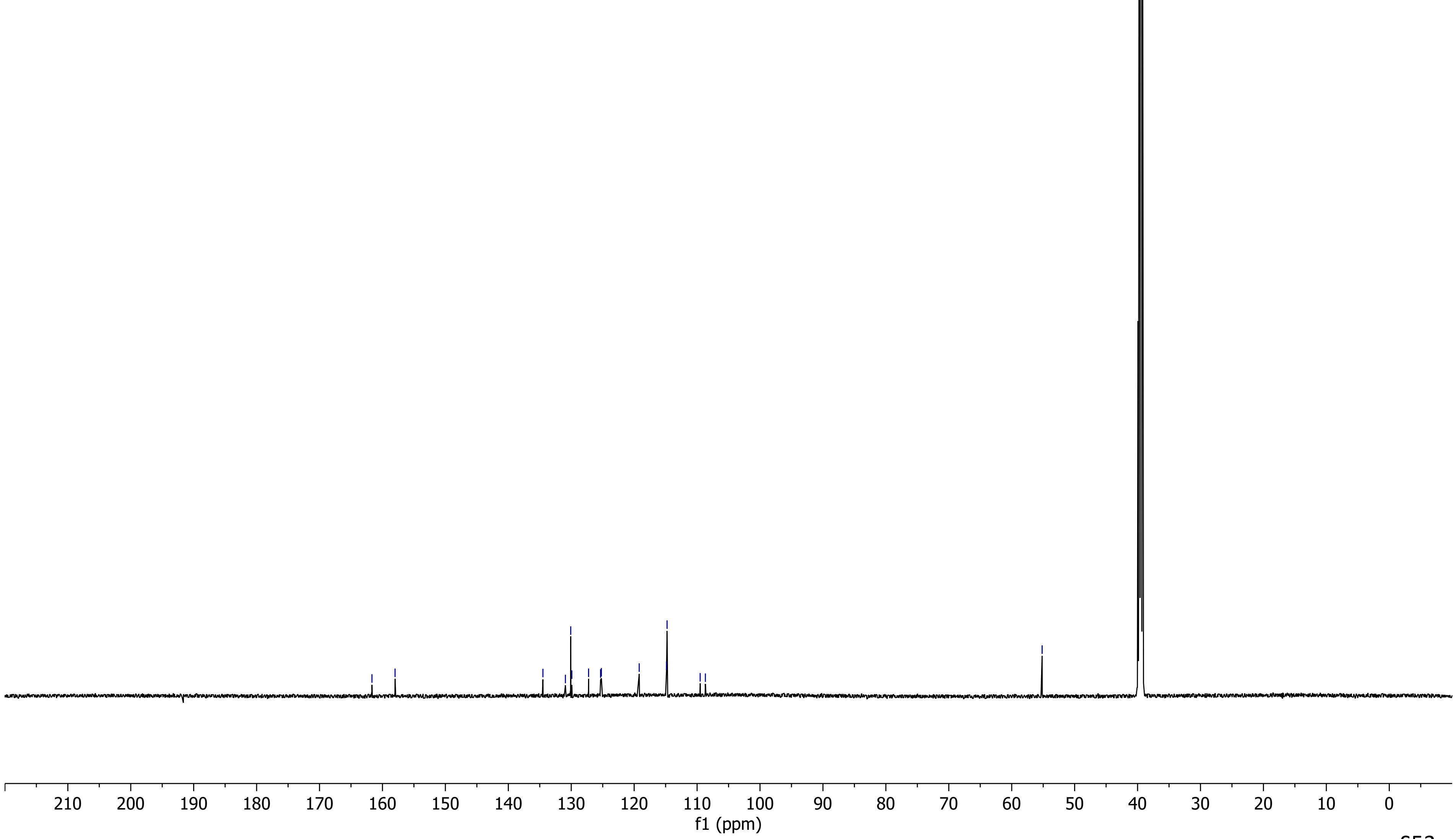

^{1}H NMR (600 MHz, $CDCl_3$)

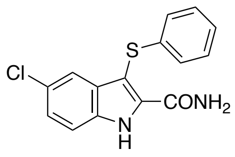

12

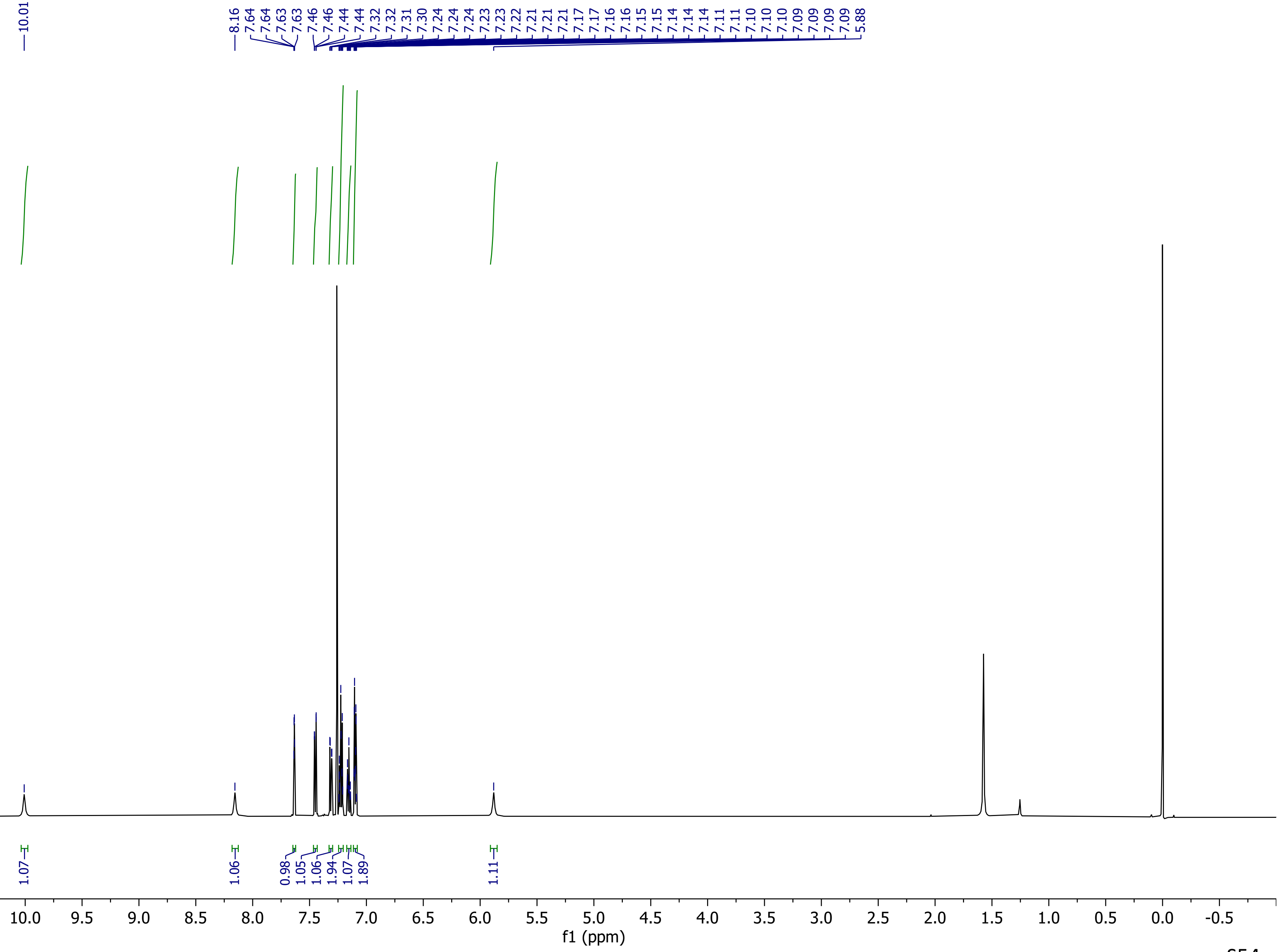

$^{13}C\{^{1}H\}$ **NMR (151 MHz, $CDCl_3$)**

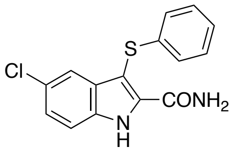

12

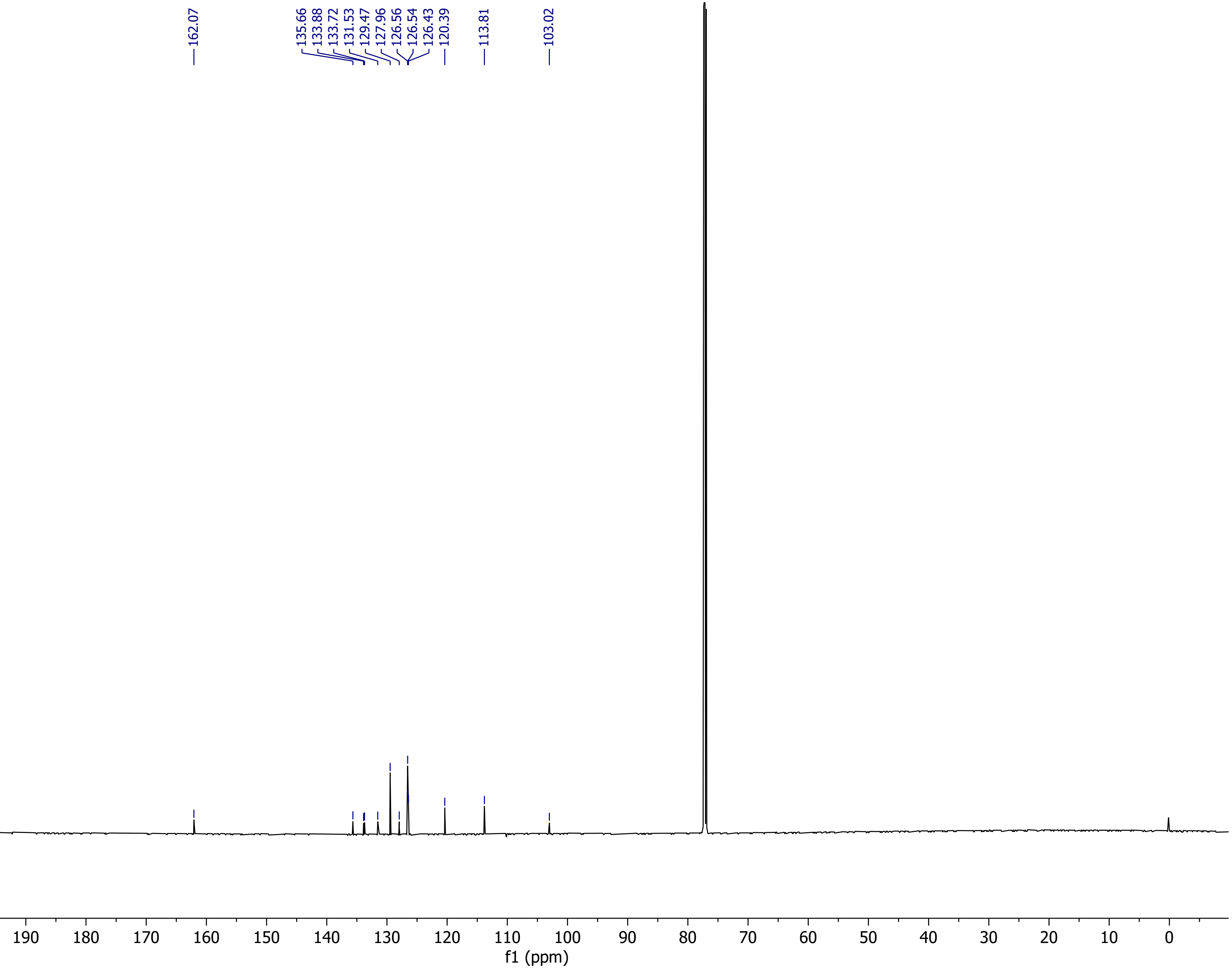

^{1}H NMR (400 MHz, DMSO-d_6)

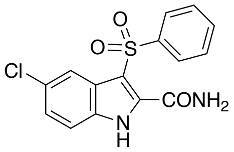

6

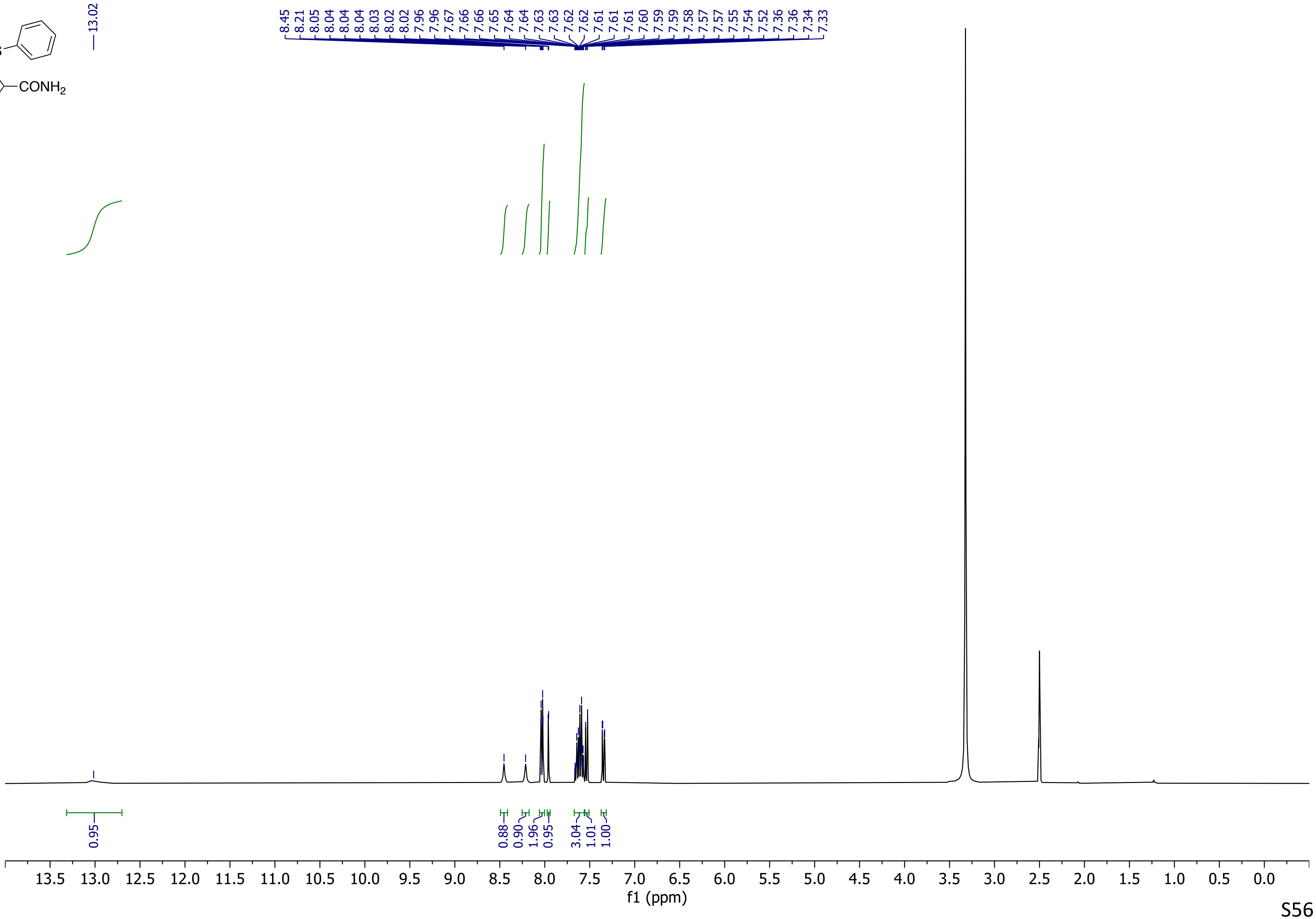

$^{13}C\{^{1}H\}$ NMR (101 MHz, DMSO-d_6)

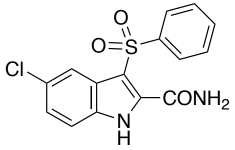

6

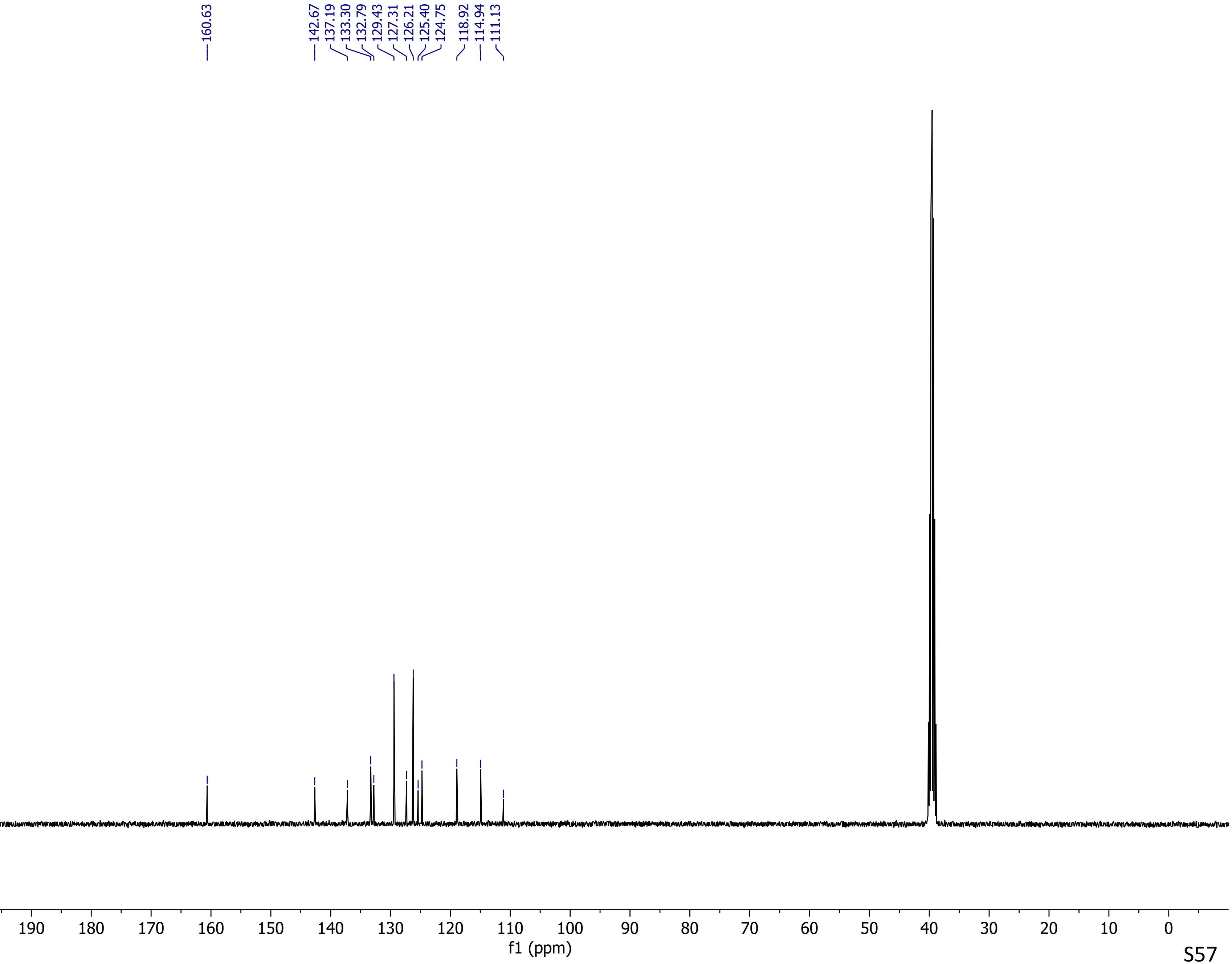

^{1}H NMR (400 MHz, $CDCl_3$)

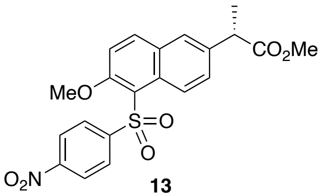

13

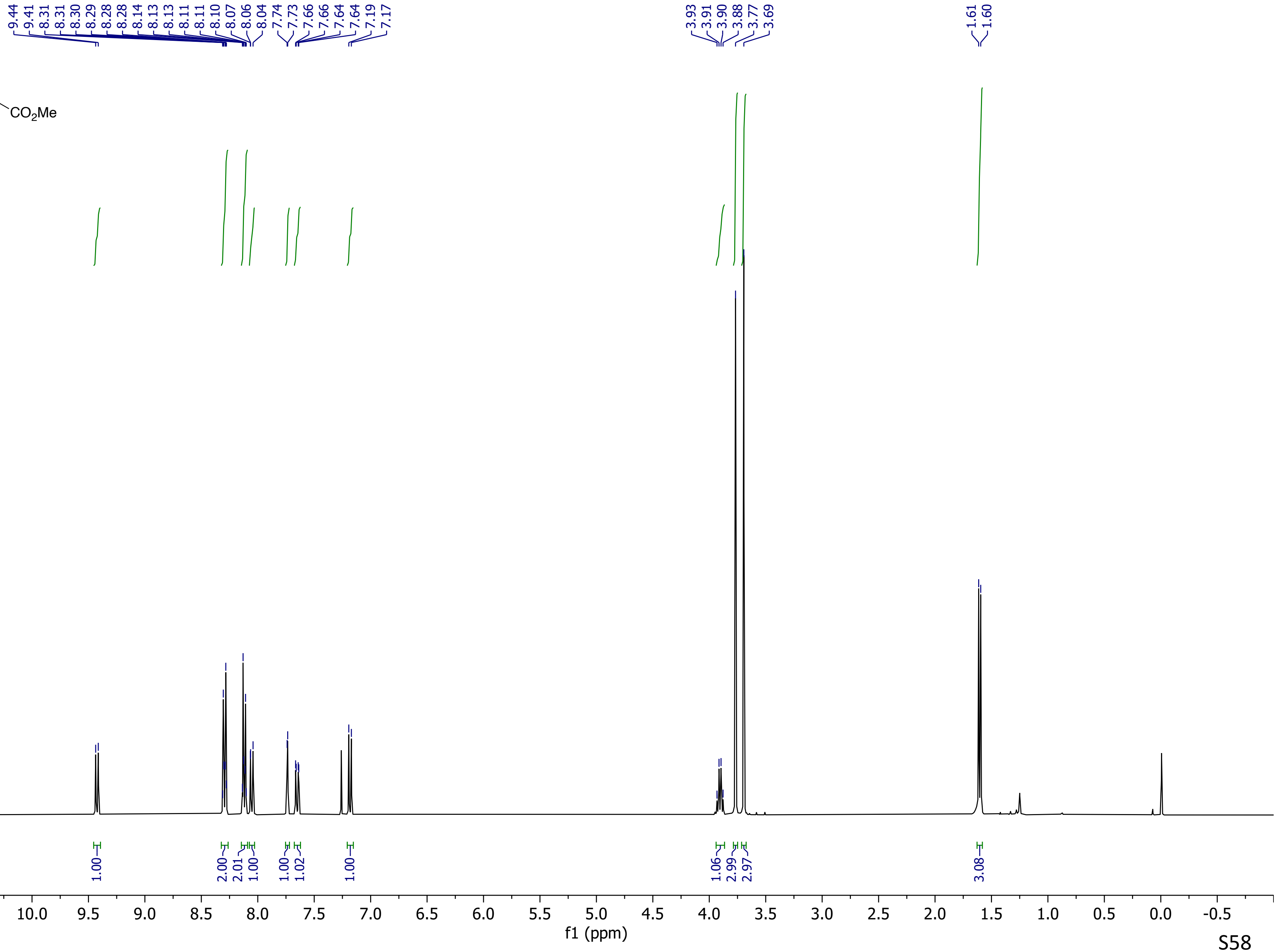

$^{13}C\{^1H\}$ NMR (101 MHz, CDCl$_3$)

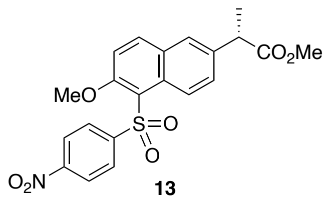
13

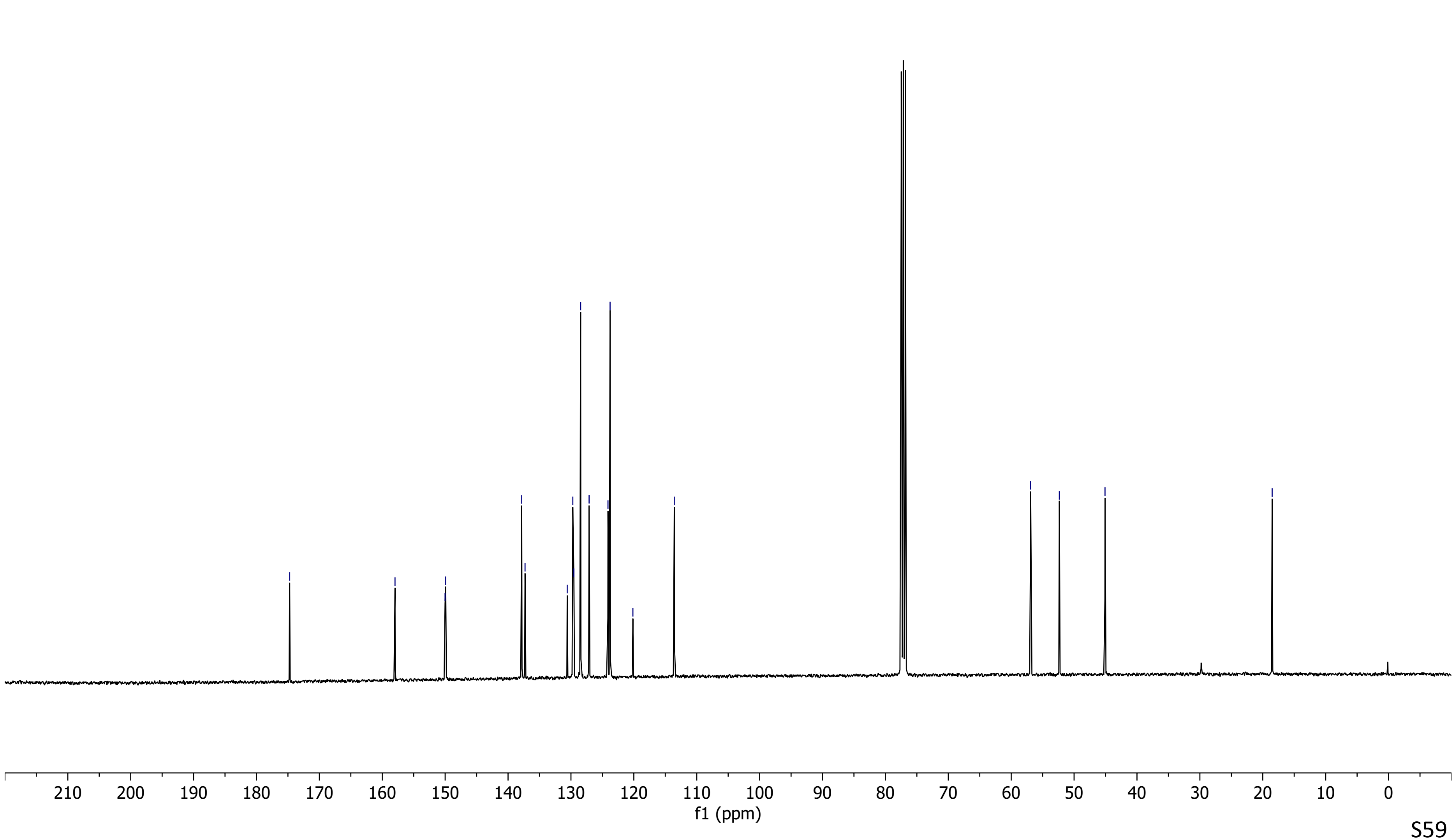

^{1}H NMR (400 MHz, $CDCl_3$)

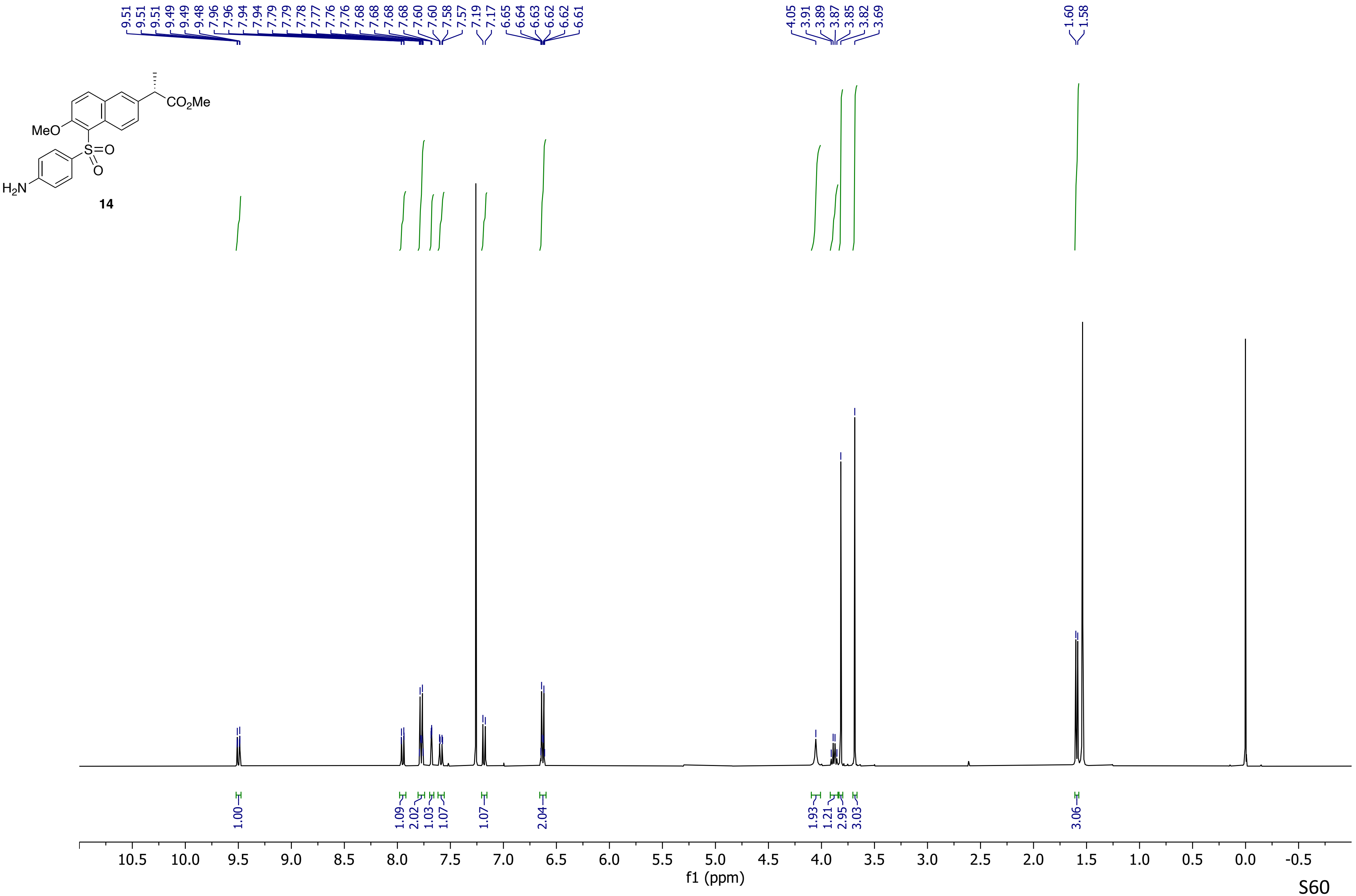

$^{13}C\{^1H\}$ NMR (101 MHz, $CDCl_3$)

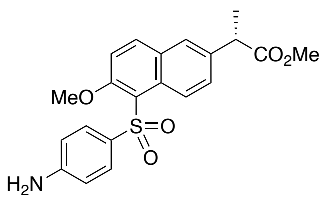

14

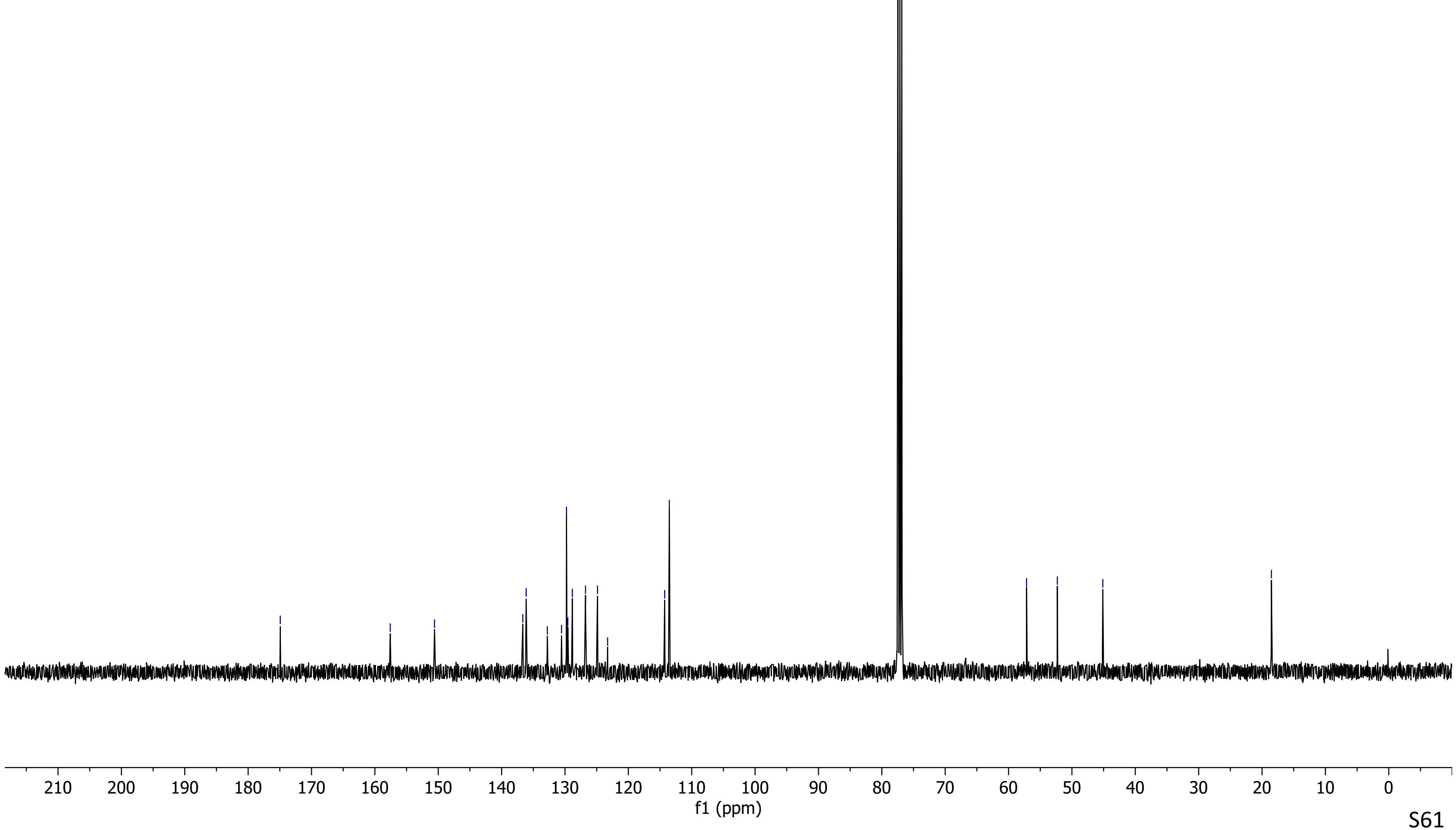

^{1}H NMR (400 MHz, DMSO-d_6)

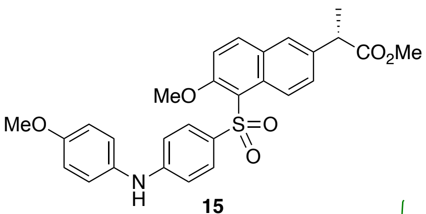

15

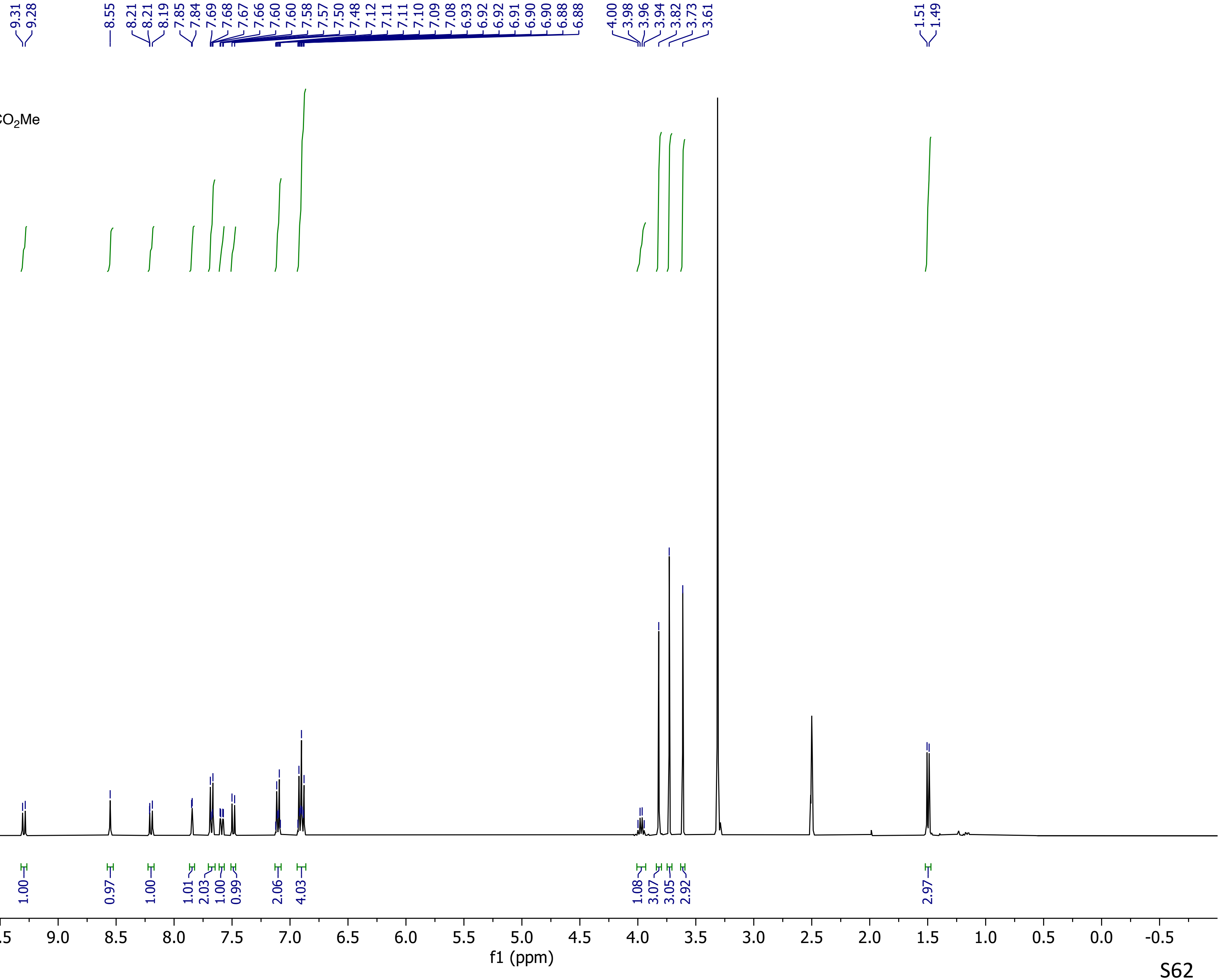

$^{13}C\{^{1}H\}$ NMR (101 MHz, DMSO-d_6)

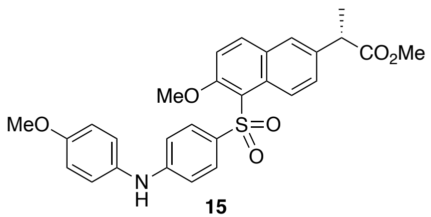

15

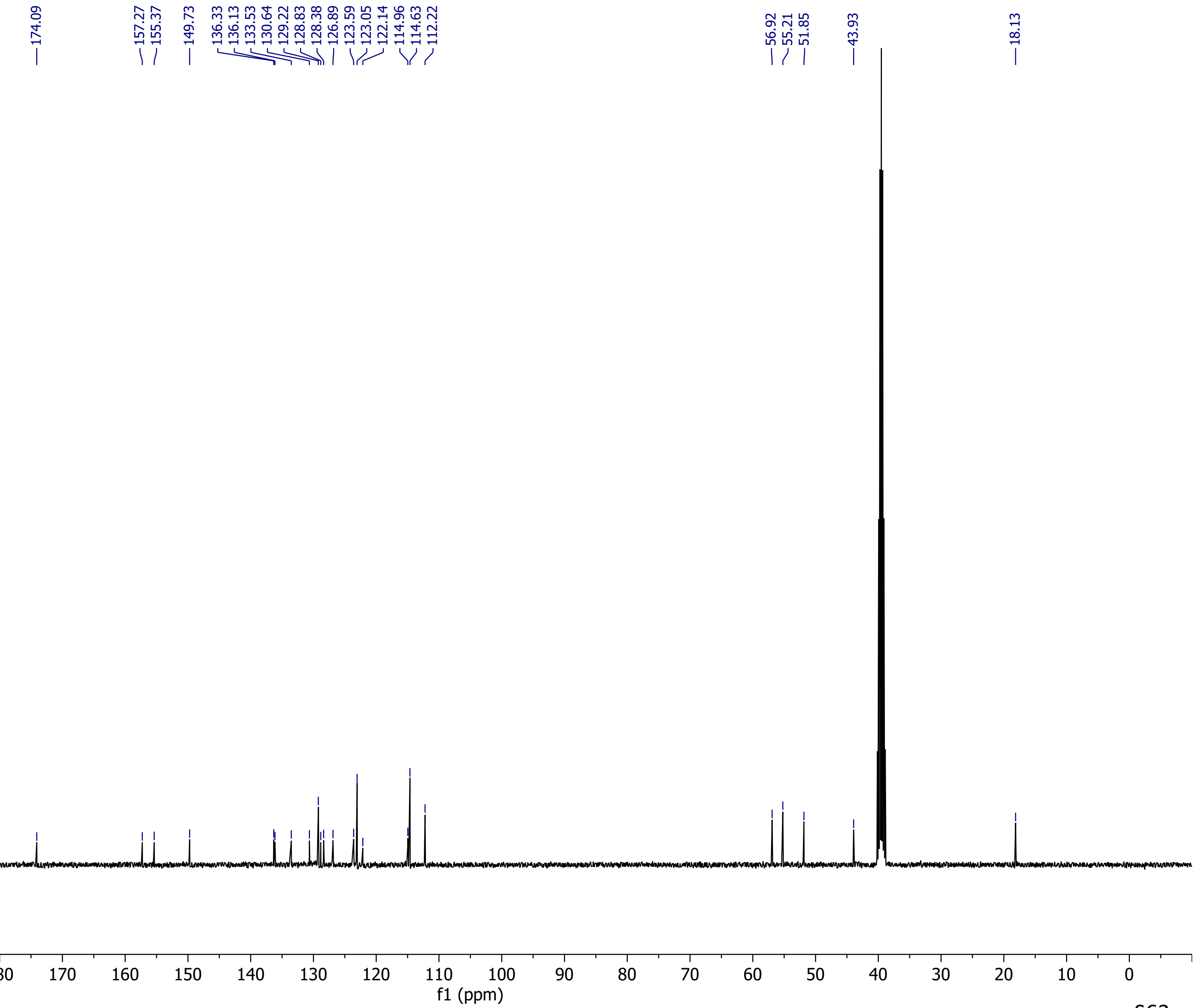

^{1}H NMR (400 MHz, $CDCl_3$)

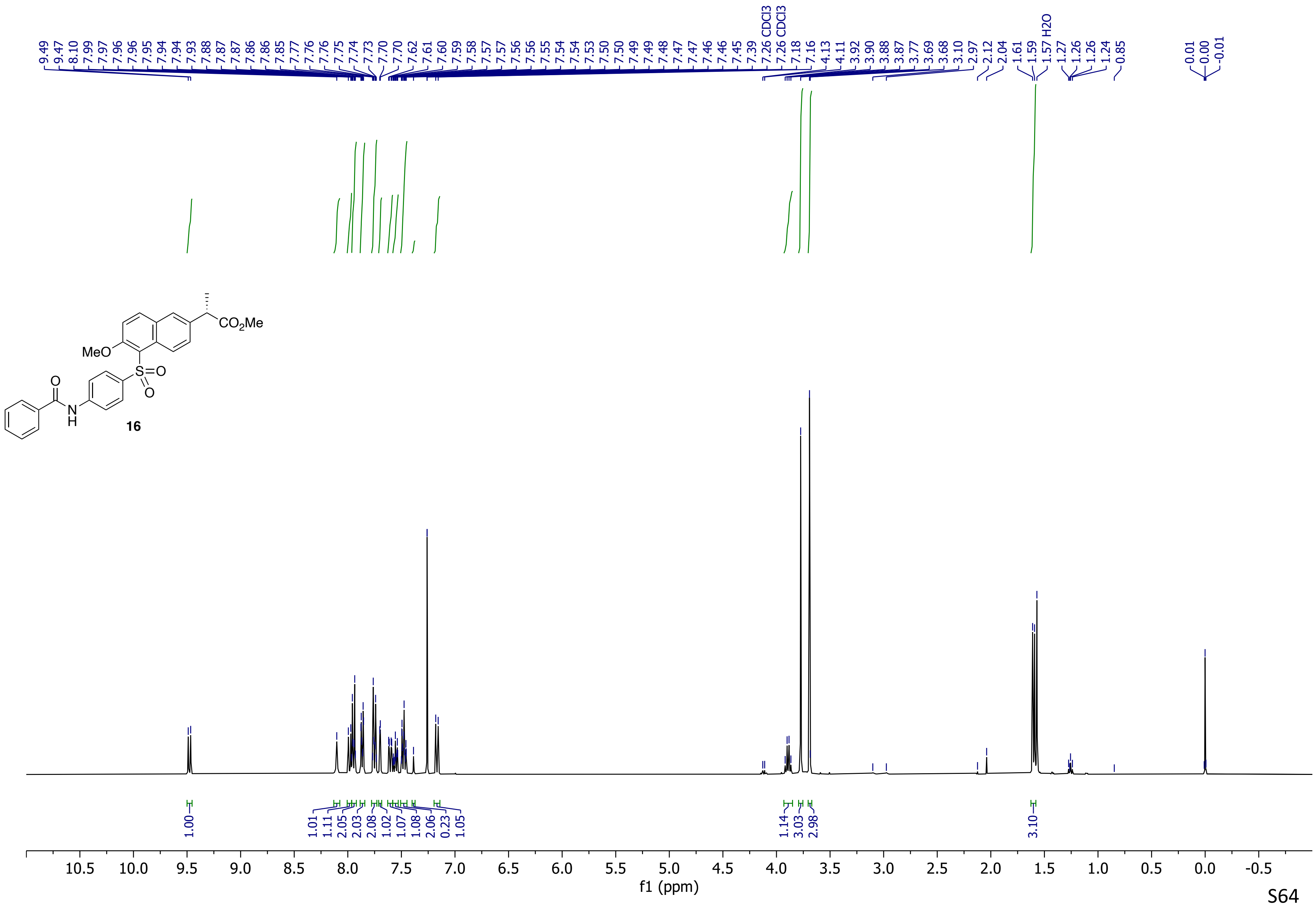
16

$^{13}C\{^1H\}$ NMR (101 MHz, $CDCl_3$)

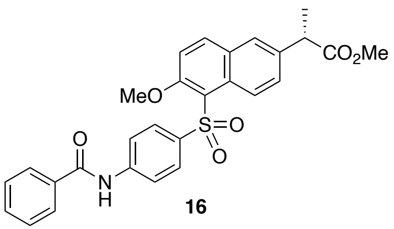

16

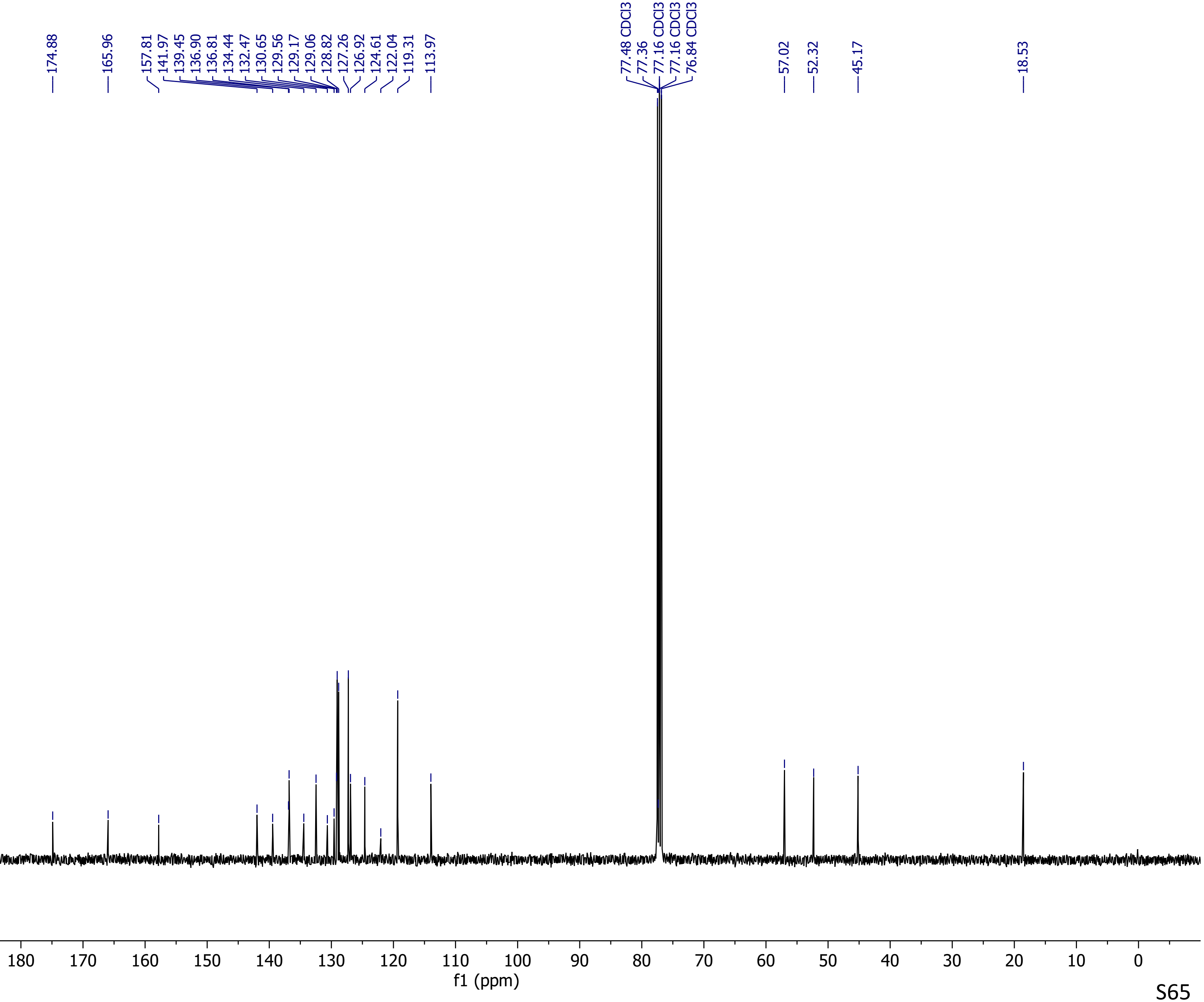

^{1}H NMR (400 MHz, $CDCl_3$)

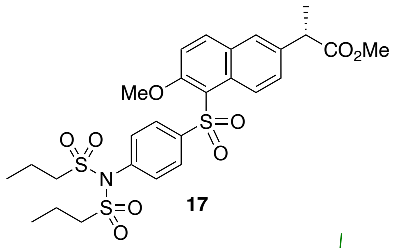

17

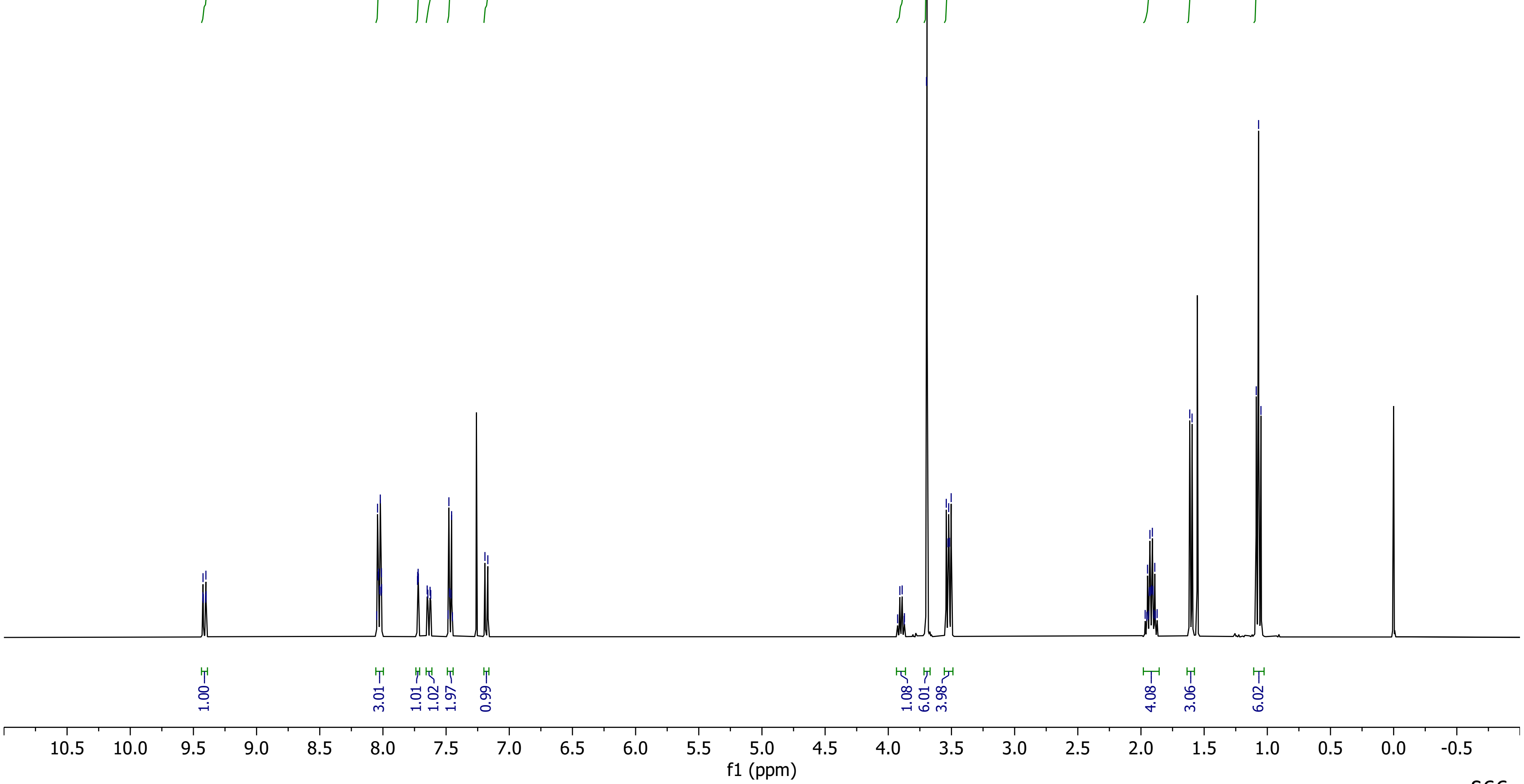

$^{13}C\{^{1}H\}$ NMR (101 MHz, $CDCl_3$)

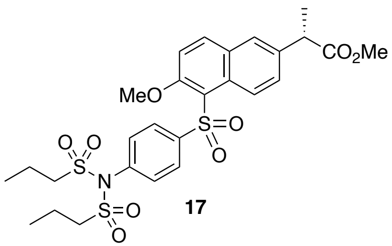

17

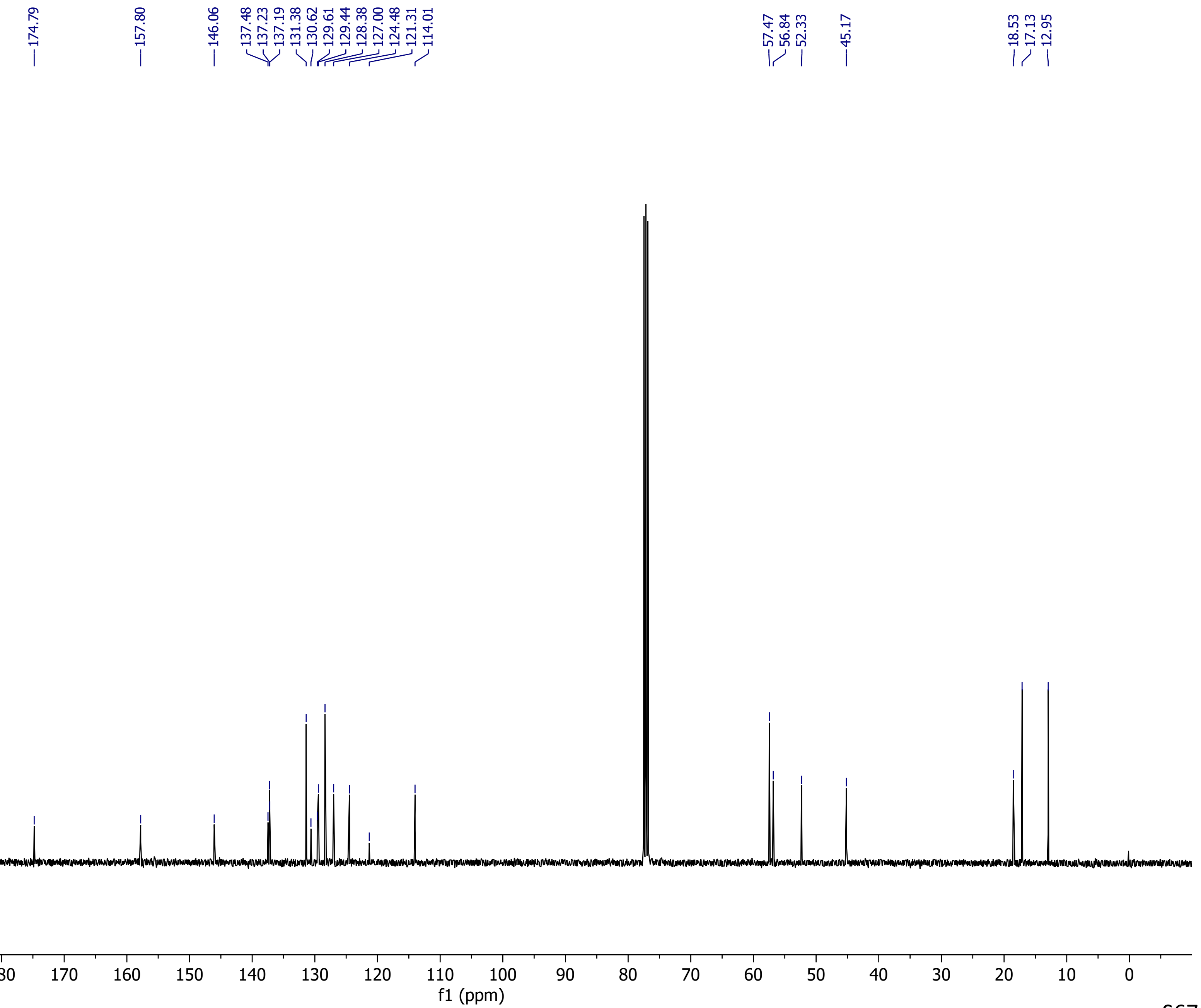

Supplement: Supplementary file 1 [file jo6c00744_si_001.pdf]
